# Structural Remodeling and Enzymatic Replacement Shape the Evolution of Organellar Group II Introns in *Ulva*

**DOI:** 10.3390/ijms27062613

**Published:** 2026-03-12

**Authors:** Feng Liu, Shuangle Jin, Huiyin Song

**Affiliations:** 1 Laboratory of Marine Ecology and Environmental Sciences, Institute of Oceanology, Chinese Academy of Sciences (IOCAS), Qingdao 266071, China; 2 Laboratory for Marine Ecology and Environmental Science, Qingdao Marine Science and Technology Center, Qingdao 266200, China; 3 College of Marine Science, University of Chinese Academy of Sciences, Beijing 101408, China; 4 School of Life Sciences, Jianghan University, Wuhan 430056, China

**Keywords:** group II introns, *Ulva*, organellar genomes, reverse transcriptase/maturase, LAGLIDADG homing endonuclease, RNA structure evolution, intron-protein coevolution

## Abstract

Group II introns are catalytic RNAs that combine self-splicing ribozyme activity with mobility and have played major roles in shaping organellar genome evolution. In green macroalgae of the genus *Ulva*, organellar genomes are highly compact, yet they harbor unusually diverse and dynamic repertoires of group II introns. To understand how organellar group II introns diversify and persist within compact organellar genomes, we performed a comparative analysis of mitochondrial and chloroplast group II introns across *Ulva*, integrating secondary structure reconstruction, intron occurrence patterns, and phylogenetic inference based on both conserved intron RNA regions and intron-encoded proteins (IEPs), including reverse transcriptase/maturase (RT/M) and LAGLIDADG homing endonuclease (LHE). A total of 168 mitochondrial and 123 chloroplast introns were identified and classified into 32 families belonging to seven major subgroups (IIA1-RT/M, IIA2-RT/M, IIB1-RT/M, IIB1-LHE, IIB2-RT/M, IIB2-LHE, and IIB-like). Most intron families retain the canonical six-domain architecture (DI–DVI), but four mitochondrial IIA families display a seven-domain configuration generated by the lineage-specific insertion of an additional stem-loop structure (DIIIa). Phylogenetic analyses revealed a high degree of congruence, supporting persistent coevolution between RNA scaffolds and their IEPs. Notably, the LHE-encoding families were scattered across distinct IIB lineages instead of forming a single clade, suggesting that at least two independent invasion events occurred within the IIB1 and IIB2 lineages. Analysis of intron occurrence frequency revealed an evolutionary continuum ranging from structurally intact and broadly distributed families to lineage-specific families exhibiting progressive scaffold degeneration, with the chloroplast *infA*-62 family representing a stably inherited lineage maintained through vertical transmission. These results suggest that organellar group II introns in *Ulva* evolve through coordinated scaffold remodeling, enzymatic replacement, and differential distribution patterns across genomic compartments, highlighting *Ulva* organellar genomes as a valuable comparative model for investigating the long-term evolution of mobile ribozymes within compact genomic environments.

## 1. Introduction

Group II introns are a distinctive class of catalytic RNAs that have attracted long-standing interest as model systems for investigating RNA catalysis and intron mobility within genomes [1,2]. Their RNA scaffolds adopt a conserved six-domain architecture (DI–DVI), within which structural and catalytic functions are partitioned rather than evenly distributed [3,4]. DI serves as the main scaffold for intron folding, DV houses the catalytic core, and DVI contains the branch-point adenosine required for lariat formation during excision [5,6]. This unusual combination of ribozyme activity and mobility has positioned group II introns as a central model system for dissecting RNA structure-function relationships [7,8] and for understanding the evolutionary transition from self-splicing introns to the spliceosomal machinery [6,9].

In algae, fungi, and plants, group II introns have played important roles in shaping organellar genome evolution [10]. They are typically protein-assisted rather than fully autonomous self-splicing elements [11,12], and their persistence is increasingly interpreted as being mediated by host splicing-factor networks [13,14]. In addition to their catalytic RNA components, many group II introns encode intron-encoded proteins (IEPs), most commonly reverse transcriptase/maturase (RT/M) proteins and, less frequently in mitochondrial genomes of some fungi and green algae [10,15], LAGLIDADG homing endonucleases (LHEs) [16]. RT/M proteins promote both splicing and mobility through their maturase activity and reverse transcription [17], whereas LHEs facilitate intron propagation by introducing site-specific DNA double-strand breaks [18,19]. Accumulating evidence from bacterial and organellar systems indicates that group II introns and their associated IEPs evolve as coupled RNA-protein units [20,21].

Mitochondria and chloroplasts usually exhibit compact, streamlined genomes, yet they often retain complex intron repertoires that can be lineage-specific and highly dynamic [22,23]. The evolutionary persistence of organellar group II introns is generally considered to reflect a balance between intron mobility, functional constraints on splicing, and progressive decay [11,12]. Active introns can spread via retrohoming mediated by conserved exon-binding site (EBS)/intron-binding site (IBS) interactions and functional IEPs [1,24]. However, degeneration of structural domains or IEPs can reduce mobility, favor persistence at specific loci, or ultimately lead to intron loss [19,20].

Green macroalgae of the genus *Ulva* (Ulvophyceae, Chlorophyta) are ecologically important coastal primary producers and are well known for forming large-scale green tides [25,26]. Recent studies have shown that both mitochondrial and chloroplast genomes in *Ulva* are compact but unusually dynamic, exhibiting extensive variation in genome architecture, gene order, and intron content. *Ulva* organellar introns are characterized by extensive family diversity, pronounced structural variability, and frequent gain and loss, even among closely related taxa (e.g., *Ulva prolifera* and *Ulva linza*) [22,23,26]. Previous work has documented the abundance of group II introns in *Ulva* organellar genomes [27,28], but these introns have largely been examined in isolation, rather than as components of an integrated evolutionary system.

Despite the growing number of available *Ulva* organellar genomes (Supplementary Tables S1 and S2), it remains unclear how group II introns diversify and persist over evolutionary time within these compact genomes. In particular, it is unknown whether organellar group II intron evolution is dominated by unidirectional degeneration or instead involves recurrent structural innovation, enzymatic replacement, and differential distribution patterns across genomic compartments. *Ulva* therefore provides a useful model system to examine how mobile ribozymes persist under extreme genome compaction. Here, we performed a comparative analysis of organellar group II introns across *Ulva* mitochondrial and chloroplast genomes, integrating intron family classification, secondary structure reconstruction, occurrence-frequency analysis, and phylogenetic inference based on both conserved intron RNA scaffolds and IEPs.

## 2. Results

### 2.1. Diversity and Structural Classification of *Ulva* Organellar Group II Introns

A total of 168 mitochondrial and 123 chloroplast group II introns were identified (Supplementary Tables S1 and S2) and classified into 32 intron families according to shared insertion sites and sequence homology among *Ulva* organellar genome sequences (Supplementary Table S3). A total of 20 mitochondrial intron families were defined, including 14 families encoding RT/M proteins, five families encoding LHEs, and one family (*cox1*-686), which was classified as an IEP-lacking IIB-LHE intron [26] based on the presence of a 5′ terminal linker sequence, which is characteristic of some IIB1-LHE introns [29]. A total of 12 chloroplast intron families were identified, including 10 families encoding RT/M proteins, one family (*orf185*-47) assigned as an IEP-lacking IIB2-RT/M intron and one IIB-like family (*infA*-62), which has completely lost its IEP [28].

**Figure 1:**
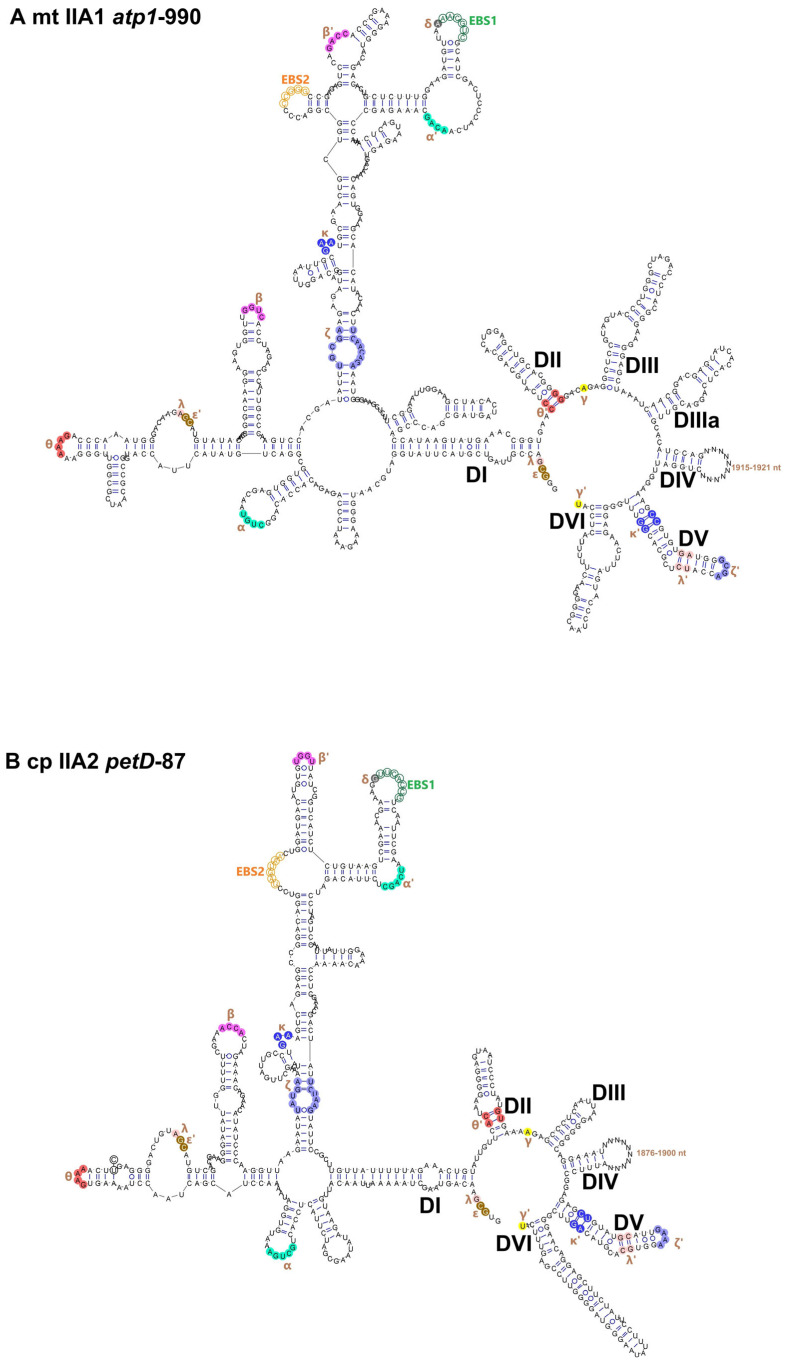

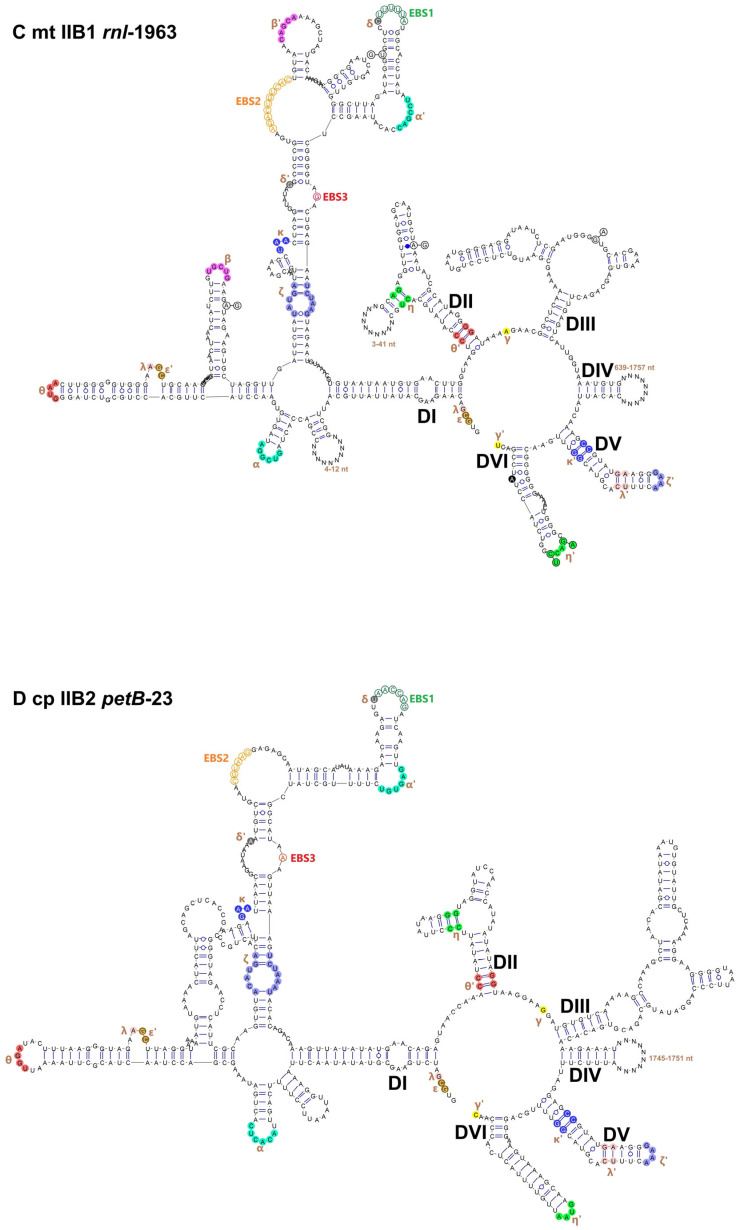
Secondary structure models of representative *Ulva* organellar group II introns. (**A**) Mitochondrial (mt) IIA1 intron *atp1*-990. (**B**) Chloroplast (cp) IIA2 intron *petD*-87. (**C**) mt IIB1 intron *rnl*-1963. (**D**) cp IIB2 intron *petB*-23. The sequences shown are representative constructs derived from sequence alignments. At each alignment site, the most frequently occurring nucleotide among family members is selected. When multiple nucleotides share the same highest frequency, they are denoted by black circles. For each intron, the canonical six domains (DI–DVI) are indicated. Exon-binding sites (EBS1/EBS2/EBS3) are annotated. Conserved tertiary interaction motifs are marked with Greek-letter labels (and primed partners) and shown in matching colors to denote long-range contacts within the ribozyme. DIV extensions correspond to variable regions that may accommodate additional sequence insertions typical of organellar group II introns.

Secondary structure reconstruction based on thermodynamic folding predictions and manual refinement showed that the vast majority of *Ulva* organellar group II introns conform to the canonical six-domain (DI–DVI) secondary structure model (Figure 1). However, four mitochondrial IIA intron families (*atp1*-990, *cox1*-643, *cox1*-760, and *nad5*-1057) exhibited an additional stem-loop structure inserted between DIII and DIV, resulting in a non-canonical seven-domain architecture (Figure 1A). This additional element, here designated DIIIa, represents a distinct structural elaboration within a subset of mitochondrial IIA families.

Intron classification was performed by integrating diagnostic secondary-structure features with the identities of their associated IEPs. Group IIA introns were subdivided into IIA1 and IIA2 subgroups based on characteristic junction motifs between DII and DIII (IIA1: BVGA; IIA2: RRGA) and additional conserved structural features [30]. Group IIB introns were assigned to IIB1 and IIB2 subgroups according to diagnostic motifs at the DIV-DV junction (IIB1: YW; IIB2: RG), the absence or presence of the specific domain Ia in the 5′ strand of domain I(i)/I(ii) (with noted lineage-specific exceptions), and other conserved structural sites [30]. With the exception of the previously described *infA*-62 family, which belongs to the IIB-like subgroup, all identified intron families could be assigned to six principal subgroups, namely IIA1-RT/M, IIA2-RT/M, IIB1-RT/M, IIB1-LHE, IIB2-RT/M, and IIB2-LHE (Supplementary Table S3). Across both mitochondrial and chloroplast genomes, IIB intron families were more abundant than IIA intron families.

### 2.2. Variations in Length and GC Content Among Organellar Group II Introns

**Figure 2:**
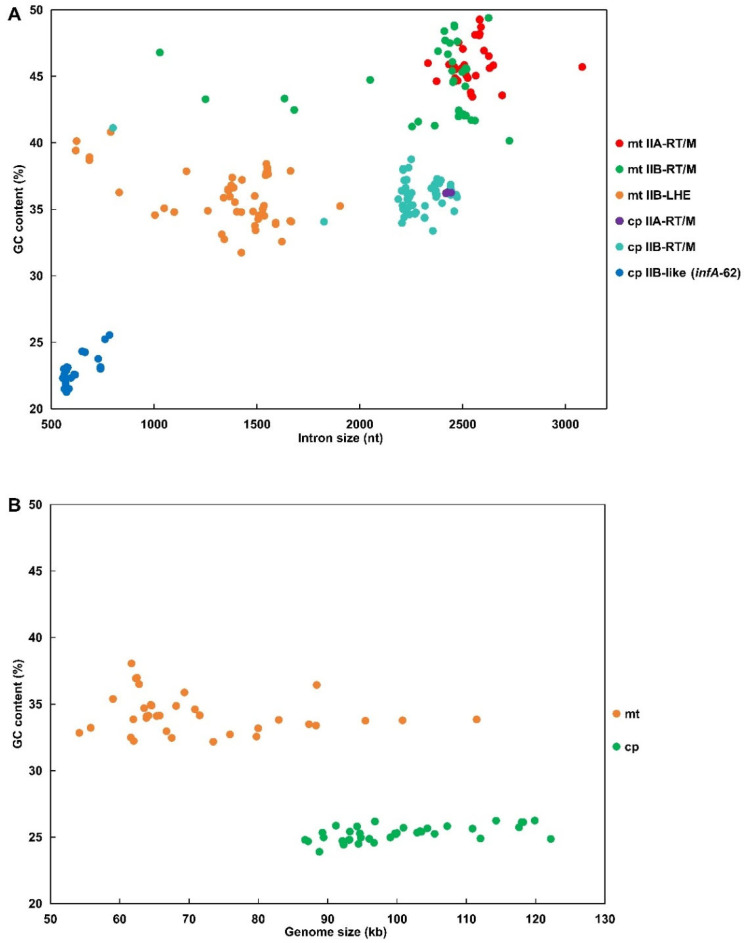
Relationships between size and GC content of group II introns and size and GC content of organellar genomes of *Ulva* species. (**A**) Scatter plot of intron length (nt) versus intron GC content (%) for different classes of organellar group II introns. Each dot represents one intron, colored by intron type: mitochondrial (mt) IIA-RT/M (red), mt IIB-RT/M (green), mt IIB-LHE (orange), chloroplast (cp) IIA-RT/M (purple), cp IIB-RT/M (cyan), and cp IIB-like introns (blue). (**B**) Scatter plot of organellar genome size (kb) versus genome GC content (%). Each dot represents one organellar genome and is colored by compartment: mitochondrion (mt; orange) and chloroplast (cp; green).

Marked differences in intron length were observed among intron lineages and between organellar compartments (two-way ANOVA, lineage effect *p* < 0.01; organelle effect *p* < 0.01; lineage × organelle interaction *p* < 0.01) (Figure 2A). Mitochondrial RT/M-encoding introns were the largest, with an average length of 2449 nt, whereas mitochondrial LHE-encoding introns were substantially shorter, with an average length of 1364 nt (Supplementary Table S4). Chloroplast RT/M-encoding introns occupied an intermediate size range with an average length of 2281 nt (Figure 2A). The chloroplast *infA*-62 family (IIB-like) was the shortest, with an average length of 615 nt (Supplementary Table S4), which is consistent with its highly degenerated structure [28]. In both mitochondrial and chloroplast genomes, the average length of IIA-RT/M introns was consistently longer than that of IIB-RT/M introns (*p* < 0.01, post hoc comparisons following two-way ANOVA) (Figure 2A). This difference is likely attributable to the presence of an intact RT/M open reading frame (ORF) in all IIA-RT/M introns, whereas the RT/M ORF in several IIB-RT/M introns showed varying degrees of degeneration.

Length variation among intron families was not only associated with concerted changes across structural domains (DI–DIV and DVI), but also involved the insertion of additional accessory domains. Several intron families displayed extreme size variation within specific domains. The *rnl*-2698 family possesses the most compact DI (257–287 nt) due to the complete absence of the domains IC2 and ID2 (Supplementary Table S5). The *cox1*-643 family exhibited the shortest DII (22 nt) among the II-RT/M families, and the *rnl*-2080 family contained the smallest DII (14–19 nt) among the II-LHE families. Four IIB2-RT/M families (*atpB*-627, *atpI*-256, *orf185*-47, and *petB*-69) and one IIA2-RT/M family (*petD*-87) contained a highly degenerated DIII (12–18 nt) (Supplementary Table S5). The *infA*-62 family harbored the shortest DIV (24–32 nt), while the 3081-nt *atp1*-1316, which was detected exclusively in a single *Ulva* species (MN853878), contained the longest DIV (2445 nt) among all families. Within most intron families, intrafamilial length variation was primarily driven by dramatic changes in DIV, particularly those associated with IEP degeneration. Some intron families showed lineage-specific divergence involving multiple domains. For instance, the *nad3*-216 family exhibited pronounced length variation (1634–2560 nt), largely attributable to the highly plastic DI (371–430 nt) and DIV (931–1864 nt) (Supplementary Table S5).

GC content analyses revealed clear lineage- and organelle-dependent patterns among group II introns (two-way ANOVA, lineage effect *p* < 0.01; organelle effect *p* < 0.01). Mitochondrial RT/M-encoding introns exhibited the highest GC content (avg. 45.56%), whereas chloroplast RT/M-encoding introns were consistently less GC-rich (avg. 36.09%) (Figure 2A). Mitochondrial LHE-encoding introns showed intermediate GC levels (avg. 35.55%) and the chloroplast *infA*-62 displayed the lowest GC content (avg. 22.73%). Most intron families displayed GC contents that differed substantially from those of their host organellar genomes (Figure 2B and Supplementary Table S4), suggesting partial decoupling between intron and host genome compositional evolution. The GC content of all RT/M-encoding intron families and nearly all LHE-encoding families (except *cox1*-874) was significantly higher than that of the host genome (*p* < 0.01). In contrast, the GC content of the chloroplast *infA*-62 family was lower than that of the chloroplast genomes (*p* < 0.01) (Figure 2 and Supplementary Table S4). These differences may reflect strong selection pressure toward AT enrichment in chloroplast introns, especially the *infA*-62 family. However, mitochondrial introns showed significantly different evolutionary patterns, and their compositional difference reflected a comprehensive result involving selective pressure, neutral decay, and modular enzymatic substitution.

### 2.3. Structural Remodeling of Group II Intron Domains

Comparative analyses of secondary structures revealed extensive structural plasticity across *Ulva* organellar group II introns, with DI representing the primary hotspot for structural remodeling. Multiple DI substructures exhibited lineage-specific loss, reduction, or modification. In contrast, the catalytic core domain (DV) remained highly conserved across all intron families, typically measuring ~34 nt in length, with only minor deviations observed in a few lineages (Supplementary Table S5).

**Figure 3:**
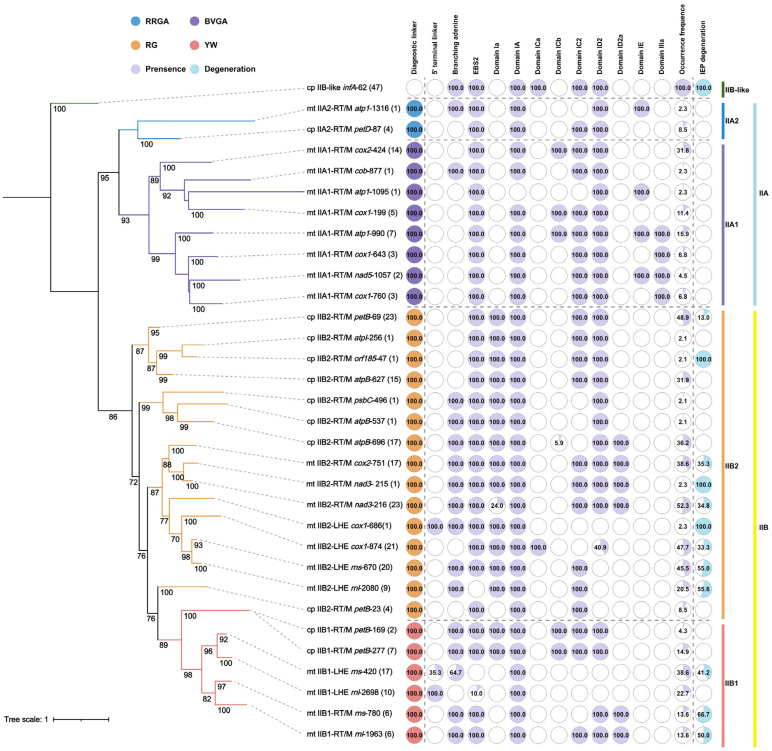
Phylogenetic relationships of organellar group II introns and comparative conservation of diagnostic structural features. A phylogenetic tree was reconstructed from alignments of conserved group II intron ribozyme regions (DI–DVI), with node support values (bootstrap, >70%) indicated at major branches. The scale bar represents substitutions per site. Terminal labels denote the organelle (cp, chloroplast; mt, mitochondrion), intron subgroup and IEP (RT/M or LHE, where present), host gene, and intron insertion site. Numbers in parentheses indicate the number of intron members in each intron family. Branches are colored according to the conserved splice-site/diagnostic motif class (RRGA, BVGA, RG, or YW; IUPAC codes where R = A/G, Y = C/U, W = A/U, B = G + C + U, V = A + G + C). To the right of the tree, a bubble matrix summarizes the occurrence and integrity of key structural/sequence elements across lineages, including diagnostic junction motifs, 5′ terminal linker, branch-point adenosine, EBS2, and structures within DI (Ia, IA, ICa, ICb, IC2, ID2, ID2a, and IE) as well as DIIIa. For each feature, the proportion (%) of the colored area within each bubble is proportional to the relative frequencies of its presence, occurrence, or degeneration in the corresponding lineage, and the percentage value is shown inside the circle. The right-side colored bars indicate intron subgroup assignments (IIA1, IIA2, IIB1, IIB2, and IIB-like).

Patterns of conservation differed markedly between intron types. All RT/M-encoding intron families retained a complete set of EBS tertiary interaction motifs, indicating strong conservation of the retrohoming recognition interface. In contrast, LHE-encoding families displayed heterogeneous patterns. Some families (e.g., *cox1*-874, *rns*-670, *cox1*-686) retained the full set of EBS motifs (EBS1–EBS3), whereas others (e.g., *rnl*-2080, *rns*-420 and 90.0% of *rnl*-2698) showed partial erosion of recognition motifs, most notably the loss of EBS2 in certain lineages (Figure 3). Similarly, domain Ia, typically regarded as a diagnostic feature of IIB2 introns, was retained in most IIB2 families but absent in several lineages, including the chloroplast *petB*-23 family and many members (76.0%) of the mitochondrial *nad3*-216 family. In addition, small Ia-like elements were detected sporadically in a few IIB1 intron families (*petB*-169 and *petB*-277) (Figure 3). These observations indicate that canonical diagnostic features of group II introns can undergo lineage-specific modification or erosion.

Loss and modification of DI substructures were widespread among intron families. Complete loss of domain IC2 occurred in several families of IIA1, IIA2, IIB1, and IIB2 lineages, while domain ID2 was absent in mitochondrial LHE-encoding families and in the chloroplast *petB*-23 family (Figure 3). In addition to domain loss, several lineages displayed structural insertions within DI. An additional stem-loop element (ID2a) was inserted between domains ID2 and ID3 in a subset of IIB1 and IIB2 families, whereas four mitochondrial IIA families (*atp1*-990, *atp1*-1095, *atp1*-1316, and *nad5*-1057) consistently contained an extra stem-loop structure (domain IE) inserted between domain ID and the terminal I(ii). These patterns highlight the modular nature of DI, which appears capable of accommodating both structural loss and lineage-specific innovation.

Terminal boundary motifs of introns were generally conserved but showed lineage-dependent variation. The canonical terminal motif (GUGCGA/AY) was largely maintained across most intron families, although mitochondrial IIA introns exhibited several substitutions restricted to the 5′ terminal motif, whereas the chloroplast IIA family *petD*-87 remained highly conserved at both termini (Supplementary Table S6). Most IIB-RT/M introns retained a conserved 5′ terminal motif, while several IIB-LHE and chloroplast intron families displayed modifications at one or both boundaries. A distinctive feature was observed in several mitochondrial IIB-LHE intron families (e.g., *cox1*-686, *rnl*-2698, and 35.3% of *rns*-420), in which a short 5′ terminal linker sequence was inserted between the upstream exon and the conserved 5′ terminal motif. The presence of this linker was consistently associated with loss of the canonical branch-point adenosine in DVI among mitochondrial IIB1-LHE intron families (Figure 3 and Supplementary Table S6). This coordinated modification suggests a structural signature linked to degeneration and reduced mobility in certain LHE-encoding lineages.

These lineage-specific modifications reveal an asymmetric pattern of constraint across the group II intron scaffold. The catalytic core domain (DV) is the most conserved element among *Ulva* organellar introns, whereas DI represents the principal hotspot of remodeling through recurrent loss, reduction, or insertion of peripheral substructures (e.g., IC2/ID2 loss and ID2a/IE/DIIIa insertions) (Figure 3). Structural variability differs among intron subclasses. RT/M-encoding introns consistently retain a complete EBS-IBS recognition interface, whereas LHE-encoding introns exhibit heterogeneous erosion of key motifs, most prominently the recurrent loss of EBS2. In mitochondrial IIB1-LHE lineages, this erosion is accompanied by the characteristic insertion of a 5′ terminal linker and the concurrent loss of the canonical branch-point adenosine in DVI. Additional lineage-specific signatures include pronounced DIII truncation in several IIB2-RT/M families and the presence of a derived DIIIa insertion shared by a subset of mitochondrial IIA1 introns. These patterns support a model in which a strongly conserved catalytic core is embedded within a permissive RNA scaffold whose peripheral domains can undergo both degeneration and innovation in a lineage- and IEP-dependent manner.

### 2.4. Phylogenetic Analysis Based on Conserved Intron RNA Sequences and Intron Occurrence Frequency

A phylogenetic tree was reconstructed using the maximum likelihood method based on conserved RNA sequences shared by *Ulva* organellar group II introns. The resulting topology clearly resolved these organellar intron families into three major evolutionary clades corresponding to IIA, IIB and IIB-like introns (Figure 3 and Supplementary Figure S1). The *infA*-62 family, representing an IIB-like lineage, was positioned as an outgroup, reflecting its highly divergent evolutionary status.

Within the IIA clade, four mitochondrial intron families characterized by a seven-domain (DIIIa-containing) architecture (*atp1*-990, *cox1*-643, *cox1*-760, and *nad5*-1057) tightly clustered together to form a well-supported subclade (Figure 3). Given that these four families contained the unique DIIIa, this topology supports a lineage-specific insertion event of DIIIa that occurred within the subclade. This subclade clustered with another subclade formed by four mitochondrial intron families (*atp1*-1095, *cob*-877, *cox1*-199, and *cox2*-424), tightly constituting the IIA1 lineage. The IIA1 lineage was resolved as a sister lineage to the IIA2 lineage comprising *atp1*-1316 and *petD*-87 (Figure 3).

Within the IIB clade, two mitochondrial II-LHE intron families (*rnl*-2698 and *rns*-420) and four mitochondrial II-RT/M families (*rnl*-1963, *rns*-780, *petB*-277, and *petB*-169) clustered together, forming the IIB1 lineage (Figure 3). The IIB2 lineage was composed of three chloroplast-derived and one mitochondria-derived subclades, which did not form an independent monophyletic clade. The phylogenetic topology supports the derivation of IIB2 introns from within the IIB1 lineage. The mitochondrial II-LHE intron families were divided into two subgroups: two (*rnl*-2698 and *rns*-420) in the IIB1 lineage and four (*cox1*-686, *cox1*-874, *rnl*-2080, and *rns*-670) in the IIB2 lineage. Four chloroplast IIB2-RT/M families (*atpB*-627, *atpI*-256, *orf185*-47, and *petB*-69), which shared an extremely degenerated DIII, clustered together, forming a well-supported subclade, indicating the small DIII was a lineage-specific characteristic of this subclade.

Analysis of intron occurrence frequency across *Ulva* species revealed substantial variation among intron families both within and among species, in sharp contrast to the stable presence of *infA*-62 across all sampled chloroplast genomes. Except for *infA*-62, the intron families with the highest occurrence frequencies (>40%) were exclusively group IIB2-RT/M introns, including *nad3*-216 (52.3%), *petB*-69 (48.9%), *cox1*-874 (47.7%), and *rns*-670 (45.5%) (Figure 3). By contrast, the RT/M-encoding intron families with low occurrence frequencies (<15%) were frequently accompanied by pronounced structural decay, such as the complete loss of domain IC2 in *atp1*-1095, *atp1*-1316, *rns*-780, *atpB*-537, and *psbC*-496, or the loss of domain ID2 in *petB*-23. All IIA1 introns harboring the specialized DIIIa exhibited relatively low occurrence frequencies (4.5–15.9%) (Figure 3). Organellar group II introns in *Ulva*, irrespective of whether they encode RT/M or LHE, retain dynamic distribution both within and among species, reflecting an evolutionary pattern of recurrent intron gain and loss.

**Figure 4:**
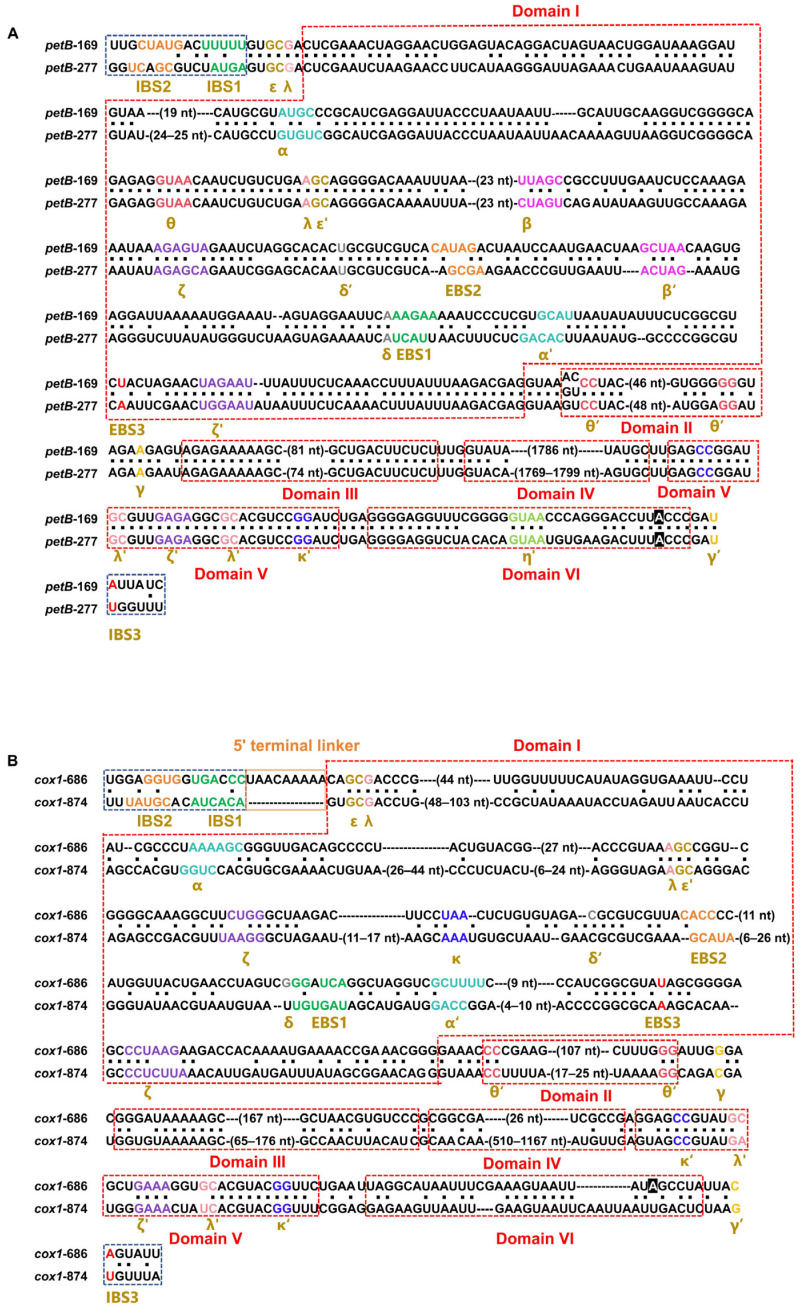
Comparative sequence-structure annotation of representative organellar group II intron families. (**A**) Pairwise alignment of chloroplast (cp) IIB1-RT/M introns, *petB*-169 and *petB*-277. (**B**) Pairwise alignment of mitochondrial (mt) IIB2-LHE introns, *cox1*-686 and *cox1*-874. For each panel, the intron RNA is annotated according to the canonical group II intron secondary structure model. Red dashed boxes delimit domains (DI–DVI). Exon-intron recognition elements are indicated, including IBS1/IBS2/IBS3 in flanking exons and the corresponding EBS1/EBS2/EBS3 in introns. Conserved tertiary interaction motifs are labeled with Greek letters (and primed partners) to denote long-range contacts. Dots between the two sequences indicate identical nucleotides, and numbers in parentheses denote the length of indel regions (nt) between aligned blocks. This comparison highlights conserved core motifs required for ribozyme function as well as lineage-specific insertions/deletions mainly located in peripheral regions of DI and DIV.

**Figure 5:**
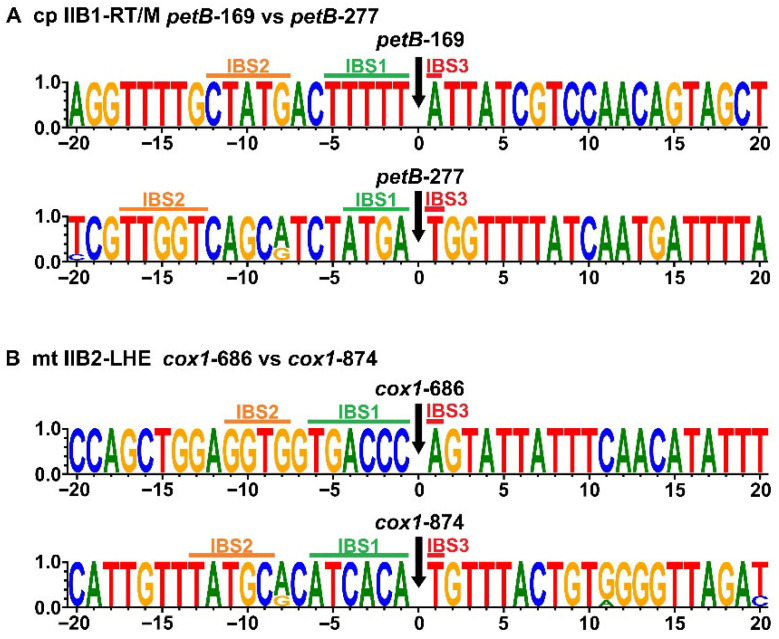
Exon-intron junctions of representative organellar group II introns highlighting IBS motifs and insertion-site sequences. The sequence logos show nucleotide conservation derived from alignments of exon sequences flanking the 5′ splice site (position 0; black arrow) within a 40-nt window (−20 to +20) surrounding the splice junction. Letter height reflects nucleotide frequency (information content), and colors denote the four nucleotides (A, C, G, and T). (**A**) Chloroplast (cp) IIB1-RT/M introns, *petB*-169 and *petB*-277. (**B**) Mitochondrial (mt) IIB2-LHE introns, *cox1*-686 (lacking an IEP) and *cox1*-874 (encoding an LHE). In each panel, nucleotide positions are numbered relative to the splice junction, where negative values indicate the upstream exon and positive values indicate the downstream exon. Green, orange and red bars mark the intron-binding sites IBS1, IBS2 and IBS3, respectively, which base-pair with the corresponding intronic exon-binding sites (EBS1, EBS2 and EBS3) during splice-site recognition.

The two chloroplast IIB1-RT/M families, *petB*-169 and *petB*-277, exhibited highly similar primary sequences and secondary structures but differed mainly at the EBS-IBS recognition interfaces (Figure 4A). Divergence was concentrated in the EBS regions, particularly EBS1 and EBS2 (Figure 5A), consistent with the recognition of distinct IBS targets (IBS1 and IBS2) and the occupancy of different genomic loci. Overall scaffold conservation coupled with interface-specific divergence suggests insertion-site switching between these closely related families.

The two mitochondrial IIB2 families, *cox1*-686 and *cox1*-874, show high similarity in structure (Figure 4B) and a close relationship in the phylogenetic tree (Figure 3). The *cox1*-686 has a 5′ terminal linker sequence, which is a typical characteristic of group IIB-LHE introns, but completely lost its IEP. Combined with their occurrence frequency and novel EBS variants, these data suggest that *cox1*-686 may have been derived from the *cox1*-874 lineage and inserted into another genomic locus, followed by complete IEP loss (Figure 5B).

### 2.5. Phylogenetic Analysis Based on Conserved IEP Sequences

**Figure 6:**
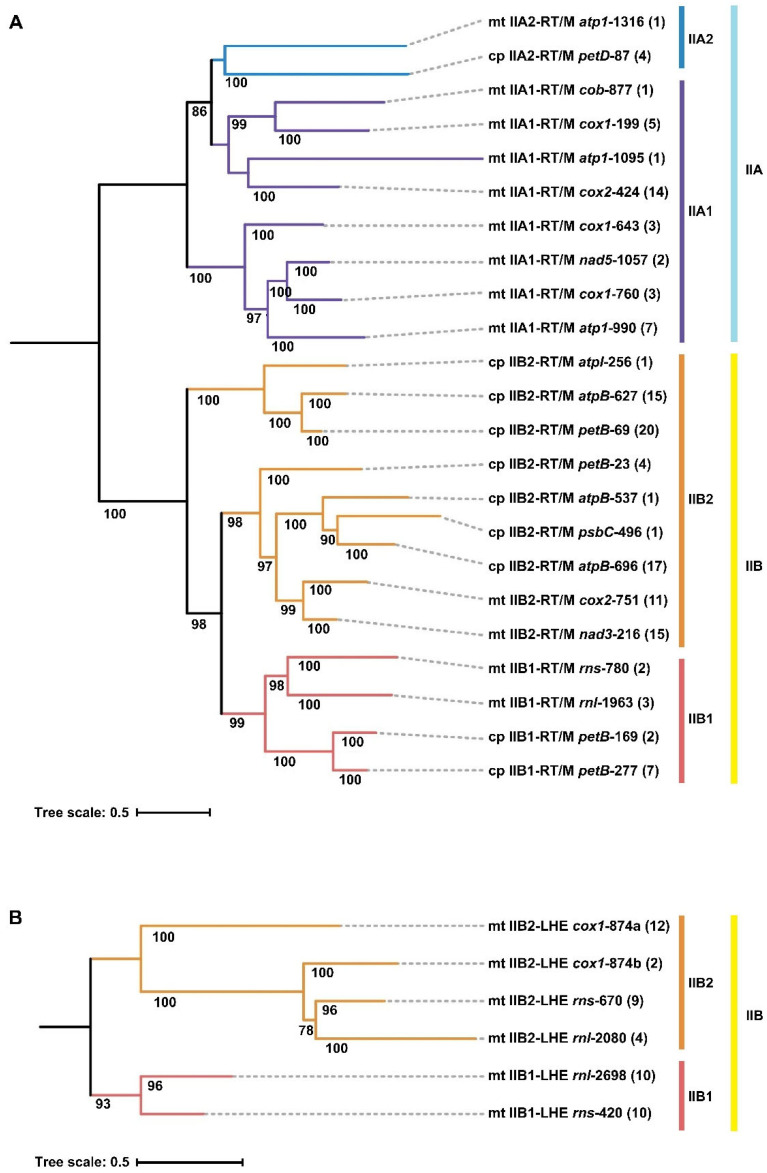
Phylogenetic classification of organellar group II intron families based on IEP sequences. Phylogenetic trees were reconstructed from sequence alignments of (**A**) the conserved RT/M proteins encoded by group II intron families from chloroplast (cp) and mitochondrial (mt) genomes, and (**B**) the conserved regions of LHEs encoded by group IIB intron families from mitochondrial genomes. Node support values (bootstrap, >70%) are indicated at major branches, and the scale bar represents substitutions per site. Terminal labels specify the organelle of origin, intron subgroup (IIA1, IIA2, IIB1, and IIB2), host gene, intron insertion site, and the number of intron members included in each family (in parentheses). Branch colors denote subgroup assignments, and the vertical bars on the right summarize intron subgroup membership.

All group IIA introns harbored an intact intron-encoded RT/M ORF, whereas some members of most group IIB intron families had a degenerated RT/M or LHE ORF, or even complete loss. No RT/M ORFs or recognizable RT/M remnants were detected in any IIB-LHE introns. The IIB introns in mitochondria tend to undergo degeneration more frequently than those in chloroplasts. Apart from *rnl*-2698, IEP degeneration was evident across all mitochondrial IIB families, whereas in chloroplast IIB families such progressive loss of IEP was restricted to *orf185*-47 and only some members of *petB*-69 (13.0%) (Figure 3). Phylogenetic analysis based on amino acid sequences of RT/M proteins revealed that RT/M proteins encoded by organellar group II introns in *Ulva* clustered into two clearly separated evolutionary clades corresponding to IIA and IIB introns (Figure 6A and Supplementary Figure S2). The overall topology of the IIA and IIB clades was largely consistent with the RNA-based phylogeny. These results supported a coevolutionary pattern between II-RT/M introns and their associated RT/M proteins within *Ulva* organellar genomes.

Phylogenetic analysis based on conserved amino acid sequences of LHEs revealed that LHEs encoded by IIB-LHE introns formed two well-supported clades, corresponding to IIB1 and IIB2, respectively (Figure 6B and Supplementary Figure S3). Phylogenetic analyses indicate that the IIB1 and IIB2 clades are sister clades to each other. This protein-based phylogenetic topology showed strong concordance with the phylogeny inferred from conserved intron RNA sequences, indicating that, following the formation of IIB-LHE introns, the intron RNA scaffolds and the associated LHE proteins displayed a coevolutionary pattern. Phylogenetic reconstruction indicated that the II-LHE families did not form a single independent evolutionary lineage. Instead, they were distributed in the IIB1 and IIB2 clades (Figure 6B), supporting a dual-origin scenario for mitochondrial IIB-LHE introns. One subgroup (*rnl*-2698 and *rns*-420) originated from an ancestral IIB1 lineage and shared a common ancestor with two IIB1-RT/M families (*rnl*-1963 and *rns*-780). The second subgroup (*cox1*-686, *cox1*-874, *rnl*-2080, and *rns*-670) originated from an ancestral IIB2 lineage and was homologous to the common ancestor of three IIB2-RT/M families (*cox2*-751, *nad3*-215, and *nad3*-216). These results support a model in which at least two distinct LHE genes independently invaded the RNA scaffolds of IIB1 and IIB2 introns during evolution.

Overall, the congruent RNA- and protein-based phylogenies, together with the dual origin of IIB-LHE introns, support coevolution of intron RNA scaffolds and IEPs and provide a phylogenetic framework for discussing repeated IEP invasion, degeneration, and turnover in *Ulva* organellar genomes.

## 3. Discussion

### 3.1. Modular Remodeling of DI and Variation at the Recognition Interface Between EBS and IBS

**Figure 7:**
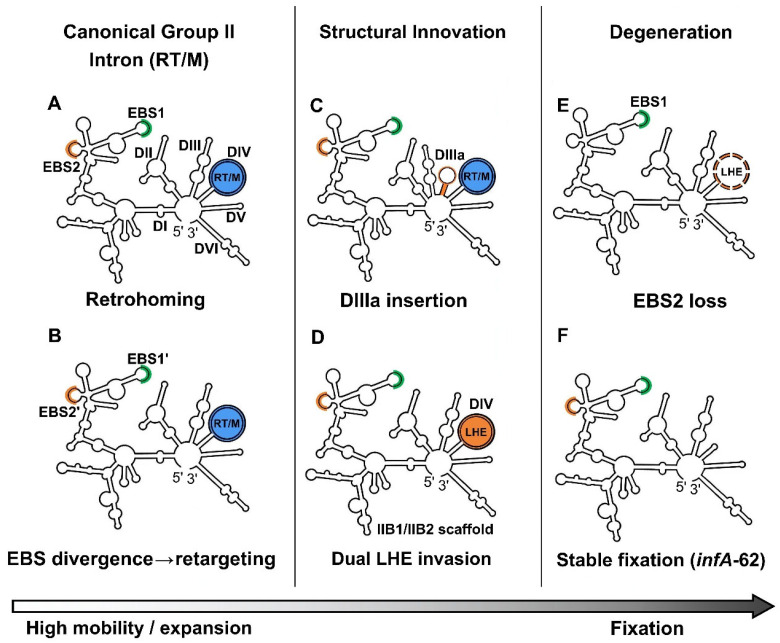
An inference-based model summarizing evolutionary states of organellar group II introns in *Ulva*. This model is intended to guide interpretation rather than to imply a single obligatory evolutionary trajectory. The diagrams summarize representative structural and functional configurations observed among *Ulva* organellar group II introns, arranged along a gradient reflecting decreasing mobility and increasing structural degeneration. Conserved ribozyme domains (DI–DVI) are indicated where relevant. Colored circles denote exon-binding sites (EBS1, green; EBS2, orange) and encoded protein modules (RT/M, blue; LHE, orange). Diagrams are not drawn to scale. (**A**) A canonical RT/M-encoding intron state characterized by intact ribozyme architecture and efficient retrohoming capacity. (**B**) A retargeting-prone state marked by divergence of EBS1 and EBS2. (**C**) A structurally modified RT/M-encoding intron retaining catalytic competence but exhibiting peripheral domain innovation (e.g., DIIIa insertion). (**D**) An alternative intron configuration in which an LHE is associated with IIB1 or IIB2 scaffolds, consistent with enzymatic replacement or invasion events. (**E**) The degeneration of the LHE-encoding introns manifested by the loss of EBS2, as well as a certain degree of LHE ORF decay. (**F**) A low-mobility, host-accommodated intron state lacking an IEP, exemplified by the chloroplast *infA*-62 family, representing one possible long-term outcome rather than a universal endpoint.

To integrate the distributional, structural, and motif-level patterns revealed by our comparative analyses, we interpret *Ulva* organellar group II intron families along a continuum of evolutionary states ranging from interface-driven retargeting and episodic spread to progressive degeneration and, in some cases, long-term host accommodation (Figure 7). This schematic framework is intended as a heuristic summary rather than a single deterministic pathway, and the canonical RT/M-encoding configuration is used as a practical reference for describing modular remodeling and IEP turnover (Figure 7A).

Within this framework, DI ranks among the most structurally variable regions across *Ulva* organellar introns. Recurrent modification or loss of DI substructures (e.g., IC2 and ID2) is observed across multiple lineages, highlighting DI as a major hotspot of scaffold remodeling. This variability does not imply instability of the ribozyme as a whole; instead, it is consistent with a conserved catalytic core supported by a permissive RNA scaffold that tolerates divergence in peripheral regions. Because DI mediates long-range tertiary contacts and provides interaction surfaces for both IEPs and host splicing factors [31,32], structural flexibility at this domain may facilitate persistence of introns across distinct organellar contexts without disrupting core splicing chemistry.

Variation at the EBS-IBS recognition interface provides an additional axis for intron diversification by enabling locus turnover without extensive changes to the overall scaffold. The two chloroplast IIB1-RT/M families, *petB*-169 and *petB*-277, remain highly similar in overall RNA scaffold organization yet differ substantially in their EBS motifs, particularly EBS1 and EBS2 within domain ID (Figure 7B). Because base-pairing between EBS and IBS motifs determines target-site recognition during retrohoming, this pattern is consistent with an “interface-first” mode of diversification in which the catalytic scaffold remains largely conserved while the recognition interface evolves rapidly to access new IBS targets [3]. A direct prediction of this model is that newly colonized loci should exhibit IBS signatures complementary to derived EBS variants, which can be evaluated through insertion-site sequence logos [1]. These observations support a jumping-degeneration continuum in which interface innovation promotes insertion-site switching and spread, whereas progressive structural decay constrains mobility and predisposes lineages to contraction or long-term fixation in organellar genomes [19,33].

Motif conservation patterns across intron subclasses further support this interpretation. The uniform retention of EBS1 and EBS2 in RT/M-encoding introns is consistent with strong constraints on the canonical EBS-IBS interface required for retrohoming and efficient splice-site definition. In contrast, the recurrent erosion of EBS2 in LHE-encoding lineages suggests relaxed constraints on retrohoming-type recognition, potentially reflecting a shift toward endonuclease-assisted mobility and/or reduced mobility. Moreover, the consistent co-occurrence of a 5′ terminal linker with loss of the canonical branch-point adenosine in mitochondrial IIB1-LHE introns indicates coordinated remodeling of exon-intron boundaries and lariat-formation signals, which may alter splicing pathways or increase dependence on host factors (Figure 3 and Figure 7).

These DI- and interface-centered signatures provide a mechanistic basis for the evolutionary continuum (Figure 7) and motivate the clade-specific structural innovation described in Section 3.2 as well as the modular replacement and degeneration of IEPs discussed in Section 3.3.

### 3.2. The Seven-Domain Secondary Structure Configuration Generated by a Lineage-Specific DIIIa Insertion

Despite an evolutionary trend toward compaction in *Ulva* organellar genomes, most group II introns examined here retain the canonical six-domain (DI–DVI) secondary-structure organization, indicating strong conservation of the overall ribozyme architecture among these organellar introns [4,34]. Nevertheless, lineage-specific structural innovations were also detected. Notably, four mitochondrial IIA1-RT/M families (*atp1*-990, *cox1*-643, *cox1*-760, and *nad5*-1057) harbor an additional accessory stem-loop element, designated DIIIa, inserted between domains DIII and DIV (Figure 7C).

Atypical bacterial group II introns with an extra 3′ extension downstream of DVI have been reported, and two stem-loop substructures have been described as part of this extension in the *Bacillus cereus* group [5]. However, based on our literature survey, we found no previous reports describing a DIIIa-like insertion positioned between DIII and DIV in organellar group II introns. We therefore interpret DIIIa as a lineage-specific mode of structural remodeling in organellar introns.

Phylogenetic analyses based on conserved intron RNA sequences further showed that these four DIIIa-containing intron families form a well-supported subclade within the IIA1 lineage, most parsimoniously consistent with a single ancestral DIIIa insertion followed by vertical inheritance rather than multiple independent gains. This pattern indicates that, alongside degeneration and domain reduction, organellar introns can also diversify via discrete acquisition of peripheral structural elements.

The functional consequences of the DIIIa insertion remain uncertain. Domain III participates in tertiary interactions and can influence folding pathways [31,32]. Insertion of an accessory stem-loop at the DIII-DIV junction could plausibly affect local folding dynamics or scaffold stability [33]. Importantly, this modification occurs outside the catalytic core (DV), which remains highly conserved in both length and sequence across the introns analyzed [4,34]. We therefore view the DIIIa-containing introns as a derived configuration in which catalytic capacity is maintained while peripheral remodeling proceeds.

Notably, these DIIIa-containing IIA1 families occur at relatively low frequencies (4.5–15.9%) in our dataset. Interpreting occurrence frequency as a long-term proxy that integrates invasion, retention, and loss (see Section 3.4), this pattern is consistent with limited spread and/or reduced persistence relative to broadly distributed families. The DIIIa insertion illustrates that organellar group II introns can undergo lineage-specific structural innovation beyond simple degeneration [35]. Future functional assays will be required to test whether DIIIa alters splicing efficiency, mobility, or dependence on host factors.

### 3.3. Evidence for Two Independent Origins of Mitochondrial IIB-LHE Introns

A central evolutionary inference from our phylogenetic analyses is that mitochondrial IIB-LHE introns in *Ulva* originated through at least two independent acquisition events rather than from a single monophyletic II-LHE lineage. This conclusion is supported by the combined topology of RT/M- and LHE-based phylogenies, together with their concordance with RNA scaffold relationships (Figure 6).

RT/M phylogenies show that RT/M proteins encoded by organellar group II introns cluster into two clearly separated clades corresponding to IIA and IIB introns. The topology of the IIA clade is fully congruent with that inferred from conserved intron RNA sequences, whereas the IIB clade shows broad, though not complete, agreement with the RNA-based phylogeny. Notably, the RNA-based analysis excludes all IEP-encoding regions, so the overall concordance between RNA- and protein-based trees supports a long-term coevolutionary association between intron RNA scaffolds and RT/M proteins [3,30]. Across *Ulva* organellar genomes, degeneration of IEPs is more common in IIB introns than in IIA introns, and this tendency is especially pronounced in mitochondrial IIB introns relative to their chloroplast counterparts, consistent with different selective regimes across the two organellar contexts.

LHE phylogenies resolve two well-supported clades corresponding to IIB1-LHE and IIB2-LHE introns. These protein-based groupings closely mirror the classification of their associated RNA scaffolds, suggesting that, following LHE acquisition, LHEs and intron RNAs evolved in a coupled manner [36,37]. Importantly, the II-LHE introns do not form a single monophyletic lineage; instead, they are distributed across both the IIB1 and IIB2 clades. The most parsimonious explanation is therefore that at least two distinct LHE genes independently invaded pre-existing IIB1 and IIB2 RNA scaffolds (Figure 7D). In addition, the presence of LHE-encoding IIB introns in three mitochondrial genes (*cox1*, *rnl*, and *rns*) suggests subsequent proliferation after these independent recruitment events, extending beyond the more restricted distributions reported for LHE-encoding IIB introns in other systems [3,38].

In the *Ulva* organellar genomes examined here, no complete RT/M ORF or recognizable RT/M remnants were detected in any IIB-LHE intron. This pattern is consistent with LHE acquisition occurring in lineages where the canonical RT/M-driven retrohoming machinery had already been inactivated or lost, although highly degraded remnants cannot be entirely excluded [19,37]. Consistent with this interpretation, RT/M-encoding intron families retain a complete set of EBS motifs, whereas EBS2 loss is observed only in some LHE-encoding families (Figure 7E). Mechanistically, replacement of RT/M with an LHE implies a shift in mobility strategy: retrohoming depends on functional RT/M proteins and intact EBS-IBS interactions [33,39], whereas LHEs can promote intron spread via site-specific DNA cleavage followed by repair-mediated integration [19,40]. Under this model, acquisition of an LHE may provide an alternative route for persistence or limited expansion once RT/M function becomes compromised. More broadly, the independent recruitment of LHEs into two distinct IIB scaffolds highlights group II introns as evolvable RNA platforms capable of accommodating different enzymatic modules [37]. The dual-origin pattern of IIB-LHE introns and the associated motif/ORF changes support modular enzyme replacement as a recurrent evolutionary mechanism in *Ulva* mitochondria.

### 3.4. Occurrence Frequency of Intron Families as a Proxy for Evolutionary State

Occurrence frequency of intron families varies greatly among *Ulva* organellar genomes, reflecting a dynamic history of intron gain and loss across both mitochondrial and chloroplast lineages [1,28]. In the framework proposed here (Figure 7), we interpret occurrence frequency not as a direct measure of current intron mobility but as a long-term proxy integrating invasion success, vertical persistence, and lineage-specific loss across the genus *Ulva*. Although indirect, this metric provides an empirical basis for comparing evolutionary trajectories among different intron families.

With the exception of the chloroplast *infA*-62, most intron families that are broadly distributed across species (>40% of sampled genomes) belong to IIB2 lineages, including *nad3*-216, *petB*-69, *cox1*-874, and *rns*-670. In contrast, many low-frequency RT/M-encoding families (<15%) show clear signatures of structural deterioration, most commonly involving loss of DI elements such as IC2 or ID2. The association between restricted occurrence frequency and RNA scaffold degeneration is consistent with a scenario in which progressive domain loss reduces intron mobility, limits further transmission, and ultimately predisposes lineages to contraction and eventual loss from organellar genomes [3].

Across the dataset, intron families therefore appear to span a continuum of evolutionary states. At one end are structurally intact lineages with broad distributions and relatively high transmission potential. At the other are late-stage introns characterized by reduced occurrence frequency, structural degeneration, and limited mobility. These distributional patterns complement the structural and motif-level signatures discussed above and collectively support the jumping-degeneration continuum (Figure 7).

An exception to this pattern is the chloroplast *infA*-62 family. Despite extensive structural reduction and complete loss of an IEP, this intron is stably retained across *Ulva* chloroplast genomes and shows clear evidence of compositional convergence toward the host genome [28]. Rather than reflecting recent mobility, this pattern is consistent with an alternative evolutionary regime in which reduced mobility facilitates long-term fixation at a specific genomic locus, promoting host accommodation rather than repeated spread [9]. These observations indicate that occurrence frequency captures the cumulative outcome of invasion, degeneration, and retention processes acting over evolutionary time.

### 3.5. Contrasting Mitochondrial and Chloroplast Regimes in Group II Intron Evolution

Intron size, scaffold remodeling, IEP turnover, and GC landscapes indicate that *Ulva* mitochondria and chloroplasts impose distinct evolutionary regimes on group II introns, consistent with the evolutionary continuum (Figure 7). In broad terms, mitochondrial introns show signatures of higher turnover and repeated invasion-degeneration cycles, whereas chloroplast introns more often exhibit longer-term retention and, in specific cases, host accommodation.

One axis of this contrast is intron size evolution. Differences in intron length between organellar compartments can be partly attributed to IEP type. RT/M ORFs embedded within DIV contribute substantially to intron length [19], whereas LHE ORFs are considerably shorter, producing much shorter II-LHE introns [37,38]. However, coding capacity alone does not fully explain size diversity. Intrafamilial length variation is frequently dominated by large changes in DIV, consistent with progressive degeneration of IEPs as an important contributor to intron contraction over evolutionary time [1,3]. In addition, extensive remodeling of RNA structural domains contributes to size change, including pronounced reductions in specific domains (e.g., near-minimal DII in *cox1*-643 and *rnl*-2080; severely truncated DIII in *atpB*-627, *atpI*-256, *orf185*-47, *petB*-69, and *petD*-87) as well as localized expansions such as insertion of an Ia-like element in *petB*-169 and *petB*-277. These patterns reinforce the view of group II introns as modular RNA architectures in which scaffold remodeling and IEP degeneration jointly shape intron size and structural organization.

A second axis is compositional evolution. Mitochondrial RT/M-encoding introns show pronounced GC enrichment relative to their chloroplast counterparts, suggesting distinct compositional and selective environments for intron evolution in mitochondria versus chloroplasts [39]. In many cases, intron GC contents deviate substantially from those of surrounding host genomes, consistent with partially decoupled compositional evolution and, in some instances, with recurrent invasion or horizontal transmission histories [3,39]. GC enrichment in mitochondrial RT/M-encoding introns may also reflect structural constraints associated with maintaining stable RNA secondary and tertiary interactions required for ribozyme folding and activity, because GC-rich sequences can enhance base-pairing stability and support long-range interactions within the intron scaffold [3,28]. By contrast, the chloroplast *infA*-62 family exhibits GC composition closely matching the chloroplast genome, pointing to compositional amelioration toward the host genome [28]. The compositional convergence of *infA*-62, together with its stable retention across species, is consistent with a domestication-like evolutionary trajectory (Figure 7F), in which reduced mobility and long-term persistence allow gradual structural and compositional integration into the host genome [9,28]. Here, “domestication-like” denotes fixation and compositional accommodation rather than functional co-option or recruitment [9].

Viewed together with the structural and protein-level patterns described above, these organelle-specific contrasts support a coherent evolutionary interpretation. Mitochondrial genomes appear to favor higher intron turnover, involving repeated cycles of invasion, enzymatic replacement or degeneration, and eventual loss, whereas chloroplast genomes appear to permit longer-term retention and host accommodation in specific intron families [12]. This contrast provides a useful comparative system for considering how genomic context shapes intron persistence, degeneration, and the potential for long-term integration within host organellar genomes [9,12].

The analyses presented here establish a robust comparative system for examining organellar group II intron diversification in *Ulva*, but several limitations should be noted. Firstly, our inferences are based primarily on secondary-structure reconstruction, occurrence frequency, and phylogenetic analyses, and direct experimental tests of splicing efficiency and protein activity were not performed [9,11]. Secondly, divergence at the EBS-IBS interface provides a plausible explanation for target-site switching, but experimental validation of altered IBS specificity will be required to confirm this mechanism [19,33]. Finally, broader taxon sampling across Ulvophyceae is likely to refine estimates of the timing and frequency of enzymatic invasion events.

## 4. Materials and Methods

### 4.1. Dataset Construction and Family Naming of Group II Introns

Organellar genome data of *Ulva* species were obtained from a curated dataset previously established by our laboratory and a systematic retrieval of publicly available sequences from the NCBI GenBank database (as of 31 January 2026). A total of 44 mitochondrial and 47 chloroplast genome sequences of *Ulva* species were included in the final dataset. All genome accession numbers used in this study are provided in Supplementary Tables S1 and S2. To ensure the accuracy and completeness of the data, all organellar genomes were checked and reannotated (e.g., using organelle-focused annotation workflows such as MFannot) [41]. Group II introns were validated using RNAweasel (https://megasun.bch.umontreal.ca/RNAweasel/; accessed on 7 July 2025) to confirm hallmark structural features of group II introns, including a conserved DV [42,43]. Mitochondrial and chloroplast introns were named according to their host genes and insertion sites, using homologous genes from the *Ulva compressa* mitochondrial genome (KY626327) and chloroplast genome (MW353781) as references, respectively. The *orf185*-47 family, which was only detected in the chloroplast genome of *Ulva tepida* (OL684341), was named according to its insertion site in *orf185*. Introns occurring at identical insertion sites of host genes and showing sequence homology were defined as belonging to the same intron family [22,23]. Mitochondrial and chloroplast intron datasets were constructed separately, including organelle type, species name, intron content, and GenBank accession numbers. Using NCBI ORF Finder, ORFs were identified within DIV of IEP-encoding introns, and amino acid sequences were extracted for full-length RT/Ms and LHE proteins, respectively. An IEP was considered intact if a continuous ORF covered the conserved RT/M (or LHE) core regions without premature stop codons. Otherwise, it was classified as degenerated [29].

### 4.2. Secondary Structure Construction and Comparative Analyses of Group II Introns

Secondary structure prediction was performed to reconstruct representative intron structures and key interaction sites. To capture typical tertiary interaction motifs within each intron family, a custom R script (available upon reasonable request) was used to generate a representative sequence by selecting, at each alignment position, the nucleotide with the highest frequency among members of each intron family. RNA secondary structures were reconstructed using RNAstructure [44] and the UNAFold web server [45,46], combined with manual adjustment, with particular emphasis on the folding of the core domains (DI–DVI) and the identification of key tertiary interaction sites. Predictions focused on preserving known group II intron architectural constraints rather than de novo folding. Subsequently, structural comparison and diversity assessment were carried out at two levels. Within each intron family, secondary structures of different members were compared to identify conserved base-pairing regions and variable loop regions. Between families, we systematically compared structural characteristics to evaluate divergence in domain size, GC content, domain gain/loss events, and variation in IEPs (typically located in DIV). All predicted secondary structures were visualized using VARNA v3-93 [47]. Intron families were classified using both diagnostic secondary structural features [30] and the identities of their associated IEPs. Differences in intron length and GC content were tested using two-way ANOVA with intron lineage and organellar compartment as fixed factors, followed by Tukey’s HSD post hoc tests.

### 4.3. Occurrence Frequency of Group II Intron Families

To characterize intron distribution patterns, we quantified occurrence frequency at the level of the genus *Ulva* [28]. For each intron family, homologous searches and precise identification were performed using BLASTN, with known intron boundary sequences and/or conserved structural regions as probes [48]. BLASTN searches were performed with an E-value cutoff of 1 × 10^−5^ and minimum query coverage of 30% (or as appropriate). Presence-absence patterns across species and organellar genomes were summarized to calculate the occurrence frequency and distribution breadth for each intron family. Families present in >40% of sampled organellar genomes of *Ulva* species were considered broadly distributed, whereas those present in <15% were considered narrowly distributed. Insertion-site analyses were subsequently conducted by extracting ~20 nt of genomic DNA sequence upstream and downstream of each intron insertion site from the corresponding annotated host gene region. Flanking sequences from all members of the same intron family were aligned using MEGA v11.0 [49], and sequence logos were generated with WebLogo v3 to visualize nucleotide conservation and consensus patterns at insertion sites [50].

### 4.4. Phylogenetic Analysis Based on Conserved Intron RNA Sequences

For phylogenetic reconstruction, highly conserved regions corresponding to the catalytic core of group II intron RNAs were manually identified, extracted, and curated from all intron sequences. To reduce alignment ambiguity and minimize the impact of lineage-specific structural variability, a total of 12 mutation-hotspot regions were excluded prior to analysis. These excluded regions comprised the 5′ terminal sequence (present only in a subset of IIB-LHE introns); loop regions within domain IA; regions corresponding to domains Ia, ICa, and ICb; IC2; ID2 together with its adjacent connecting regions; IDa; the DIIIa region (restricted to certain mitochondrial introns); and loop regions within DII, DIII, and DIV. After curation, 793 nucleotide positions were retained and used for phylogenetic inference. Maximum-likelihood (ML) trees were reconstructed using IQ-TREE v3.0.1 [51]. The optimal nucleotide substitution model was selected using ModelFinder [52], which identified GTR + F + I + G4 as the best-fitting model. Branch support was evaluated using both 1000 ultrafast bootstrap replicates (UFBoot2) [53] and 1000 SH-like approximate likelihood ratio tests (SH-aLRT) [54]. Resulting phylogenetic topologies were examined to assess whether intron families cluster according to host gene identity, organelle origin, or species relationships, thereby providing insight into potential evolutionary trajectories, including vertical inheritance and horizontal transfer.

### 4.5. Phylogenetic Analyses Based on RT/Ms and LHEs

To evaluate evolutionary coupling between intron RNAs and their associated IEPs, phylogenetic analyses were conducted separately for RT/M and LHE proteins. Because a proportion of intron sequences showed varying degrees of degeneration in IEPs, only sequences retaining sufficient length and recognizable conserved ORFs were included. In total, 140 RT/M protein sequences and 47 LHE protein sequences were retrieved for analysis. Protein sequences were aligned at the amino acid level using MUSCLE as implemented in MEGA v11.0 [49,55]. Maximum-likelihood phylogenies were reconstructed using IQ-TREE v3.0.1 [51]. For RT/M proteins, the alignment consisted of 140 sequences spanning 681 amino acid positions. ModelFinder was used to select Q.PFAM + F + I + G4 as the optimal substitution model [52], and node support was assessed using 1000 ultrafast bootstrap replicates [53] and 1000 SH-aLRT tests [54]. For LHE proteins, 47 sequences comprising 193 aligned amino acid positions were analyzed. ModelFinder was employed to select VT + G4 as the best-fitting model [52], and support values were likewise assessed using 1000 ultrafast bootstrap replicates [53] and 1000 SH-aLRT tests [54]. To infer coevolutionary relationships, phylogenetic topologies derived from conserved intron RNA sequences were compared directly with those obtained from RT/M and LHE protein datasets. Patterns of congruence and conflict among the three trees were examined in detail to assess evolutionary coupling between intron RNA scaffolds and their IEPs and to infer key evolutionary events such as coordinated divergence, protein loss, or modular enzymatic replacement. All analyses were performed using established bioinformatic software with fixed parameters as specified, ensuring methodological reproducibility of the results.

## 5. Conclusions

This study presents the first genus-wide comparative analysis of organellar group II introns in the green macroalgal genus *Ulva*. By integrating RNA secondary structure reconstruction, intron occurrence patterns, and phylogenetic analyses of both intron RNA scaffolds and IEPs, we provide a comprehensive framework for understanding the evolutionary dynamics of these mobile ribozymes in compact organellar genomes. Our results demonstrate that *Ulva* organellar group II introns retain a highly conserved catalytic core while exhibiting extensive lineage-specific variation in peripheral RNA domains and associated enzymatic components. Structural analyses revealed recurrent remodeling of RNA scaffolds, particularly within DI, as well as lineage-specific innovations such as the DIIIa insertion in a subset of mitochondrial IIA1 introns. Phylogenetic analyses further support strong coevolution between intron RNAs and their IEPs and indicate that mitochondrial IIB-LHE introns originated through at least two independent acquisitions of LHE. Patterns of intron occurrence frequency and structural integrity suggest that individual intron families occupy different positions along an evolutionary continuum ranging from structurally intact and widely distributed lineages to highly degenerated, low-mobility introns. In addition, comparisons between mitochondrial and chloroplast genomes reveal contrasting evolutionary regimes, with mitochondrial introns showing more frequent structural remodeling and IEP turnover, whereas chloroplast introns more often exhibit long-term retention and, in some cases, host accommodation. These findings indicate that organellar group II introns in *Ulva* evolve through multiple, non-exclusive evolutionary trajectories shaped by RNA scaffold plasticity, enzymatic replacement, and organellar genomic context. The comparative framework established here highlights *Ulva* organellar genomes as a valuable model system for investigating the long-term evolution of mobile ribozymes in compact genomes.

## Data Availability

The original contributions presented in this study are included in the article. Further inquiries can be directed to the corresponding author.

## Supplementary Materials

The following supporting information can be downloaded at https://www.mdpi.com/article/10.3390/ijms27062613/s1.

## References

[1] Lambowitz A.M., Zimmerly S. (2011). Group II Introns: Mobile Ribozymes That Invade DNA. Cold Spring Harb. Perspect. Biol..

[2] Costa M. (2022). Group II Introns: Flexibility and Repurposing. Front. Mol. Biosci..

[3] Zimmerly S., Semper C. (2015). Evolution of Group II Introns. Mob. DNA.

[4] Toor N., Keating K.S., Taylor S.D., Pyle A.M. (2008). Crystal Structure of a Self-Spliced Group II Intron. Science.

[5] Tourasse N.J., Stabell F.B., Kolstø A.-B. (2010). Structural and Functional Evolution of Group II Intron Ribozymes: Insights from Unusual Elements Carrying a 3′ Extension. New Biotechnol..

[6] Keating K.S., Toor N., Perlman P.S., Pyle A.M. (2010). A Structural Analysis of the Group II Intron Active Site and Implications for the Spliceosome. RNA.

[7] Robart A.R., Chan R.T., Peters J.K., Rajashankar K.R., Toor N. (2014). Crystal Structure of a Eukaryotic Group II Intron Lariat. Nature.

[8] Toor N., Rajashankar K., Keating K.S., Pyle A.M. (2008). Structural Basis for Exon Recognition by a Group II Intron. Nat. Struct. Mol. Biol..

[9] Novikova O., Belfort M. (2017). Mobile Group II Introns as Ancestral Eukaryotic Elements. Trends Genet..

[10] Mukhopadhyay J., Hausner G. (2021). Organellar introns in fungi, algae, and plants. Cells.

[11] Small I., Melonek J., Bohne A.-V., Nickelsen J., Schmitz-Linneweber C. (2023). Plant Organellar RNA Maturation. Plant Cell.

[12] Brown G.G., Colas des Francs-Small C., Ostersetzer-Biran O. (2014). Group II Intron Splicing Factors in Plant Mitochondria. Front. Plant Sci..

[13] Zoschke R., Nakamura M., Liere K., Sugiura M., Börner T., Schmitz-Linneweber C. (2010). An Organellar Maturase Associates with Multiple Group II Introns. Proc. Natl. Acad. Sci. USA.

[14] Zeng C., Jiao Q., Jia T., Hu X. (2022). Updated Progress on Group II Intron Splicing Factors in Plant Chloroplasts. Curr. Issues Mol. Biol..

[15] Liu F., Melton J.T., Lopez-Bautista J.M., Chen N. (2020). Multiple Intraspecific Variations of Mitochondrial Genomes in the Green-Tide Forming Alga, *Ulva compressa* Linnaeus (Ulvophyceae, Chlorophyta). Front. Mar. Sci..

[16] Belfort M., Bonocora R.P. (2014). Homing Endonucleases: From Genetic Anomalies to Programmable Genomic Clippers. Methods Mol. Biol..

[17] Qu G., Kaushal P.S., Wang J., Shigematsu H., Piazza C.L., Agrawal R.K., Belfort M., Wang H.-W. (2016). Structure of a Group II Intron in Complex with Its Reverse Transcriptase. Nat. Struct. Mol. Biol..

[18] Stoddard B.L. (2006). Homing endonuclease structure and function. Q. Rev. Biophys..

[19] Liu N., Dong X., Hu C., Zeng J., Wang J., Wang J., Wang H.W., Belfort M. (2020). Exon and Protein Positioning in a Pre-Catalytic Group II Intron RNP Primed for Splicing. Nucleic Acids Res..

[20] Monachello D., Lauraine M., Gillot S., Michel F., Costa M. (2021). A New RNA–DNA Interaction Required for Integration of Group II Intron Retrotransposons into DNA Targets. Nucleic Acids Res..

[21] García-Rodríguez F.M., Neira J.L., Marcia M., Molina-Sánchez M.D., Toro N. (2019). A Group II Intron-Encoded Protein Interacts with the Cellular Replicative Machinery through the β-Sliding Clamp. Nucleic Acids Res..

[22] Liu F., Melton J.T. (2021). Chloroplast Genomes of the Green-Tide Forming Alga *Ulva compressa*: Comparative Chloroplast Genomics in the Genus *Ulva* (Ulvophyceae, Chlorophyta). Front. Mar. Sci..

[23] Liu F., Melton J.T., Wang H., Wang J., Lopez-Bautista J.M. (2022). Understanding the Evolution of Mitochondrial Genomes in the Green Macroalgal Genus *Ulva* (Ulvophyceae, Chlorophyta). Front. Mar. Sci..

[24] Costa M., Walbott H., Monachello D., Westhof E., Michel F. (2016). Crystal Structures of a Group II Intron Lariat Primed for Reverse Splicing. Science.

[25] De Clerck O., Kao S.-M., Bogaert K.A., Blomme J., Foflonker F., Kwantes M., Vancaester E., Vanderstraeten L., Aydogdu E., Boesger J. (2018). Insights into the Evolution of Multicellularity from the Sea Lettuce Genome. Curr. Biol..

[26] Liu F., Wang H., Song W. (2022). Tandem Integration of Circular Plasmid Contributes Significantly to the Expanded Mitochondrial Genomes of the Green-Tide Forming Alga *Ulva meridionalis* (Ulvophyceae, Chlorophyta). Front. Plant Sci..

[27] Liu F., Chen N., Wang H., Li J., Wang J., Qu F. (2023). Novel Insights into Chloroplast Genome Evolution in the Green Macroalgal Genus *Ulva* (Ulvophyceae, Chlorophyta). Front. Plant Sci..

[28] Liu F., Jin S., Kim J.K., Wu X., Wang J. (2025). Occurrence Frequency, Molecular Evolution and Phylogenetic Utility of *Ulva*-Specific Chloroplast Group II Intron *infA*-62 Family. Front. Mar. Sci..

[29] Li C.-F., Costa M., Bassi G.S., Lai Y.-K., Michel F. (2011). Recurrent Insertion of 5′-Terminal Nucleotides and Loss of the Branchpoint Motif in Lineages of Group II Introns Inserted in Mitochondrial Preribosomal RNAs. RNA.

[30] Toor N., Hausner G., Zimmerly S. (2001). Coevolution of Group II Intron RNA Structures with Their Intron-Encoded Reverse Transcriptases. RNA.

[31] Pyle A.M., Fedorova O., Waldsich C. (2007). Folding of Group II Introns: A Model System for Large, Multidomain RNAs?. Trends Biochem. Sci..

[32] Fedorova O., Pyle A.M. (2005). Linking the Group II Intron Catalytic Domains: Tertiary Contacts and Structural Features of Domain 3. EMBO J..

[33] Haack D.B., Yan X., Zhang C., Hingey J., Lyumkis D., Baker T.S., Toor N. (2019). Cryo-EM Structures of a Group II Intron Reverse Splicing into DNA. Cell.

[34] Marcia M., Pyle A.M. (2012). Visualizing Group II Intron Catalysis through the Stages of Splicing. Cell.

[35] Simon D.M., Kelchner S.A., Zimmerly S. (2009). A Broadscale Phylogenetic Analysis of Group II Intron RNAs and Intron-Encoded Reverse Transcriptases. Mol. Biol. Evol..

[36] Mullineux S.-T., Costa M., Bassi G.S., Michel F., Hausner G. (2010). A Group II Intron Encodes a Functional LAGLIDADG Homing Endonuclease and Self-Splices under Moderate Temperature and Ionic Conditions. RNA.

[37] Toor N., Zimmerly S. (2002). Identification of a Family of Group II Introns Encoding LAGLIDADG ORFs Typical of Group I Introns. RNA.

[38] Pfeifer A., Martin B., Kämper J., Basse C.W. (2012). The Mitochondrial LSU rRNA Group II Intron of *Ustilago maydis* Encodes an Active Homing Endonuclease Likely Involved in Intron Mobility. PLoS ONE.

[39] Smith D.R. (2012). Updating Our View of Organelle Genome Nucleotide Landscape. Front. Genet..

[40] Stoddard B.L. (2014). Homing Endonucleases from Mobile Group I Introns: Discovery to Genome Engineering. Mob. DNA.

[41] Lang B.F., Beck N., Prince S., Sarrasin M., Rioux P., Burger G. (2023). Mitochondrial Genome Annotation with MFannot: A Critical Analysis of Gene Identification and Gene Model Prediction. Front. Plant Sci..

[42] Lang B.F., Laforest M.-J., Burger G. (2007). Mitochondrial Introns: A Critical View. Trends Genet..

[43] Lambert A., Fontaine J.-F., Legendre M., Leclerc F., Permal E., Major F., Putzer H., Delfour O., Michot B., Gautheret D. (2004). The ERPIN Server: An Interface to Profile-Based RNA Motif Identification. Nucleic Acids Res..

[44] Reuter J.S., Mathews D.H. (2010). RNAstructure: Software for RNA Secondary Structure Prediction and Analysis. BMC Bioinform..

[45] Markham N.R., Zuker M. (2008). UNAFold: Software for Nucleic Acid Folding and Hybridization. Methods Mol. Biol..

[46] Zuker M. (2003). Mfold Web Server for Nucleic Acid Folding and Hybridization Prediction. Nucleic Acids Res..

[47] Darty K., Denise A., Ponty Y. (2009). VARNA: Interactive Drawing and Editing of the RNA Secondary Structure. Bioinformatics.

[48] Camacho C., Coulouris G., Avagyan V., Ma N., Papadopoulos J., Bealer K., Madden T.L. (2009). BLAST+: Architecture and Applications. BMC Bioinform..

[49] Tamura K., Stecher G., Kumar S. (2021). MEGA11: Molecular Evolutionary Genetics Analysis Version 11. Mol. Biol. Evol..

[50] Crooks G.E., Hon G., Chandonia J.-M., Brenner S.E. (2004). WebLogo: A Sequence Logo Generator. Genome Res..

[51] Wong T.K.F., Ly-Trong N., Ren H., Banos H., Roger A.J., Susko E., Bielow C., De Maio N., Goldman N., Hahn M.W. (2025). IQ-TREE 3: Phylogenomic Inference Software using Complex Evolutionary Models. Life Sci..

[52] Kalyaanamoorthy S., Minh B.Q., Wong T.K.F., von Haeseler A., Jermiin L.S. (2017). ModelFinder: Fast Model Selection for Accurate Phylogenetic Estimates. Nat. Methods.

[53] Hoang D.T., Chernomor O., von Haeseler A., Minh B.Q., Vinh L.S. (2018). UFBoot2: Improving the Ultrafast Bootstrap Approximation. Mol. Biol. Evol..

[54] Guindon S., Dufayard J.-F., Lefort V., Anisimova M., Hordijk W., Gascuel O. (2010). New Algorithms and Methods to Estimate Maximum-Likelihood Phylogenies: Assessing the Performance of PhyML 3.0. Syst. Biol..

[55] Edgar R.C. (2004). MUSCLE: Multiple Sequence Alignment with High Accuracy and High Throughput. Nucleic Acids Res..

