# Supplementary material for: Structural Remodeling and Enzymatic Replacement Shape the Evolution of Organellar Group II Introns in Ulva
Source: Int J Mol Sci. 2026 Mar 12;27(6):2613. doi: 10.3390/ijms27062613 (PMC13026550; doi:10.3390/ijms27062613)
Supplement: Supplementary file 1 [file ijms-27-02613-s001.zip › Supplementary Figure S1. NT-Tree.pdf]

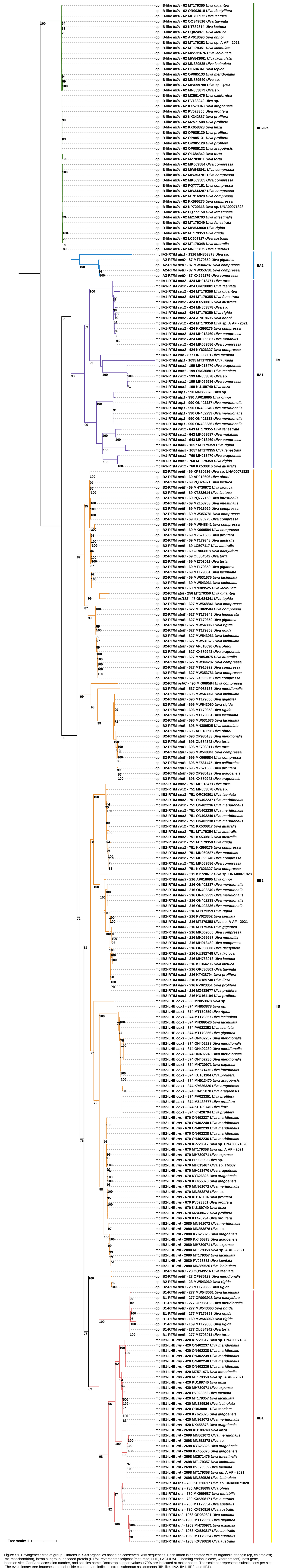

Figure S1. Phylogenetic tree of group II introns in *Ulva* organelles based on conserved RNA sequences. Each intron is annotated with its organelle of origin (cp, chloroplast; mt, mitochondrion), intron subgroup, encoded protein (RT/M, reverse transcriptase/maturase; LHE, LAGLIDADG homing endonuclease, wherepresent), host gene, insertion site, GenBank accession number, and species name. Bootstrap support values >70% are indicated at major nodes. The scale bar represents substitutions per site. The evolutionary tree branches and right-side colored bars indicate intron subgroup assignments (IIB-like, IIA2, IIA1, IIB2, and IIB1).
