# Supplementary material for: Structural Remodeling and Enzymatic Replacement Shape the Evolution of Organellar Group II Introns in Ulva
Source: Int J Mol Sci. 2026 Mar 12;27(6):2613. doi: 10.3390/ijms27062613 (PMC13026550; doi:10.3390/ijms27062613)
Supplement: Supplementary file 1 [file ijms-27-02613-s001.zip › Supplementary Figure S2. RTM-AA.pdf]

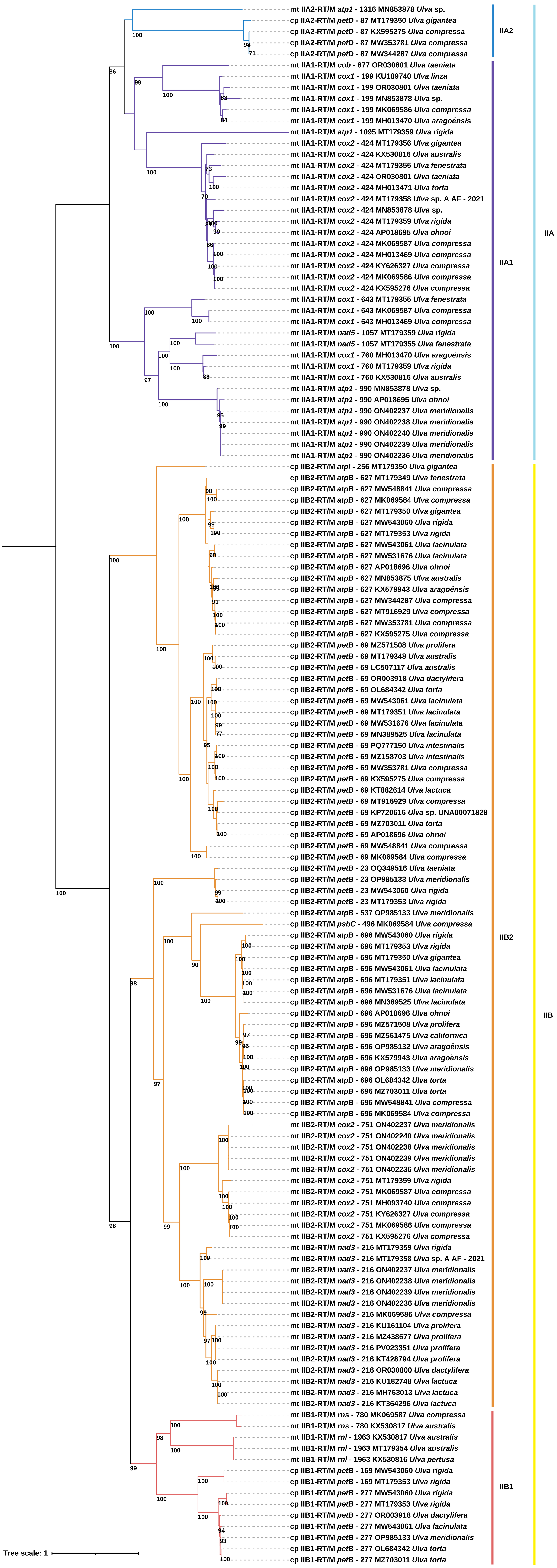

Figure S2. Phylogenetic tree based on conserved RT/M proteins encoded by group II introns in *Ulva* chloroplasts and mitochondria. Each branch represents an intron, annotated with its organelle source (cp, chloroplast; mt, mitochondrion), intron subgroup, host gene,intron insertion site, GenBank accession number, and species name. Bootstrap support values greater than 70% are shown at major nodes.The scale bar represents substitutions per site. The evolutionary tree branchesand right-side colored bars indicate intron subgroup assignments (IIA2, IIA1, IIB2, and IIB1).
