# Supplementary material for: Structural Remodeling and Enzymatic Replacement Shape the Evolution of Organellar Group II Introns in Ulva
Source: Int J Mol Sci. 2026 Mar 12;27(6):2613. doi: 10.3390/ijms27062613 (PMC13026550; doi:10.3390/ijms27062613)
Supplement: Supplementary file 1 [file ijms-27-02613-s001.zip › Supplementary Table S1. Ulva mitogenomes.pdf]

Table S1. The group II introns detected in mitochondrial genomes (mitogenomes) of *Ulva* species.

Genome	Species	GenBank accession number	Genome size (bp)	Genome GC (%)	Group II intron number
Mitogenome	*Ulva ohnoi*	AP018695	65,326	34.11	4
Mitogenome	*Ulva* sp. UNA00071828	KP720617	73,493	32.17	4
Mitogenome	*Ulva lactuca*	KT364296	61,614	32.49	1
Mitogenome	*Ulva prolifera*	KT428794	63,845	33.96	3
Mitogenome	*Ulva prolifera*	KU161104	61,962	33.86	3
Mitogenome	*Ulva lactuca*	KU182748	62,021	32.23	1
Mitogenome	*Ulva linza*	KU189740	70,858	34.61	6
Mitogenome	*Ulva aragoënsis*	KX455878	71,545	34.16	5
Mitogenome	*Ulva australis*	KX530816	69,333	35.86	5
Mitogenome	*Ulva australis*	KX530817	64,602	34.89	3
Mitogenome	*Ulva compressa*	KX595276	62,311	36.92	2
Mitogenome	*Ulva aragoënsis*	KY626326	71,527	34.16	5
Mitogenome	*Ulva compressa*	KY626327	62,477	36.97	2
Mitogenome	*Ulva* sp. TM637	MH013467	67,506	32.46	1
Mitogenome	*Ulva compressa*	MH013469	61,700	38.04	3
Mitogenome	*Ulva aragoënsis*	MH013470	63,526	34.69	4
Mitogenome	*Ulva torta*	MH013471	65,772	34.14	2
Mitogenome	*Ulva compressa*	MH093740	62,791	36.48	1
Mitogenome	*Ulva expansa*	MH730971	64,143	34.13	5
Mitogenome	*Ulva lactuca*	MH763013	>61,125	32.73	1
Mitogenome	*Ulva compressa*	MK069586	>66,587	37.73	4
Mitogenome	*Ulva compressa*	MK069587	>67,021	38.84	5
Mitogenome	*Ulva lacinulata*	MN389526	75908	32.72	4
Mitogenome	*Ulva* sp.	MN853878	>107,512	36.34	10
Mitogenome	*Ulva meridionalis*	MN861072	>62,887	34.2	4
Mitogenome	*Ulva australis*	MT179354	64,466	34.93	3
Mitogenome	*Ulva fenestrata*	MT179355	59,026	35.37	3
Mitogenome	*Ulva gigantea*	MT179356	66,743	32.96	4
Mitogenome	*Ulva lacinulata*	MT179357	79,723	32.56	4
Mitogenome	*Ulva* sp. A AF-2021	MT179358	88,318	33.39	6
Mitogenome	*Ulva rigida*	MT179359	88,416	36.42	7
Mitogenome	*Ulva prolifera*	MZ438677	63,843	33.96	3
Mitogenome	*Ulva intestinalis*	MZ571476	68,139	34.86	3
Mitogenome	*Ulva meridionalis*	ON402236	100,796	33.78	6
Mitogenome	*Ulva meridionalis*	ON402237	95,439	33.75	6
Mitogenome	*Ulva meridionalis*	ON402238	82,944	33.82	6
Mitogenome	*Ulva meridionalis*	ON402239	82,944	33.82	6
Mitogenome	*Ulva meridionalis*	ON402240	111,485	33.85	6
Mitogenome	*Ulva dactylifera*	OR030800	54174	32.84	1
Mitogenome	*Ulva taeniata*	OR030801	>73,098	34.35	7

Mitogenome	*Ulva* sp.	PP908992	87298	33.49	1
Mitogenome	*Ulva prolifera*	PV023351	63880	34.09	3
Mitogenome	*Ulva taeniata*	PV023352	79981	33.18	5
Mitogenome	*Ulva* sp. TM708	MH013468	55,814	33.22	0
	Number of mitochondrial group II introns				168
