# Supplementary material for: Structural Remodeling and Enzymatic Replacement Shape the Evolution of Organellar Group II Introns in Ulva
Source: Int J Mol Sci. 2026 Mar 12;27(6):2613. doi: 10.3390/ijms27062613 (PMC13026550; doi:10.3390/ijms27062613)
Supplement: Supplementary file 1 [file ijms-27-02613-s001.zip › Supplementary Table S2. Ulva plastomes.pdf]

Table S2. The group II introns detected in chloroplast genomes (plastomes) of *Ulva* species.

Genome	Species	GenBank accession number	Genome size (bp)	Genome GC (%)	Group II intron number
Plastome	*Ulva ohnoi*	AP018696	103,313	25.44	4
Plastome	*Ulva* sp. UNA00071828	KP720616	99,983	25.3	2
Plastome	*Ulva lactuca*	KT882614	96,005	24.87	2
Plastome	*Ulva linza*	KX058323	86,726	24.79	1
Plastome	*Ulva prolifera*	KX342867	93,066	24.78	1
Plastome	*Ulva aragoënsis*	KX579943	89,414	24.97	3
Plastome	*Ulva compressa*	KX595275	96,808	26.18	4
Plastome	*Ulva australis*	LC507117	102,899	25.33	2
Plastome	*Ulva lactuca*	MH730972	95,997	24.87	2
Plastome	*Ulva compressa*	MK069584	119,866	26.24	5
Plastome	*Ulva compressa*	MK069585	>89,164	26.25	1
Plastome	*Ulva lacinulata*	MN389525	103,523	25.4	3
Plastome	*Ulva australis*	MN853875	104,380	25.66	2
Plastome	*Ulva* sp.	MN853879	88,801	23.89	1
Plastome	*Ulva* sp.	MN889540	>88,653	23.91	1
Plastome	*Ulva australis*	MT179348	99,820	25.21	2
Plastome	*Ulva fenestrata*	MT179349	94,654	25.27	2
Plastome	*Ulva gigantea*	MT179350	117,606	25.73	6
Plastome	*Ulva lacinulata*	MT179351	103,444	25.4	3
Plastome	*Ulva* sp. A AF-2021	MT179352	96,673	24.57	1
Plastome	*Ulva rigida*	MT179353	118,206	26.12	6
Plastome	*Ulva compressa*	MT916929	94,226	25.8	3
Plastome	*Ulva compressa*	MW344287	91,189	25.86	3
Plastome	*Ulva compressa*	MW353781	96,824	26.17	4
Plastome	*Ulva lacinulata*	MW531676	110,889	25.63	4
Plastome	*Ulva rigida*	MW543060	117,995	26.13	6
Plastome	*Ulva lacinulata*	MW543061	107,242	25.82	5
Plastome	*Ulva compressa*	MW548841	114,291	26.23	4
Plastome	*Ulva* sp. Q253	MW699788	88,801	23.89	1
Plastome	*Ulva intestinalis*	MZ158703	99,041	24.97	2
Plastome	*Ulva californica*	MZ561475	92,126	24.71	2
Plastome	*Ulva prolifera*	MZ571508	99,724	25.28	3
Plastome	*Ulva torta*	MZ703011	105,423	25.24	4
Plastome	*Ulva tepida*	OL684341	94,449	24.49	2
Plastome	*Ulva torta*	OL684342	112,034	24.89	4
Plastome	*Ulva prolifera*	OP985129	93,066	24.78	1
Plastome	*Ulva prolifera*	OP985130	93,066	24.78	1
Plastome	*Ulva prolifera*	OP985131	93,072	24.78	1
Plastome	*Ulva aragoënsis*	OP985132	87,172	24.68	2
Plastome	*Ulva meridionalis*	OP985133	122,172	24.86	5

Plastome	*Ulva taeniata*	OQ349516	100,923	25.7	2
Plastome	*Ulva dactylifera*	OR003918	93,205	25.41	3
Plastome	*Ulva intestinalis*	PQ777150	94,778	24.96	2
Plastome	*Ulva compressa*	PQ777151	89,239	25.33	1
Plastome	*Ulva lactuca*	PQ824971	96,008	24.86	2
Plastome	*Ulva prolifera*	PV023350	93,152	24.8	1
Plastome	*Ulva* sp.	PV138240	92,301	24.42	1
	Number of chloroplast group II introns				123
