# Supplementary material for: Structural Remodeling and Enzymatic Replacement Shape the Evolution of Organellar Group II Introns in Ulva
Source: Int J Mol Sci. 2026 Mar 12;27(6):2613. doi: 10.3390/ijms27062613 (PMC13026550; doi:10.3390/ijms27062613)
Supplement: Supplementary file 1 [file ijms-27-02613-s001.zip › Supplementary Table S3. Intron distribution and type.pdf]

Table S3. The type and number of group II introns detected in organellar genomes (mitogenomes or plastomes) of *Ulva* species.

Host genome	Intron type	Intron name	Intron number	GenBank accession number	Species name
mitogenome	RT/M	*atp1*-1095	1	MT179359	*Ulva rigida*
mitogenome	RT/M	*atp1*-1316	1	MN853878	*Ulva* sp.
mitogenome	RT/M	*atp1*-990	7	AP018695	*Ulva ohnoi*
				ON402240	*Ulva meridionalis*
				ON402236	*Ulva meridionalis*
				ON402237	*Ulva meridionalis*
				ON402238	*Ulva meridionalis*
				ON402239	*Ulva meridionalis*
				MN853878	*Ulva* sp.
mitogenome	RT/M	*cob*-877	1	OR030801	*Ulva taeniata*
mitogenome	RT/M	*cox1*-199	5	KU189740	*Ulva linza*
				MH013470	*Ulva aragoënsis*
				MK069586	*Ulva compressa*
				MN853878	*Ulva* sp.
				OR030801	*Ulva taeniata*
mitogenome	RT/M	*cox1*-643	3	MH013469	*Ulva compressa*
				MK069587	*Ulva compressa*
				MT179355	*Ulva fenestrata*
mitogenome	RT/M	*cox1*-760	3	KX530816	*Ulva australis*
				MH013470	*Ulva aragoënsis*
				MT179359	*Ulva rigida*
mitogenome	RT/M	*cox2*-424	14	AP018695	*Ulva ohnoi*
				KX530816	*Ulva australis*
				KX595276	*Ulva compressa*
				KY626327	*Ulva compressa*
				MH013469	*Ulva compressa*
				MH013471	*Ulva torta*
				MK069586	*Ulva compressa*
				MK069587	*Ulva compressa*
				MN853878	*Ulva* sp.
				MT179355	*Ulva fenestrata*
				MT179356	*Ulva gigantea*
				MT179358	*Ulva* sp. A AF-2021
				MT179359	*Ulva rigida*
				OR030801	*Ulva taeniata*
mitogenome	RT/M	*nad5*-1057	2	MT179355	*Ulva fenestrata*
				MT179359	*Ulva rigida*
mitogenome	RT/M	*cox2*-751	17	KX530816	*Ulva australis*

				KX530817	*Ulva australis*
				KX595276	*Ulva compressa*
				KY626327	*Ulva compressa*
				ON402240	*Ulva meridionalis*
				ON402236	*Ulva meridionalis*
				ON402237	*Ulva meridionalis*
				ON402238	*Ulva meridionalis*
				ON402239	*Ulva meridionalis*
				MH013471	*Ulva torta*
				MH093740	*Ulva compressa*
				MK069586	*Ulva compressa*
				MK069587	*Ulva compressa*
				MN853878	*Ulva* sp.
				MT179354	*Ulva australis*
				MT179359	*Ulva rigida*
				OR030801	*Ulva taeniata*
mitogenome	RT/M	*nad3*-215	1	KP720617	*Ulva* sp. UNA00071828
mitogenome	RT/M	*nad3*-216	23	AP018695	*Ulva ohnoi*
				KT364296	*Ulva lactuca*
				KT428794	*Ulva prolifera*
				KU161104	*Ulva prolifera*
				KU182748	*Ulva lactuca*
				KU189740	*Ulva linza*
				ON402240	*Ulva meridionalis*
				ON402236	*Ulva meridionalis*
				ON402237	*Ulva meridionalis*
				ON402238	*Ulva meridionalis*
				ON402239	*Ulva meridionalis*
				MH013469	*Ulva compressa*
				MH763013	*Ulva lactuca*
				MK069586	*Ulva compressa*
				MK069587	*Ulva compressa*
				MT179356	*Ulva gigantea*
				MT179358	*Ulva* sp. A AF-2021
				MT179359	*Ulva rigida*
				MZ438677	*Ulva prolifera*
				OR030800	*Ulva dactylifera*
				OR030801	*Ulva taeniata*
				PV023351	*Ulva prolifera*
				PV023352	*Ulva taeniata*
mitogenome	RT/M	*rnl*-1963	6	KX530816	*Ulva pertusa*

				KX530817	*Ulva australis*
				MH730971	*Ulva expansa*
				MT179354	*Ulva australis*
				MT179356	*Ulva gigantea*
				OR030801	*Ulva taeniata*
mitogenome	RT/M	*rns*-780	6	AP018695	*Ulva ohnoi*
				KP720617	*Ulva* sp. UNA00071828
				KX530816	*Ulva pertusa*
				KX530817	*Ulva australis*
				MK069587	*Ulva compressa*
				MT179354	*Ulva australis*
mitogenome	LHE	*cox1*-686	1	MN853878	*Ulva* sp.
mitogenome	LHE	*cox1*-874	21	KT428794	*Ulva prolifera*
				KU161104	*Ulva prolifera*
				KU189740	*Ulva linza*
				KX455878	*Ulva aragoënsis*
				KY626326	*Ulva aragoënsis*
				MH013470	*Ulva aragoënsis*
				MH730971	*Ulva expansa*
				MN389526	*Ulva lacinulata*
				MN853878	*Ulva* sp.
				MT179356	*Ulva gigantea*
				MT179357	*Ulva lacinulata*
				MT179359	*Ulva rigida*
				MZ438677	*Ulva prolifera*
				MZ571476	*Ulva intestinalis*
				ON402236	*Ulva meridionalis*
				ON402237	*Ulva meridionalis*
				ON402238	*Ulva meridionalis*
				ON402239	*Ulva meridionalis*
				ON402240	*Ulva meridionalis*
				PV023351	*Ulva prolifera*
				PV023352	*Ulva taeniata*
mitogenome	LHE	*rnl*-2080	9	KX455878	*Ulva aragoënsis*
				KY626326	*Ulva aragoënsis*
				MH730971	*Ulva expansa*
				MN389526	*Ulva lacinulata*
				MN853878	*Ulva* sp.
				MN861072	*Ulva meridionalis*
				MT179357	*Ulva lacinulata*
				MT179358	*Ulva* sp. A AF-2021

				PV023352	*Ulva taeniata*
mitogenome	LHE	*rnl*-2698	10	KU189740	*Ulva linza*
				KX455878	*Ulva aragoënsis*
				KY626326	*Ulva aragoënsis*
				MN389526	*Ulva lacinulata*
				MN853878	*Ulva* sp.
				MN861072	*Ulva meridionalis*
				MT179357	*Ulva lacinulata*
				MT179358	*Ulva* sp. A AF-2021
				MZ571476	*Ulva intestinalis*
				PV023352	*Ulva taeniata*
mitogenome	LHE	*rns*-420	17	KP720617	*Ulva* sp. UNA00071828
				KU189740	*Ulva linza*
				KX455878	*Ulva aragoënsis*
				KY626326	*Ulva aragoënsis*
				MH730971	*Ulva expansa*
				MN389526	*Ulva lacinulata*
				MN861072	*Ulva meridionalis*
				MT179357	*Ulva lacinulata*
				MT179358	*Ulva* sp. A AF-2021
				MZ571476	*Ulva intestinalis*
				ON402236	*Ulva meridionalis*
				ON402237	*Ulva meridionalis*
				ON402238	*Ulva meridionalis*
				ON402239	*Ulva meridionalis*
				ON402240	*Ulva meridionalis*
				OR030801	*Ulva taeniata*
				PV023352	*Ulva taeniata*
mitogenome	LHE	*rns*-670	20	KP720617	*Ulva* sp.
				KT428794	*Ulva prolifera*
				KU161104	*Ulva prolifera*
				KU189740	*Ulva linza*
				KX455878	*Ulva aragoënsis*
				KY626326	*Ulva aragoënsis*
				MH013467	*Ulva* sp. TM637
				MH013470	*Ulva aragoënsis*
				MH730971	*Ulva expansa*
				MN853878	*Ulva* sp.
				MN861072	*Ulva meridionalis*
				MT179358	*Ulva* sp. A AF-2021
				MZ438677	*Ulva prolifera*

				ON402236	*Ulva meridionalis*
				ON402237	*Ulva meridionalis*
				ON402238	*Ulva meridionalis*
				ON402239	*Ulva meridionalis*
				ON402240	*Ulva meridionalis*
				PP908992	*Ulva* sp.
				PV023351	*Ulva prolifera*
plastome	RT/M	*petD*-87	4	KX595275	*Ulva compressa*
				MT179350	*Ulva gigantea*
				MW344287	*Ulva compressa*
				MW353781	*Ulva compressa*
plastome	RT/M	*atpB*-537	1	OP985133	*Ulva meridionalis*
plastome	RT/M	*atpB*-627	15	AP018696	*Ulva ohnoi*
				KX579943	*Ulva aragoënsis*
				KX595275	*Ulva compressa*
				MK069584	*Ulva compressa*
				MN853875	*Ulva australis*
				MT179349	*Ulva fenestrata*
				MT179350	*Ulva gigantea*
				MT179353	*Ulva rigida*
				MT916929	*Ulva compressa*
				MW344287	*Ulva compressa*
				MW353781	*Ulva compressa*
				MW531676	*Ulva lacinulata*
				MW543060	*Ulva rigida*
				MW543061	*Ulva lacinulata*
				MW548841	*Ulva compressa*
plastome	RT/M	*atpB*-696	17	AP018696	*Ulva ohnoi*
				KX579943	*Ulva aragoënsis*
				OP985132	*Ulva aragoënsis*
				OP985133	*Ulva meridionalis*
				MK069584	*Ulva compressa*
				MN389525	*Ulva lacinulata*
				MT179350	*Ulva gigantea*
				MT179351	*Ulva lacinulata*
				MT179353	*Ulva rigida*
				MW531676	*Ulva lacinulata*
				MW543060	*Ulva rigida*
				MW543061	*Ulva lacinulata*
				MW548841	*Ulva compressa*
				MZ561475	*Ulva californica*

				MZ571508	*Ulva prolifera*
				MZ703011	*Ulva torta*
				OL684342	*Ulva torta*
plastome	RT/M	*atpI*-256	1	MT179350	*Ulva gigantea*
plastome	RT/M	*petB*-169	2	MT179353	*Ulva rigida*
				MW543060	*Ulva rigida*
plastome	RT/M	*petB*-23	4	OP985133	*Ulva meridionalis*
				MT179353	*Ulva rigida*
				MW543060	*Ulva rigida*
				OQ349516	*Ulva taeniata*
plastome	RT/M	*petB*-277	7	MT179353	*Ulva rigida*
				MW543060	*Ulva rigida*
				MW543061	*Ulva lacinulata*
				MZ703011	*Ulva torta*
				OL684342	*Ulva torta*
				OP985133	*Ulva meridionalis*
				OR003918	*Ulva dactylifera*
plastome	RT/M	*petB*-69	23	AP018696	*Ulva ohnoi*
				KP720616	*Ulva* sp. UNA00071828
				KT882614	*Ulva lactuca*
				KX595275	*Ulva compressa*
				LC507117	*Ulva australis*
				MH730972	*Ulva lactuca*
				MK069584	*Ulva compressa*
				MN389525	*Ulva lacinulata*
				MT179348	*Ulva australis*
				MT179350	*Ulva gigantea*
				MT179351	*Ulva lacinulata*
				MT916929	*Ulva compressa*
				MW353781	*Ulva compressa*
				MW531676	*Ulva lacinulata*
				MW543061	*Ulva lacinulata*
				MW548841	*Ulva compressa*
				MZ158703	*Ulva intestinalis*
				MZ571508	*Ulva prolifera*
				MZ703011	*Ulva torta*
				OL684342	*Ulva torta*
				OR003918	*Ulva dactylifera*
				PQ777150	*Ulva intestinalis*
				PQ824971	*Ulva lactuca*
plastome	RT/M	*psbC*-496	1	MK069584	*Ulva compressa*

plastome	RT/M	*orf185*-47	1	OL684341	*Ulva tepida*
plastome	IIB-like	*infA*-62	47	KP720616	*Ulva* sp. UNA00071828
				AP018696	*Ulva ohnoi*
				KT882614	*Ulva lactuca*
				KX058323	*Ulva linza*
				KX342867	*Ulva prolifera*
				KX579943	*Ulva aragoënsis*
				KX595275	*Ulva compressa*
				LC507117	*Ulva australis*
				MH730972	*Ulva lactuca*
				MK069584	*Ulva compressa*
				MK069585	*Ulva compressa*
				MN389525	*Ulva lacinulata*
				MN853875	*Ulva australis*
				MN853879	*Ulva* sp.
				MN889540	*Ulva* sp.
				MT179348	*Ulva australis*
				MT179349	*Ulva fenestrata*
				MT179350	*Ulva gigantea*
				MT179351	*Ulva lacinulata*
				MT179352	*Ulva* sp. A AF-2021
				MT179353	*Ulva rigida*
				MT916929	*Ulva compressa*
				MW344287	*Ulva compressa*
				MW353781	*Ulva compressa*
				MW531676	*Ulva lacinulata*
				MW543060	*Ulva rigida*
				MW543061	*Ulva lacinulata*
				MW548841	*Ulva compressa*
				MW699788	*Ulva* sp. Q253
				MZ158703	*Ulva intestinalis*
				MZ561475	*Ulva californica*
				MZ571508	*Ulva prolifera*
				MZ703011	*Ulva torta*
				OL684341	*Ulva tepida*
				OL684342	*Ulva torta*
				OP985129	*Ulva prolifera*
				OP985130	*Ulva prolifera*
				OP985131	*Ulva prolifera*
				OP985132	*Ulva aragoënsis*
				OP985133	*Ulva meridionalis*

	OQ349516	*Ulva taeniata*
	OR003918	*Ulva dactylifera*
	PQ777150	*Ulva intestinalis*
	PQ777151	*Ulva compressa*
	PQ824971	*Ulva lactuca*
	PV023350	*Ulva prolifera*
	PV138240	*Ulva* sp.
