# Supplementary material for: Structural Remodeling and Enzymatic Replacement Shape the Evolution of Organellar Group II Introns in Ulva
Source: Int J Mol Sci. 2026 Mar 12;27(6):2613. doi: 10.3390/ijms27062613 (PMC13026550; doi:10.3390/ijms27062613)
Supplement: Supplementary file 1 [file ijms-27-02613-s001.zip › Supplementary Table S4. Size and GC of intron lineages.pdf]

Table S4. The average size and GC content of 32 group II intron families in *Ulva* organellar genomes, as well as the differences in GC content between intron families and host genomes.

Host genomes	Class	Intron family	Intron number (n)	Avg. size of family (SD, nt)	Avg. size of introns (SD, nt)	Avg. GC of family (SD, %)	Avg. GC of introns (SD, %)	Avg. GC of host genomes (SD, %)	Significance in GC (*P*)
Mitogenomes (mt)	RT/M	*atp1*-1095	1	2547	2449 (260.50)	43.46	45.56 (2.45)	36.42	-
		atp1-1316	1	3081		45.70		36.34	-
		atp1-990	7	2584 (2.27)		49.04 (0.42)		33.80 (0.04)	<0.001
		cob-877	1	2331		45.99		34.35	-
		cox1-199	5	2493 (22.96)		45.37 (1.02)		34.65 (0.06)	<0.001
		cox1-643	3	2511 (5.20)		45.60 (0.44)		36.71 (1.89)	0.003
		cox1-760	3	2641 (45.64)		45.67 (1.83)		35.66 (0.88)	<0.001
		cox2-424	14	2513 (66.32)		46.22 (1.73)		35.42 (1.71)	<0.001
		nad5-1057	2	2640 (12.73)		45.72 (0.16)		35.90 (0.74)	0.003
		cox2-751	17	2430 (113.00)		4.60 (1.18)		35.05 (1.30)	<0.001
		nad3-215	1	2727		40.15		32.17	-
		nad3-216	23	2455 (187.83)		43.47 (1.64)		33.96 (1.33)	<0.001
		rnl-1963	6	2051 (474.70)		41.87 (0.83)		34.55 (1.08)	<0.001
		rns-780	6	2193 (571.08)		47.13 (0.91)		34.39 (1.39)	<0.001
	LHE	*cox1*-686	1	789	1364 (262.70)	40.81	35.55 (1.92)	36.34	-
		cox1-874	21	1530 (133.02)		34.19 (1.23)		33.97 (0.83)	0.503
		rnl-2080	9	1562 (38.21)		37.86 (0.28)		33.47 (0.69)	<0.001
		rnl-2698	10	1375 (21.72)		36.67 (0.56)		33.71 (0.86)	<0.001
		rns-420	17	1186 (297.27)		35.55 (2.34)		33.66 (0.75)	0.006
		rns-670	20	1276 (287.64)		35.12 (1.26)		33.79 (0.63)	<0.001

		petD-87	4	2428 (11.32)		36.23 (0.03)		25.99 (0.23)	<0.001
		atpB-537	1	2355		33.38		24.86	-
		atpB-627	15	2225 (13.49)		36.79 (0.93)		25.82 (0.38)	<0.001
		atpB-696	17	2372 (12.18)		36.52 (0.52)		25.46 (0.53)	<0.001
		atpI-256	1	2252		36.23		25.73	-
Plastomes (cp)	RT/M	*petB*-23	4	2305 (21.35)	2281 (201.82)	34.69 (0.43)	36.09 (1.18)	25.70 (0.60)	<0.001
		petB-69	23	2209 (85.77)		35.42 (0.97)		25.44 (0.46)	<0.001
		petB-169	2	2459 (0.00)		34.85 (0.00)		26.13 (0.01)	<0.001
		petB-277	7	2450 (13.89)		36.33 (0.38)		25.50 (0.54)	<0.001
		psbC-496	1	2441		36.71		26.24	-
		orf185-47	1	800		41.12		24.49	-
	IIB-like	*infA*-62	47	615 (68.89)	615 (68.89)	22.73 (1.19)	22.73 (1.19)	25.21 (0.59)	<0.001
