# Supplementary material for: Structural Remodeling and Enzymatic Replacement Shape the Evolution of Organellar Group II Introns in Ulva
Source: Int J Mol Sci. 2026 Mar 12;27(6):2613. doi: 10.3390/ijms27062613 (PMC13026550; doi:10.3390/ijms27062613)
Supplement: Supplementary file 1 [file ijms-27-02613-s001.zip › Supplementary Table S5. Size and GC of domains.pdf]

Table S5. The size and GC content of 32 group II intron families and their domains (DI to DVI, and DIIIa) in secondary structures detected in organellar genomes of *Ulva* species.

Intron name	GenBank accession number	Species	Intron		DI		DII		DIII		DIIIa		DIV		DV		DVI	
			Size (nt)	GC (%)	Size (nt)	GC (%)	Size (nt)	GC (%)	Size (nt)	GC (%)	Size (nt)	GC (%)	Size (nt)	GC (%)	Size (nt)	GC (%)	Size (nt)	GC (%)
atpB-537	OP985133	*Ulva meridionalis*	2355	33.38	375	33.87	54	29.63	58	37.93	0	0.00	1764	32.77	34	44.12	35	40.00
atpB-627	KX579943	*Ulva aragoënsis*	2242	35.95	438	34.02	75	42.67	18	27.78	0	0.00	1612	35.48	34	52.94	34	47.06
atpB-627	MN853875	*Ulva australis*	2235	35.70	447	34.68	70	41.43	18	27.78	0	0.00	1601	35.23	34	52.94	34	41.18
atpB-627	KX595275	*Ulva compressa*	2224	36.38	437	36.38	58	48.28	18	27.78	0	0.00	1612	35.48	34	52.94	34	38.24
atpB-627	MK069584	*Ulva compressa*	2232	36.25	439	33.03	62	46.77	18	38.89	0	0.00	1614	35.87	34	52.94	34	50.00
atpB-627	MT916929	*Ulva compressa*	2224	36.38	437	36.38	58	48.28	18	27.78	0	0.00	1612	35.48	34	52.94	34	38.24
atpB-627	MW344287	*Ulva compressa*	2223	36.62	436	36.47	58	48.28	18	27.78	0	0.00	1612	35.79	34	52.94	34	38.24
atpB-627	MW353781	*Ulva compressa*	2224	36.38	437	36.38	58	48.28	18	27.78	0	0.00	1612	35.48	34	52.94	34	38.24
atpB-627	MW548841	*Ulva compressa*	2232	36.11	439	32.80	62	46.77	18	38.89	0	0.00	1614	35.75	34	52.94	34	50.00
atpB-627	MT179349	*Ulva fenestrata*	2250	38.76	442	35.75	57	49.12	18	27.78	0	0.00	1634	38.56	34	52.94	34	50.00
atpB-627	MT179350	*Ulva gigantea*	2237	38.13	433	37.88	66	50.00	18	27.78	0	0.00	1621	37.08	34	52.94	34	52.94
atpB-627	MW531676	*Ulva lacinulata*	2205	36.37	426	37.79	59	40.68	18	27.78	0	0.00	1603	35.12	34	52.94	34	52.94
atpB-627	MW543061	*Ulva lacinulata*	2203	36.40	426	38.03	57	40.35	18	27.78	0	0.00	1603	35.12	34	52.94	34	52.94
atpB-627	AP018696	*Ulva ohnoi*	2222	36.41	440	35.23	59	42.37	18	22.22	0	0.00	1606	36.05	34	52.94	34	41.18
atpB-627	MT179353	*Ulva rigida*	2211	38.04	433	36.49	57	47.37	18	27.78	0	0.00	1603	37.49	34	52.94	34	52.94
atpB-627	MW543060	*Ulva rigida*	2211	37.95	433	36.49	57	47.37	18	27.78	0	0.00	1603	37.37	34	52.94	34	52.94
atpB-696	KX579943	*Ulva aragoënsis*	2371	35.93	410	33.17	50	50.00	58	41.38	0	0.00	1760	35.68	34	52.94	34	26.47
atpB-696	OP985132	*Ulva aragoënsis*	2371	35.93	410	33.17	50	50.00	58	41.38	0	0.00	1760	35.68	34	52.94	34	26.47
atpB-696	MZ561475	*Ulva californica*	2375	36.08	414	33.57	50	52.00	58	41.38	0	0.00	1760	35.74	34	52.94	34	26.47
atpB-696	MK069584	*Ulva compressa*	2369	36.18	413	32.20	50	54.00	58	37.93	0	0.00	1755	36.18	34	52.94	34	29.41
atpB-696	MW548841	*Ulva compressa*	2373	36.03	412	32.28	50	54.00	58	37.93	0	0.00	1760	35.97	34	52.94	34	29.41

Intron name	GenBank accession number	Species	Intron		DI		DII		DIII		DIIIa		DIV		DV		DVI	
			Size (nt)	GC (%)	Size (nt)	GC (%)	Size (nt)	GC (%)	Size (nt)	GC (%)	Size (nt)	GC (%)	Size (nt)	GC (%)	Size (nt)	GC (%)	Size (nt)	GC (%)
atpB-696	MT179350	*Ulva gigantea*	2387	36.95	395	36.96	52	48.08	64	35.94	0	0.00	1783	36.23	34	52.94	34	35.29
atpB-696	MN389525	*Ulva lacinulata*	2364	36.89	396	37.12	52	48.08	58	39.66	0	0.00	1765	35.98	34	52.94	34	35.29
atpB-696	MT179351	*Ulva lacinulata*	2364	36.89	396	37.12	52	48.08	58	39.66	0	0.00	1765	35.98	34	52.94	34	35.29
atpB-696	MW531676	*Ulva lacinulata*	2364	36.89	396	37.12	52	48.08	58	39.66	0	0.00	1765	35.98	34	52.94	34	35.29
atpB-696	MW543061	*Ulva lacinulata*	2364	36.84	396	37.12	52	48.08	58	37.93	0	0.00	1765	35.98	34	52.94	34	35.29
atpB-696	OP985133	*Ulva meridionalis*	2376	37.29	416	32.93	50	52.00	58	41.38	0	0.00	1759	37.52	34	52.94	34	29.41
atpB-696	AP018696	*Ulva ohnoi*	2348	36.58	375	36.53	53	35.85	63	36.51	0	0.00	1764	36.34	34	52.94	34	26.47
atpB-696	MZ571508	*Ulva prolifera*	2400	35.46	439	31.44	50	50.00	58	41.38	0	0.00	1760	35.51	34	52.94	34	26.47
atpB-696	MT179353	*Ulva rigida*	2394	37.18	391	36.83	57	42.11	82	48.78	0	0.00	1771	36.19	34	52.94	34	35.29
atpB-696	MW543060	*Ulva rigida*	2370	36.96	391	37.08	57	42.11	58	43.10	0	0.00	1771	36.19	34	52.94	34	35.29
atpB-696	MZ703011	*Ulva torta*	2370	36.41	409	33.50	50	54.00	58	37.93	0	0.00	1760	36.19	34	52.94	34	29.41
atpB-696	OL684342	*Ulva torta*	2370	36.41	409	33.50	50	54.00	58	37.93	0	0.00	1760	36.19	34	52.94	34	29.41
atpI-256	MT179350	*Ulva gigantea*	2252	36.23	490	38.78	58	39.66	12	66.67	0	0.00	1587	34.53	34	44.12	34	50.00
infA-62	KX579943	*Ulva aragoënsis*	650	24.31	446	24.66	27	7.41	50	24.00	0	0.00	24	0.00	34	41.18	43	23.26
infA-62	OP985132	*Ulva aragoënsis*	650	24.31	446	24.66	27	7.41	50	24.00	0	0.00	24	0.00	34	41.18	43	23.26
infA-62	LC507117	*Ulva australis*	561	22.99	348	22.13	31	6.45	50	24.00	0	0.00	26	11.54	34	41.18	46	21.74
infA-62	MN853875	*Ulva australis*	561	22.99	348	22.13	31	6.45	50	24.00	0	0.00	26	11.54	34	41.18	46	21.74
infA-62	MT179348	*Ulva australis*	561	22.99	348	22.13	31	6.45	50	24.00	0	0.00	26	11.54	34	41.18	46	21.74
infA-62	MZ561475	*Ulva californica*	664	24.25	460	24.35	27	11.11	50	24.00	0	0.00	24	0.00	34	41.18	43	23.26
infA-62	KX595275	*Ulva compressa*	761	25.23	550	26.55	27	0.00	50	24.00	0	0.00	24	0.00	34	41.18	50	20.00
infA-62	MK069584	*Ulva compressa*	739	23.14	528	23.67	27	0.00	50	24.00	0	0.00	24	0.00	34	41.18	50	20.00
infA-62	MK069585	*Ulva compressa*	761	25.23	550	26.55	27	0.00	50	24.00	0	0.00	24	0.00	34	41.18	50	20.00
infA-62	MT916929	*Ulva compressa*	761	25.23	550	26.55	27	0.00	50	24.00	0	0.00	24	0.00	34	41.18	50	20.00

Intron name	GenBank accession number	Species	Intron		DI		DII		DIII		DIIIa		DIV		DV		DVI	
			Size (nt)	GC (%)	Size (nt)	GC (%)	Size (nt)	GC (%)	Size (nt)	GC (%)	Size (nt)	GC (%)	Size (nt)	GC (%)	Size (nt)	GC (%)	Size (nt)	GC (%)
infA-62	MW344287	*Ulva compressa*	728	23.76	517	24.56	27	0.00	50	24.00	0	0.00	24	0.00	34	41.18	50	20.00
infA-62	MW353781	*Ulva compressa*	761	25.23	550	26.55	27	0.00	50	24.00	0	0.00	24	0.00	34	41.18	50	20.00
infA-62	MW548841	*Ulva compressa*	739	23.00	528	23.48	27	0.00	50	24.00	0	0.00	24	0.00	34	41.18	50	20.00
infA-62	PQ777151	*Ulva compressa*	783	25.54	572	26.92	27	0.00	50	24.00	0	0.00	24	0.00	34	41.18	50	20.00
infA-62	OR003918	*Ulva dactylifera*	573	22.86	352	22.16	27	11.11	50	28.00	0	0.00	24	4.17	34	41.18	60	16.67
infA-62	MT179349	*Ulva fenestrata*	556	22.30	351	21.37	25	4.00	50	24.00	0	0.00	25	0.00	34	41.18	45	24.44
infA-62	MT179350	*Ulva gigantea*	563	22.56	356	21.63	27	7.41	50	28.00	0	0.00	24	0.00	34	41.18	46	21.74
infA-62	MZ158703	*Ulva intestinalis*	596	22.32	389	22.37	27	0.00	50	24.00	0	0.00	24	0.00	34	41.18	46	21.74
infA-62	PQ777150	*Ulva intestinalis*	596	22.32	389	22.37	27	0.00	50	24.00	0	0.00	24	0.00	34	41.18	46	21.74
infA-62	MN389525	*Ulva lacinulata*	571	21.72	357	21.01	27	7.41	50	26.00	0	0.00	24	0.00	34	41.18	53	18.87
infA-62	MT179351	*Ulva lacinulata*	571	21.72	357	21.01	27	7.41	50	26.00	0	0.00	24	0.00	34	41.18	53	18.87
infA-62	MW531676	*Ulva lacinulata*	571	21.72	357	21.01	27	7.41	50	26.00	0	0.00	24	0.00	34	41.18	53	18.87
infA-62	MW543061	*Ulva lacinulata*	571	21.72	357	21.01	27	7.41	50	26.00	0	0.00	24	0.00	34	41.18	53	18.87
infA-62	KT882614	*Ulva lactuca*	580	23.10	373	22.52	27	11.11	50	26.00	0	0.00	24	0.00	34	41.18	46	21.74
infA-62	MH730972	*Ulva lactuca*	580	23.10	373	22.52	27	11.11	50	26.00	0	0.00	24	0.00	34	41.18	46	21.74
infA-62	PQ824971	*Ulva lactuca*	580	23.10	373	22.52	27	11.11	50	26.00	0	0.00	24	0.00	34	41.18	46	21.74
infA-62	KX058323	*Ulva linza*	611	22.59	392	20.66	32	15.62	50	24.00	0	0.00	32	18.75	34	41.18	45	22.22
infA-62	OP985133	*Ulva meridionalis*	573	21.29	358	20.39	27	7.41	50	24.00	0	0.00	24	0.00	34	41.18	54	20.37
infA-62	AP018696	*Ulva ohnoi*	575	23.13	368	22.55	27	11.11	50	26.00	0	0.00	24	0.00	34	41.18	46	21.74
infA-62	KX342867	*Ulva prolifera*	616	22.56	397	20.65	32	15.62	50	24.00	0	0.00	32	18.75	34	41.18	45	22.22
infA-62	MZ571508	*Ulva prolifera*	616	22.56	397	20.65	32	15.62	50	24.00	0	0.00	32	18.75	34	41.18	45	22.22
infA-62	OP985129	*Ulva prolifera*	616	22.56	397	20.65	32	15.62	50	24.00	0	0.00	32	18.75	34	41.18	45	22.22
infA-62	OP985130	*Ulva prolifera*	616	22.56	397	20.65	32	15.62	50	24.00	0	0.00	32	18.75	34	41.18	45	22.22

Intron name	GenBank accession number	Species	Intron		DI		DII		DIII		DIIIa		DIV		DV		DVI	
			Size (nt)	GC (%)	Size (nt)	GC (%)	Size (nt)	GC (%)	Size (nt)	GC (%)	Size (nt)	GC (%)	Size (nt)	GC (%)	Size (nt)	GC (%)	Size (nt)	GC (%)
infA-62	OP985131	*Ulva prolifera*	616	22.56	397	20.65	32	15.62	50	24.00	0	0.00	32	18.75	34	41.18	45	22.22
infA-62	PV023350	*Ulva prolifera*	616	22.56	397	20.65	32	15.62	50	24.00	0	0.00	32	18.75	34	41.18	45	22.22
infA-62	MT179353	*Ulva rigida*	569	21.62	358	20.67	32	6.25	50	24.00	0	0.00	24	0.00	34	41.18	45	22.22
infA-62	MW543060	*Ulva rigida*	569	21.62	358	20.67	32	6.25	50	24.00	0	0.00	24	0.00	34	41.18	45	22.22
infA-62	MN853879	*Ulva* sp.	567	21.52	345	21.74	27	0.00	50	24.00	0	0.00	24	0.00	34	41.18	61	18.03
infA-62	MN889540	*Ulva* sp.	567	21.52	345	21.74	27	0.00	50	24.00	0	0.00	24	0.00	34	41.18	61	18.03
infA-62	PV138240	*Ulva* sp.	563	21.49	358	20.95	27	3.70	50	24.00	0	0.00	24	0.00	34	41.18	44	18.18
infA-62	MT179352	*Ulva* sp. A AF-2021	578	21.45	357	21.01	27	7.41	50	26.00	0	0.00	24	0.00	34	41.18	60	16.67
infA-62	MW699788	*Ulva* sp. Q253	567	21.52	345	21.74	27	0.00	50	24.00	0	0.00	24	0.00	34	41.18	61	18.03
infA-62	KP720616	*Ulva* sp. UNA00071828	569	21.97	357	21.29	32	9.38	50	24.00	0	0.00	24	0.00	34	41.18	46	21.74
infA-62	OQ349516	*Ulva taeniata*	563	22.38	356	21.35	27	11.11	50	26.00	0	0.00	24	0.00	34	41.18	46	21.74
infA-62	OL684341	*Ulva tepida*	586	21.50	354	21.47	27	7.41	50	24.00	0	0.00	24	0.00	34	44.12	71	15.49
infA-62	MZ703011	*Ulva torta*	574	21.43	365	20.00	27	3.70	50	26.00	0	0.00	24	0.00	34	41.18	48	25.00
infA-62	OL684342	*Ulva torta*	574	21.25	365	19.73	27	3.70	50	26.00	0	0.00	24	0.00	34	41.18	48	25.00
orf185-47	OL684341	*Ulva tepida*	800	41.12	408	45.59	74	41.89	18	72.22	0	0.00	201	24.38	34	55.88	38	50.00
petB-169	MT179353	*Ulva rigida*	2459	34.85	399	38.35	61	45.90	104	33.65	0	0.00	1796	32.52	34	67.65	35	65.71
petB-169	MW543060	*Ulva rigida*	2459	34.85	399	38.35	61	44.26	104	33.65	0	0.00	1796	32.52	34	67.65	35	65.71
petB-23	OP985133	*Ulva meridionalis*	2317	35.26	323	35.29	43	39.53	86	45.35	0	0.00	1765	34.05	34	47.06	33	42.42
petB-23	MT179353	*Ulva rigida*	2315	34.38	323	35.60	46	34.78	87	41.38	0	0.00	1759	33.26	34	47.06	33	39.39
petB-23	MW543060	*Ulva rigida*	2315	34.34	323	35.60	46	34.78	87	41.38	0	0.00	1759	33.20	34	47.06	33	39.39
petB-23	OQ349516	*Ulva taeniata*	2273	34.76	323	37.15	58	39.66	80	45.00	0	0.00	1712	33.06	34	47.06	33	42.42

Intron name	GenBank accession number	Species	Intron		DI		DII		DIII		DIIIa		DIV		DV		DVI	
			Size (nt)	GC (%)	Size (nt)	GC (%)	Size (nt)	GC (%)	Size (nt)	GC (%)	Size (nt)	GC (%)	Size (nt)	GC (%)	Size (nt)	GC (%)	Size (nt)	GC (%)
petB-277	OR003918	*Ulva dactylifera*	2468	36.10	405	38.77	63	42.86	92	36.96	0	0.00	1809	34.38	34	67.65	35	48.57
petB-277	MW543061	*Ulva lacinulata*	2443	36.47	405	38.27	63	42.86	97	39.18	0	0.00	1779	34.91	34	67.65	35	48.57
petB-277	OP985133	*Ulva meridionalis*	2473	35.91	405	39.01	63	41.27	97	38.14	0	0.00	1809	34.05	34	67.65	35	48.57
petB-277	MT179353	*Ulva rigida*	2442	36.81	400	41.50	62	40.32	97	36.08	0	0.00	1784	34.87	34	67.65	35	51.43
petB-277	MW543060	*Ulva rigida*	2442	36.86	400	41.75	62	40.32	97	36.08	0	0.00	1784	34.87	34	67.65	35	51.43
petB-277	MZ703011	*Ulva torta*	2442	36.08	403	36.72	63	38.10	97	37.11	0	0.00	1780	34.94	34	67.65	35	48.57
petB-277	OL684342	*Ulva torta*	2442	36.08	403	36.72	63	38.10	97	37.11	0	0.00	1780	34.94	34	67.65	35	48.57
petB-69	LC507117	*Ulva australis*	2235	35.12	459	38.34	56	44.64	14	57.14	0	0.00	1594	33.50	34	50.00	43	25.58
petB-69	MT179348	*Ulva australis*	2235	35.12	459	38.34	56	44.64	14	57.14	0	0.00	1594	33.50	34	50.00	43	25.58
petB-69	KX595275	*Ulva compressa*	2227	37.22	428	39.95	70	44.29	14	50.00	0	0.00	1602	35.83	34	52.94	44	31.82
petB-69	MK069584	*Ulva compressa*	2268	34.70	446	35.87	82	34.15	14	57.14	0	0.00	1611	33.95	34	47.06	46	30.43
petB-69	MT916929	*Ulva compressa*	2213	35.25	428	39.95	70	44.29	14	50.00	0	0.00	1588	33.06	34	52.94	44	31.82
petB-69	MW353781	*Ulva compressa*	2227	37.22	428	39.95	70	44.29	14	50.00	0	0.00	1602	35.83	34	52.94	44	31.82
petB-69	MW548841	*Ulva compressa*	2268	34.66	446	35.87	82	34.15	14	57.14	0	0.00	1611	33.89	34	47.06	46	30.43
petB-69	OR003918	*Ulva dactylifera*	2242	34.61	429	39.39	59	38.98	14	50.00	0	0.00	1627	32.82	34	50.00	44	29.55
petB-69	MT179350	*Ulva gigantea*	1826	34.06	431	39.44	69	33.33	14	50.00	0	0.00	1198	31.72	34	47.06	45	28.89
petB-69	MZ158703	*Ulva intestinalis*	2216	37.18	423	39.95	70	42.86	14	50.00	0	0.00	1596	35.84	34	52.94	44	31.82
petB-69	PQ777150	*Ulva intestinalis*	2228	37.21	423	39.95	70	42.86	14	50.00	0	0.00	1608	35.88	34	52.94	44	31.82
petB-69	MN389525	*Ulva lacinulata*	2232	35.80	443	38.37	66	34.85	14	50.00	0	0.00	1602	34.77	34	50.00	38	31.58
petB-69	MT179351	*Ulva lacinulata*	2187	35.76	443	38.37	66	34.85	14	50.00	0	0.00	1557	34.68	34	50.00	38	31.58
petB-69	MW531676	*Ulva lacinulata*	2232	35.75	443	38.37	66	34.85	14	50.00	0	0.00	1602	34.71	34	50.00	38	31.58
petB-69	MW543061	*Ulva lacinulata*	2232	35.71	443	38.37	66	34.85	14	50.00	0	0.00	1602	34.64	34	50.00	38	31.58
petB-69	KT882614	*Ulva lactuca*	2217	35.18	447	39.15	60	41.67	14	50.00	0	0.00	1586	33.29	34	52.94	41	29.27

Intron name	GenBank accession number	Species	Intron		DI		DII		DIII		DIIIa		DIV		DV		DVI	
			Size (nt)	GC (%)	Size (nt)	GC (%)	Size (nt)	GC (%)	Size (nt)	GC (%)	Size (nt)	GC (%)	Size (nt)	GC (%)	Size (nt)	GC (%)	Size (nt)	GC (%)
petB-69	MH730972	*Ulva lactuca*	2208	35.28	447	39.15	60	41.67	14	50.00	0	0.00	1577	33.42	34	52.94	41	29.27
petB-69	PQ824971	*Ulva lactuca*	2208	35.28	447	39.15	60	41.67	14	50.00	0	0.00	1577	33.42	34	52.94	41	29.27
petB-69	AP018696	*Ulva ohnoi*	2222	34.92	438	40.18	59	42.37	14	50.00	0	0.00	1601	32.54	34	52.94	41	31.71
petB-69	MZ571508	*Ulva prolifera*	2257	35.31	459	38.34	61	45.90	14	50.00	0	0.00	1611	33.77	34	50.00	43	25.58
petB-69	KP720616	*Ulva* sp. UNA00071828	2211	35.01	438	40.18	59	47.46	14	50.00	0	0.00	1590	32.52	34	52.94	41	29.27
petB-69	MZ703011	*Ulva torta*	2215	34.40	426	38.97	59	38.98	14	50.00	0	0.00	1603	32.69	34	50.00	44	27.27
petB-69	OL684342	*Ulva torta*	2205	33.97	426	38.97	59	38.98	14	50.00	0	0.00	1593	32.08	34	50.00	44	27.27
petD-87	KX595275	*Ulva compressa*	2420	36.20	383	39.16	27	44.44	18	61.11	0	0.00	1888	34.75	34	47.06	47	46.81
petD-87	MW344287	*Ulva compressa*	2444	36.25	383	39.16	27	44.44	18	61.11	0	0.00	1912	34.83	34	47.06	47	46.81
petD-87	MW353781	*Ulva compressa*	2420	36.20	383	39.16	27	44.44	18	61.11	0	0.00	1888	34.75	34	47.06	47	46.81
petD-87	MT179350	*Ulva gigantea*	2427	36.26	381	40.94	27	44.44	18	55.56	0	0.00	1900	34.47	34	44.12	44	52.27
psbC-496	MK069584	*Ulva compressa*	2441	36.71	389	35.99	66	36.36	66	36.36	0	0.00	1798	36.32	35	60.00	64	34.38
atp1-1095	MT179359	*Ulva rigida*	2547	43.46	346	50.58	23	39.13	82	45.12	0	0.00	1990	41.76	34	61.76	46	43.48
atp1-1316	MN853878	*Ulva* sp.	3081	45.70	406	51.72	93	47.31	38	39.47	0	0.00	2445	44.54	34	52.94	38	44.74
atp1-990	ON402236	*Ulva meridionalis*	2583	49.28	455	49.89	28	67.86	42	64.29	30	53.33	1927	48.37	34	58.82	38	44.74
atp1-990	ON402237	*Ulva meridionalis*	2583	49.28	455	49.89	28	67.86	42	64.29	30	53.33	1927	48.37	34	58.82	38	44.74
atp1-990	ON402238	*Ulva meridionalis*	2583	49.25	455	49.89	28	67.86	42	64.29	30	53.33	1927	48.31	34	58.82	38	44.74
atp1-990	ON402239	*Ulva meridionalis*	2583	49.25	455	49.89	28	67.86	42	64.29	30	53.33	1927	48.31	34	58.82	38	44.74
atp1-990	ON402240	*Ulva meridionalis*	2583	49.28	455	49.89	28	67.86	42	64.29	30	53.33	1927	48.37	34	58.82	38	44.74
atp1-990	AP018695	*Ulva ohnoi*	2589	48.71	455	48.57	28	53.57	42	61.90	30	43.33	1933	48.32	34	55.88	38	44.74
atp1-990	MN853878	*Ulva* sp.	2583	48.20	455	48.79	28	64.29	42	64.29	30	53.33	1927	47.17	34	55.88	38	47.37
cob-877	OR030801	*Ulva taeniata*	2331	45.99	377	50.40	24	66.67	40	55.00	0	0.00	1781	44.41	34	50.00	52	40.38

Intron name	GenBank accession number	Species	Intron		DI		DII		DIII		DIIIa		DIV		DV		DVI	
			Size (nt)	GC (%)	Size (nt)	GC (%)	Size (nt)	GC (%)	Size (nt)	GC (%)	Size (nt)	GC (%)	Size (nt)	GC (%)	Size (nt)	GC (%)	Size (nt)	GC (%)
cox1-199	MK069586	*Ulva compressa*	2501	47.06	381	51.44	57	63.16	41	43.90	0	0.00	1931	45.05	34	44.12	37	75.68
cox1-199	MH013470	*Ulva aragoënsis*	2495	45.61	381	51.97	57	57.89	39	46.15	0	0.00	1927	43.18	34	47.06	37	72.97
cox1-199	KU189740	*Ulva linza*	2525	44.87	380	50.26	56	58.93	38	47.37	0	0.00	1960	42.55	34	47.06	37	72.97
cox1-199	MN853878	*Ulva* sp.	2476	44.67	380	50.00	54	64.81	34	50.00	0	0.00	1917	42.10	34	47.06	37	72.97
cox1-199	OR030801	*Ulva taeniata*	2466	44.65	377	51.19	58	50.00	36	50.00	0	0.00	1904	42.23	34	47.06	37	72.97
cox1-643	MH013469	*Ulva compressa*	2508	45.85	413	47.70	22	81.82	32	62.50	22	54.55	1881	44.39	34	64.71	71	39.44
cox1-643	MK069587	*Ulva compressa*	2508	45.85	413	47.70	22	81.82	32	62.50	22	54.55	1881	44.39	34	64.71	71	39.44
cox1-643	MT179355	*Ulva fenestrata*	2517	45.09	407	49.14	22	81.82	35	60.00	22	45.45	1886	42.90	34	61.76	78	50.00
cox1-686	MN853878	*Ulva* sp.	789	40.81	333	46.25	121	32.79	194	38.14	0	0.00	38	57.89	34	58.82	31	29.03
cox1-760	KX530816	*Ulva australis*	2604	46.93	372	49.46	39	12.82	86	52.33	30	43.33	1947	46.84	34	55.88	65	43.08
cox1-760	MH013470	*Ulva aragoënsis*	2692	43.57	444	45.05	45	6.67	86	53.49	27	33.33	1963	43.61	34	55.88	62	38.71
cox1-760	MT179359	*Ulva rigida*	2627	46.52	379	48.81	55	12.73	86	53.49	30	46.67	1947	46.64	34	55.88	65	41.54
cox1-874	MH730971	*Ulva expansa*	1157	37.86	411	41.61	34	38.24	90	36.67	0	0.00	522	35.06	32	46.88	40	25.00
cox1-874	KX455878	*Ulva aragoënsis*	1591	33.88	398	39.70	35	34.29	86	47.67	0	0.00	972	29.73	32	46.88	40	30.00
cox1-874	KY626326	*Ulva aragoënsis*	1591	34.00	398	39.95	35	37.14	86	47.67	0	0.00	972	29.73	32	46.88	40	30.00
cox1-874	MH013470	*Ulva aragoënsis*	1506	34.26	402	39.80	35	37.14	90	40.00	0	0.00	879	30.38	32	46.88	40	30.00
cox1-874	MT179356	*Ulva gigantea*	1424	31.74	373	42.90	35	40.00	90	37.78	0	0.00	826	24.58	32	56.25	40	25.00
cox1-874	MZ571476	*Ulva intestinalis*	1510	34.50	398	39.70	35	37.14	90	40.00	0	0.00	887	30.89	32	46.88	40	30.00
cox1-874	MN389526	*Ulva lacinulata*	1662	34.12	380	42.89	35	42.86	178	31.46	0	0.00	969	30.34	32	46.88	40	30.00
cox1-874	MT179357	*Ulva lacinulata*	1667	34.07	380	42.89	35	42.86	178	31.46	0	0.00	974	30.29	32	46.88	40	30.00
cox1-874	KU189740	*Ulva linza*	1509	34.53	398	39.70	35	37.14	90	40.00	0	0.00	886	30.93	32	46.88	40	30.00
cox1-874	ON402236	*Ulva meridionalis*	1493	33.42	389	37.53	31	41.94	90	40.00	0	0.00	883	29.78	32	56.25	40	25.00
cox1-874	ON402237	*Ulva meridionalis*	1493	33.42	389	37.53	31	41.94	90	40.00	0	0.00	883	29.78	32	56.25	40	25.00

Intron name	GenBank accession number	Species	Intron		DI		DII		DIII		DIIIa		DIV		DV		DVI	
			Size (nt)	GC (%)	Size (nt)	GC (%)	Size (nt)	GC (%)	Size (nt)	GC (%)	Size (nt)	GC (%)	Size (nt)	GC (%)	Size (nt)	GC (%)	Size (nt)	GC (%)
cox1-874	ON402238	*Ulva meridionalis*	1493	33.42	389	37.53	31	41.94	90	40.00	0	0.00	883	29.78	32	56.25	40	25.00
cox1-874	ON402239	*Ulva meridionalis*	1493	33.42	389	37.53	31	41.94	90	40.00	0	0.00	883	29.78	32	56.25	40	25.00
cox1-874	ON402240	*Ulva meridionalis*	1493	33.42	389	37.53	31	41.94	90	40.00	0	0.00	883	29.78	32	56.25	40	25.00
cox1-874	KT428794	*Ulva prolifera*	1509	34.53	398	39.70	35	37.14	90	40.00	0	0.00	886	30.93	32	46.88	40	30.00
cox1-874	KU161104	*Ulva prolifera*	1509	34.53	398	39.70	35	37.14	90	40.00	0	0.00	886	30.93	32	46.88	40	30.00
cox1-874	MZ438677	*Ulva prolifera*	1509	34.53	398	39.70	35	37.14	90	40.00	0	0.00	886	30.93	32	46.88	40	30.00
cox1-874	PV023351	*Ulva prolifera*	1509	34.53	398	39.70	35	37.14	90	40.00	0	0.00	886	30.93	32	46.88	40	30.00
cox1-874	MT179359	*Ulva rigida*	1621	32.57	414	35.51	39	33.33	166	36.14	0	0.00	902	30.04	32	46.88	40	25.00
cox1-874	MN853878	*Ulva* sp.	1904	35.24	392	38.78	32	31.25	201	33.33	0	0.00	1179	34.27	32	46.88	40	27.50
cox1-874	PV023352	*Ulva taeniata*	1489	36.00	380	42.63	35	42.86	90	37.78	0	0.00	884	32.47	32	46.88	40	27.50
cox2-424	KX530816	*Ulva australis*	2463	45.51	436	51.38	29	51.72	40	47.50	0	0.00	1843	43.24	34	58.82	60	51.67
cox2-424	KX595276	*Ulva compressa*	2581	48.12	508	53.35	29	55.17	40	37.50	0	0.00	1898	46.10	34	58.82	51	58.82
cox2-424	KY626327	*Ulva compressa*	2581	48.08	508	53.15	29	55.17	40	37.50	0	0.00	1898	46.10	34	58.82	51	58.82
cox2-424	MH013469	*Ulva compressa*	2561	48.11	508	53.54	29	55.17	40	37.50	0	0.00	1878	46.01	34	58.82	51	58.82
cox2-424	MK069586	*Ulva compressa*	2581	48.08	508	53.15	29	55.17	40	37.50	0	0.00	1898	46.10	34	58.82	51	58.82
cox2-424	MK069587	*Ulva compressa*	2560	48.12	508	53.54	29	55.17	40	37.50	0	0.00	1877	46.03	34	58.82	51	58.82
cox2-424	MT179355	*Ulva fenestrata*	2434	45.89	397	50.88	29	51.72	40	52.50	0	0.00	1852	43.63	34	58.82	61	59.02
cox2-424	MT179356	*Ulva gigantea*	2480	47.54	436	51.38	29	58.62	37	48.65	0	0.00	1872	45.73	34	58.82	51	56.86
cox2-424	AP018695	*Ulva ohnoi*	2539	43.80	498	47.59	29	48.28	40	40.00	0	0.00	1861	41.97	34	58.82	56	51.79
cox2-424	MT179359	*Ulva rigida*	2541	43.60	500	47.40	29	41.38	40	40.00	0	0.00	1861	41.97	34	58.82	56	48.21
cox2-424	MN853878	*Ulva* sp.	2564	45.05	507	47.73	29	48.28	40	35.00	0	0.00	1867	43.71	34	58.82	66	51.52
cox2-424	MT179358	*Ulva* sp. A AF-2021	2463	44.86	433	50.35	29	44.83	40	45.00	0	0.00	1849	42.94	34	58.82	57	47.37

Intron name	GenBank accession number	Species	Intron		DI		DII		DIII		DIIIa		DIV		DV		DVI	
			Size (nt)	GC (%)	Size (nt)	GC (%)	Size (nt)	GC (%)	Size (nt)	GC (%)	Size (nt)	GC (%)	Size (nt)	GC (%)	Size (nt)	GC (%)	Size (nt)	GC (%)
cox2-424	OR030801	*Ulva taeniata*	2373	44.63	451	50.11	29	48.28	40	45.00	0	0.00	1746	42.27	34	58.82	52	55.77
cox2-424	MH013471	*Ulva torta*	2460	45.69	454	49.78	29	51.72	40	47.50	0	0.00	1830	43.72	34	58.82	52	57.69
cox2-751	KX530816	*Ulva australis*	2380	46.89	464	51.29	54	46.30	89	49.44	0	0.00	1680	45.36	34	55.88	33	48.48
cox2-751	KX530817	*Ulva australis*	2380	46.89	464	51.29	54	46.30	89	49.44	0	0.00	1680	45.36	34	55.88	33	48.48
cox2-751	MT179354	*Ulva australis*	2380	46.89	464	51.29	54	46.30	89	49.44	0	0.00	1680	45.36	34	55.88	33	48.48
cox2-751	KX595276	*Ulva compressa*	2460	48.78	458	52.18	55	49.09	88	52.27	0	0.00	1766	47.45	34	70.59	33	42.42
cox2-751	KY626327	*Ulva compressa*	2460	48.74	458	51.97	55	49.09	88	52.27	0	0.00	1766	47.45	34	70.59	33	42.42
cox2-751	MH093740	*Ulva compressa*	2459	48.84	457	52.74	55	49.09	88	53.41	0	0.00	1766	47.34	34	70.59	33	42.42
cox2-751	MK069586	*Ulva compressa*	2460	48.74	458	51.97	55	49.09	88	52.27	0	0.00	1766	47.45	34	70.59	33	42.42
cox2-751	MK069587	*Ulva compressa*	2626	49.39	457	52.08	55	49.09	260	59.23	0	0.00	1761	47.02	34	70.59	33	42.42
cox2-751	ON402236	*Ulva meridionalis*	2474	47.62	458	49.78	53	58.49	88	51.14	0	0.00	1782	46.18	34	70.59	33	48.48
cox2-751	ON402237	*Ulva meridionalis*	2474	47.62	458	49.78	53	58.49	88	51.14	0	0.00	1782	46.18	34	70.59	33	48.48
cox2-751	ON402238	*Ulva meridionalis*	2474	47.62	458	49.78	53	58.49	88	51.14	0	0.00	1782	46.18	34	70.59	33	48.48
cox2-751	ON402239	*Ulva meridionalis*	2474	47.62	458	49.78	53	58.49	88	51.14	0	0.00	1782	46.18	34	70.59	33	48.48
cox2-751	ON402240	*Ulva meridionalis*	2474	47.62	458	49.78	53	58.49	88	51.14	0	0.00	1782	46.18	34	70.59	33	48.48
cox2-751	MT179359	*Ulva rigida*	2411	48.40	520	54.81	54	62.96	88	44.32	0	0.00	1656	45.83	34	64.71	33	48.48
cox2-751	MN853878	*Ulva* sp.	2450	46.08	449	47.88	53	54.72	89	49.44	0	0.00	1766	44.73	34	67.65	33	48.48
cox2-751	OR030801	*Ulva taeniata*	2050	44.73	484	50.21	51	54.90	88	50.00	0	0.00	1334	41.45	34	64.71	33	48.48
cox2-751	MH013471	*Ulva torta*	2428	46.66	458	49.13	68	48.53	89	51.69	0	0.00	1720	45.35	34	67.65	33	45.45
nad3-215	KP720617	*Ulva* sp. UNA00071828	2727	40.15	419	48.21	55	54.55	187	44.39	0	0.00	1975	37.16	35	51.43	33	51.52
nad3-216	MH013469	*Ulva compressa*	2542	41.70	386	47.93	55	47.27	164	48.17	0	0.00	1864	39.27	35	51.43	18*	61.11*

Intron name	GenBank accession number	Species	Intron		DI		DII		DIII		DIIIa		DIV		DV		DVI	
			Size (nt)	GC (%)	Size (nt)	GC (%)	Size (nt)	GC (%)	Size (nt)	GC (%)	Size (nt)	GC (%)	Size (nt)	GC (%)	Size (nt)	GC (%)	Size (nt)	GC (%)
nad3-216	MK069586	*Ulva compressa*	2507	45.47	384	48.44	55	47.27	164	48.17	0	0.00	1813	44.35	35	51.43	33	51.52
nad3-216	MK069587	*Ulva compressa*	2560	41.68	386	47.93	55	47.27	164	48.17	0	0.00	1864	39.27	35	51.43	33	51.52
nad3-216	OR030800	*Ulva dactylifera*	2480	41.98	371	45.28	54	44.44	158	45.57	0	0.00	1806	40.48	35	51.43	33	51.52
nad3-216	MT179356	*Ulva gigantea*	1634	43.33	381	48.29	55	47.27	176	46.59	0	0.00	931	39.96	35	51.43	33	48.48
nad3-216	KT364296	*Ulva lactuca*	2481	42.40	376	46.81	54	50.00	157	45.86	0	0.00	1803	40.71	35	51.43	33	45.45
nad3-216	KU182748	*Ulva lactuca*	2481	42.44	376	46.81	54	50.00	157	45.86	0	0.00	1803	40.77	35	51.43	33	45.45
nad3-216	MH763013	*Ulva lactuca*	2481	42.44	376	46.81	54	50.00	157	45.86	0	0.00	1803	40.77	35	51.43	33	45.45
nad3-216	KU189740	*Ulva linza*	2516	42.05	383	47.78	55	45.45	156	45.51	0	0.00	1831	40.09	35	51.43	33	51.52
nad3-216	ON402236	*Ulva meridionalis*	2519	45.53	430	48.37	57	59.65	125	48.80	0	0.00	1816	43.83	35	51.43	33	57.58
nad3-216	ON402237	*Ulva meridionalis*	2519	45.53	430	48.37	57	59.65	125	48.80	0	0.00	1816	43.83	35	51.43	33	57.58
nad3-216	ON402238	*Ulva meridionalis*	2514	45.62	430	48.37	57	59.65	125	48.80	0	0.00	1811	43.95	35	51.43	33	57.58
nad3-216	ON402239	*Ulva meridionalis*	2514	45.62	430	48.37	57	59.65	125	48.80	0	0.00	1811	43.95	35	51.43	33	57.58
nad3-216	ON402240	*Ulva meridionalis*	2518	45.59	430	48.37	57	59.65	125	48.80	0	0.00	1815	43.91	35	51.43	33	57.58
nad3-216	AP018695	*Ulva ohnoi*	2498	45.32	426	49.06	57	56.14	123	51.22	0	0.00	1801	43.36	35	51.43	33	57.58
nad3-216	KT428794	*Ulva prolifera*	2503	42.15	383	47.78	55	45.45	156	45.51	0	0.00	1818	40.21	35	51.43	33	51.52
nad3-216	KU161104	*Ulva prolifera*	2503	42.11	383	47.78	55	45.45	156	45.51	0	0.00	1818	40.15	35	51.43	33	51.52
nad3-216	MZ438677	*Ulva prolifera*	2503	42.15	383	47.78	55	45.45	156	45.51	0	0.00	1818	40.21	35	51.43	33	51.52
nad3-216	PV023351	*Ulva prolifera*	2508	42.03	383	47.78	55	45.45	156	45.51	0	0.00	1823	40.04	35	51.43	33	51.52
nad3-216	MT179359	*Ulva rigida*	2513	44.25	375	48.27	54	53.70	135	45.19	0	0.00	1858	42.84	35	51.43	33	51.52
nad3-216	MT179358	*Ulva* sp. A AF-2021	2456	44.67	377	48.01	54	51.85	133	43.61	0	0.00	1801	43.59	35	54.29	33	48.48
nad3-216	OR030801	*Ulva taeniata*	2254	41.22	375	47.47	54	48.15	151	48.34	0	0.00	1583	38.34	35	51.43	33	51.52
nad3-216	PV023352	*Ulva taeniata*	2453	44.56	377	47.75	54	51.85	133	43.61	0	0.00	1798	43.49	35	54.29	33	48.48

Intron name	GenBank accession number	Species	Intron		DI		DII		DIII		DIIIa		DIV		DV		DVI	
			Size (nt)	GC (%)	Size (nt)	GC (%)	Size (nt)	GC (%)	Size (nt)	GC (%)	Size (nt)	GC (%)	Size (nt)	GC (%)	Size (nt)	GC (%)	Size (nt)	GC (%)
nad5-1057	MT179355	*Ulva fenestrata*	2649	45.83	434	44.01	28	42.86	83	46.99	66	54.55	1911	45.47	34	61.76	64	46.88
nad5-1057	MT179359	*Ulva rigida*	2631	45.61	426	45.31	28	46.43	86	46.51	24	58.33	1920	45.31	34	64.71	84	36.90
rnl-1963	KX530817	*Ulva australis*	2364	41.29	355	43.66	55	47.27	78	50.00	0	0.00	1778	39.76	34	44.12	35	65.71
rnl-1963	MT179354	*Ulva australis*	2364	41.29	355	43.66	55	47.27	78	50.00	0	0.00	1778	39.76	34	44.12	35	65.71
rnl-1963	MH730971	*Ulva expansa*	1681	42.47	375	44.00	93	49.46	78	48.72	0	0.00	1037	40.31	34	44.12	35	60.00
rnl-1963	MT179356	*Ulva gigantea*	1250	43.28	358	44.41	56	48.21	78	44.87	0	0.00	660	41.21	34	47.06	35	60.00
rnl-1963	KX530816	*Ulva pertusa*	2364	41.29	355	43.66	55	47.27	78	50.00	0	0.00	1778	39.76	34	44.12	35	65.71
rnl-1963	OR030801	*Ulva taeniata*	2284	41.59	356	44.94	55	45.45	78	42.31	0	0.00	1697	40.19	34	44.12	35	60.00
rnl-2080	MH730971	*Ulva expansa*	1546	38.42	335	42.09	14	71.43	176	36.93	0	0.00	925	36.00	34	44.12	31	54.84
rnl-2080	KX455878	*Ulva aragoënsis*	1550	38.13	334	43.41	14	71.43	175	36.57	0	0.00	931	34.91	34	47.06	31	54.84
rnl-2080	KY626326	*Ulva aragoënsis*	1550	37.94	333	43.24	14	71.43	174	36.21	0	0.00	933	34.73	34	47.06	31	54.84
rnl-2080	MN389526	*Ulva lacinulata*	1554	37.64	328	40.24	14	71.43	183	37.70	0	0.00	933	35.26	34	47.06	31	54.84
rnl-2080	MT179357	*Ulva lacinulata*	1554	37.64	328	40.24	14	71.43	183	37.70	0	0.00	933	35.26	34	47.06	31	54.84
rnl-2080	MN861072	*Ulva meridionalis*	1545	37.73	328	42.07	14	71.43	175	36.57	0	0.00	932	34.98	34	47.06	31	48.39
rnl-2080	MN853878	*Ulva* sp.	1663	37.88	355	41.41	19	57.89	243	37.45	0	0.00	950	35.37	34	47.06	31	48.39
rnl-2080	MT179358	*Ulva* sp. A AF-2021	1551	37.78	328	40.24	14	71.43	183	37.70	0	0.00	930	35.38	34	47.06	31	54.84
rnl-2080	PV023352	*Ulva taeniata*	1542	37.55	328	40.24	14	71.43	183	37.70	0	0.00	921	35.07	34	47.06	31	54.84
rnl-2698	KX455878	*Ulva aragoënsis*	1380	37.39	258	43.02	70	41.43	81	39.51	0	0.00	870	34.37	34	58.82	33	42.42
rnl-2698	KY626326	*Ulva aragoënsis*	1380	37.39	258	43.02	70	41.43	81	39.51	0	0.00	870	34.37	34	58.82	33	42.42
rnl-2698	MZ571476	*Ulva intestinalis*	1359	36.42	258	40.70	65	38.46	81	39.51	0	0.00	854	33.37	34	58.82	33	45.45
rnl-2698	MN389526	*Ulva lacinulata*	1363	36.46	258	40.70	65	40.00	81	39.51	0	0.00	858	33.33	34	58.82	33	45.45
rnl-2698	MT179357	*Ulva lacinulata*	1359	36.50	258	40.70	65	40.00	81	39.51	0	0.00	854	33.37	34	58.82	33	45.45

Intron name	GenBank accession number	Species	Intron		DI		DII		DIII		DIIIa		DIV		DV		DVI	
			Size (nt)	GC (%)	Size (nt)	GC (%)	Size (nt)	GC (%)	Size (nt)	GC (%)	Size (nt)	GC (%)	Size (nt)	GC (%)	Size (nt)	GC (%)	Size (nt)	GC (%)
rnl-2698	KU189740	*Ulva linza*	1393	35.53	287	39.72	74	36.49	81	34.57	0	0.00	850	32.82	34	58.82	33	48.48
rnl-2698	MN861072	*Ulva meridionalis*	1375	36.80	258	42.25	70	41.43	81	39.51	0	0.00	866	34.41	34	58.82	33	30.30
rnl-2698	MN853878	*Ulva* sp.	1427	37.21	257	40.86	79	43.04	81	41.98	0	0.00	910	34.95	34	58.82	33	33.33
rnl-2698	MT179358	*Ulva* sp. A AF-2021	1359	36.50	258	40.70	65	40.00	81	39.51	0	0.00	854	33.37	34	58.82	33	45.45
rnl-2698	PV023352	*Ulva taeniata*	1359	36.50	258	40.70	65	40.00	81	39.51	0	0.00	854	33.37	34	58.82	33	45.45
rns-420	MH730971	*Ulva expansa*	619	39.42	260	42.31	70	40.00	81	38.27	0	0.00	116	26.72	34	58.82	33	48.48
rns-420	KX455878	*Ulva aragoënsis*	1368	35.96	260	38.08	70	41.43	81	38.27	0	0.00	865	33.41	34	58.82	33	48.48
rns-420	KY626326	*Ulva aragoënsis*	1368	35.96	260	38.08	70	41.43	81	38.27	0	0.00	865	33.41	34	58.82	33	48.48
rns-420	MZ571476	*Ulva intestinalis*	1338	35.87	260	38.46	78	33.33	81	40.74	0	0.00	827	33.49	34	58.82	33	48.48
rns-420	MN389526	*Ulva lacinulata*	686	38.92	260	40.00	72	37.50	81	39.51	0	0.00	180	32.22	34	58.82	34	52.94
rns-420	MT179357	*Ulva lacinulata*	686	38.92	260	40.00	72	37.50	81	39.51	0	0.00	180	32.22	34	58.82	34	52.94
rns-420	KU189740	*Ulva linza*	1402	34.81	260	39.62	74	37.84	81	34.57	0	0.00	895	31.73	34	58.82	33	51.52
rns-420	MN861072	*Ulva meridionalis*	1368	35.96	260	38.08	70	41.43	81	38.27	0	0.00	865	33.41	34	58.82	33	48.48
rns-420	ON402236	*Ulva meridionalis*	1329	33.11	266	33.83	76	40.79	81	30.86	0	0.00	803	30.88	34	55.88	37	48.65
rns-420	ON402237	*Ulva meridionalis*	1329	33.11	266	33.83	76	40.79	81	30.86	0	0.00	803	30.88	34	55.88	37	48.65
rns-420	ON402238	*Ulva meridionalis*	1329	33.11	266	33.83	76	40.79	81	30.86	0	0.00	803	30.88	34	55.88	37	48.65
rns-420	ON402239	*Ulva meridionalis*	1329	33.11	266	33.83	76	40.79	81	30.86	0	0.00	803	30.88	34	55.88	37	48.65
rns-420	ON402240	*Ulva meridionalis*	1329	33.11	266	33.83	76	40.79	81	30.86	0	0.00	803	30.88	34	55.88	37	48.65
rns-420	MT179358	*Ulva* sp. A AF-2021	1387	36.63	259	40.93	142	37.32	81	39.51	0	0.00	813	33.46	34	58.82	33	51.52
rns-420	KP720617	*Ulva* sp. UNA00071828	1319	32.22	274	32.48	64	40.62	81	41.98	0	0.00	798	28.57	34	55.88	37	51.35

Intron name	GenBank accession number	Species	Intron		DI		DII		DIII		DIIIa		DIV		DV		DVI	
			Size (nt)	GC (%)	Size (nt)	GC (%)	Size (nt)	GC (%)	Size (nt)	GC (%)	Size (nt)	GC (%)	Size (nt)	GC (%)	Size (nt)	GC (%)	Size (nt)	GC (%)
rns-420	OR030801	*Ulva taeniata*	1261	34.89	260	37.69	70	41.43	81	38.27	0	0.00	758	31.40	34	58.82	33	48.48
rns-420	PV023352	*Ulva taeniata*	685	38.69	260	40.00	72	37.50	81	39.51	0	0.00	180	32.22	34	58.82	33	51.52
rns-670	MH730971	*Ulva expansa*	623	40.13	375	39.20	51	45.10	55	41.82	0	0.00	48	22.92	34	47.06	33	54.55
rns-670	KX455878	*Ulva aragoënsis*	1004	34.56	341	38.42	51	47.06	55	40.00	0	0.00	463	27.00	34	47.06	33	51.52
rns-670	KY626326	*Ulva aragoënsis*	1004	34.56	341	38.42	51	47.06	55	40.00	0	0.00	463	27.00	34	47.06	33	51.52
rns-670	MH013470	*Ulva aragoënsis*	1490	33.76	341	38.71	51	47.06	55	40.00	0	0.00	949	29.50	34	47.06	33	51.52
rns-670	KU189740	*Ulva linza*	1481	34.84	341	39.00	51	47.06	55	41.82	0	0.00	940	31.17	34	47.06	33	45.45
rns-670	MN861072	*Ulva meridionalis*	1049	35.08	371	39.08	51	50.98	55	41.82	0	0.00	478	27.41	34	47.06	33	48.48
rns-670	ON402236	*Ulva meridionalis*	1528	35.01	357	38.10	50	50.00	55	41.82	0	0.00	970	31.65	34	47.06	33	48.48
rns-670	ON402237	*Ulva meridionalis*	1528	35.01	357	38.10	50	50.00	55	41.82	0	0.00	970	31.65	34	47.06	33	48.48
rns-670	ON402238	*Ulva meridionalis*	1528	35.01	357	38.10	50	50.00	55	41.82	0	0.00	970	31.65	34	47.06	33	48.48
rns-670	ON402239	*Ulva meridionalis*	1528	35.01	357	38.10	50	50.00	55	41.82	0	0.00	970	31.65	34	47.06	33	48.48
rns-670	ON402240	*Ulva meridionalis*	1528	35.01	357	38.10	50	50.00	55	41.82	0	0.00	970	31.65	34	47.06	33	48.48
rns-670	KT428794	*Ulva prolifera*	1098	34.79	341	39.00	51	47.06	55	41.82	0	0.00	557	28.55	34	47.06	33	45.45
rns-670	KU161104	*Ulva prolifera*	1098	34.79	341	39.00	51	47.06	55	41.82	0	0.00	557	28.55	34	47.06	33	45.45
rns-670	MZ438677	*Ulva prolifera*	1098	34.79	341	39.00	51	47.06	55	41.82	0	0.00	557	28.55	34	47.06	33	45.45
rns-670	PV023351	*Ulva prolifera*	1098	34.79	341	39.00	51	47.06	55	41.82	0	0.00	557	28.55	34	47.06	33	45.45
rns-670	KP720617	*Ulva* sp.	1534	35.27	368	41.30	51	41.18	55	41.82	0	0.00	966	31.26	34	47.06	33	48.48
rns-670	MN853878	*Ulva* sp.	1536	34.51	371	39.08	51	50.98	55	41.82	0	0.00	965	30.36	34	47.06	33	48.48
rns-670	PP908992	*Ulva* sp.	1519	34.50	365	39.45	51	47.06	55	40.00	0	0.00	954	30.40	34	47.06	33	48.48
rns-670	MT179358	*Ulva* sp. A AF-2021	830	36.27	341	39.59	51	49.02	55	43.64	0	0.00	289	25.26	34	47.06	33	48.48
rns-670	MH013467	*Ulva* sp. TM637	1424	34.79	365	39.29	51	47.06	55	40.00	0	0.00	859	30.50	34	47.06	33	48.48

Intron name	GenBank accession number	Species	Intron		DI		DII		DIII		DIIIa		DIV		DV		DVI	
			Size (nt)	GC (%)	Size (nt)	GC (%)	Size (nt)	GC (%)	Size (nt)	GC (%)	Size (nt)	GC (%)	Size (nt)	GC (%)	Size (nt)	GC (%)	Size (nt)	GC (%)
rns-780	KX530817	*Ulva australis*	2415	47.70	312	49.68	64	57.81	78	44.87	0	0.00	1867	46.65	34	55.88	33	72.73
rns-780	MT179354	*Ulva australis*	2415	47.70	312	49.68	64	57.81	78	44.87	0	0.00	1867	46.65	34	55.88	33	72.73
rns-780	MK069587	*Ulva compressa*	2438	47.50	312	49.68	65	58.46	78	44.87	0	0.00	1890	46.40	34	55.88	32	71.88
rns-780	AP018695	*Ulva ohnoi*	1028	46.79	308	48.05	59	59.32	78	41.03	0	0.00	489	44.17	34	55.88	33	66.67
rns-780	KX530816	*Ulva pertusa*	2415	47.70	312	49.68	64	57.81	78	44.87	0	0.00	1867	46.65	34	55.88	33	72.73
rns-780	KP720617	*Ulva* sp. UNA00071828	2449	45.41	309	48.22	58	62.07	83	44.58	0	0.00	1905	44.09	34	55.88	33	66.67

*The region of this intron was not fully statistically analyzed due to significant sequence differences.
