# Supplementary material for: Structural Remodeling and Enzymatic Replacement Shape the Evolution of Organellar Group II Introns in Ulva
Source: Int J Mol Sci. 2026 Mar 12;27(6):2613. doi: 10.3390/ijms27062613 (PMC13026550; doi:10.3390/ijms27062613)
Supplement: Supplementary file 1 [file ijms-27-02613-s001.zip › Supplementary Table S6. terminal motifs and linker.pdf]

Table S6. The 5′ and 3′ terminal motifs and 5′ terminal linker of group II introns detected in organellar genomes of *Ulva* species.

Intron name	Intron type	Species	GenBank accession number	5′ terminal linker (5′-3′)	5′ terminal motif (5-GUGCGA-3′)	3′ terminal motif (5′-AY-3′)	Changes of 5′ and 3′ terminal motifs (GUGCGA/AY)
atp1-1316	mt IIA2-RT/M	*Ulva* sp.	MN853878		GUGGGA	CU	Both 5′ and 3′ terminal motif change
psbC-496	cp IIB2-RT/M	*Ulva compressa*	MK069584		UUGCGA	GC	Both 5′ and 3′ terminal motif change
cob-877	mt IIA1-RT/M	*Ulva taeniata*	OR030801		GCGCGC	AG	Both 5′ and 3′ terminal motif change
atpI-256	cp IIB2-RT/M	*Ulva gigantea*	MT179350		GUGCGG	CC	Both 5′ and 3′ terminal motif change
cox1-874	mt IIB2-LHE	*Ulva prolifera*	KT428794		GUGCGA	AG	Both 5′ and 3′ terminal motif change
cox1-874	mt IIB2-LHE	*Ulva prolifera*	KU161104		GUGCGA	AG	Both 5′ and 3′ terminal motif change
cox1-874	mt IIB2-LHE	*Ulva linza*	KU189740		GUGCGA	AG	Both 5′ and 3′ terminal motif change
cox1-874	mt IIB2-LHE	*Ulva aragoënsis*	KX455878		GUGCGA	AG	Both 5′ and 3′ terminal motif change
cox1-874	mt IIB2-LHE	*Ulva aragoënsis*	KY626326		GUGCGA	AG	Both 5′ and 3′ terminal motif change
cox1-874	mt IIB2-LHE	*Ulva aragoënsis*	MH013470		GUGCGA	AG	Both 5′ and 3′ terminal motif change
cox1-874	mt IIB2-LHE	*Ulva expansa*	MH730971		GUGCGA	AG	Both 5′ and 3′ terminal motif change
cox1-874	mt IIB2-LHE	*Ulva lacinulata*	MN389526		GUGUGA	AG	Both 5′ and 3′ terminal motif change
cox1-874	mt IIB2-LHE	*Ulva* sp.	MN853878		GUGCGA	AG	Both 5′ and 3′ terminal motif change
cox1-874	mt IIB2-LHE	*Ulva gigantea*	MT179356		GUGCGA	AG	Both 5′ and 3′ terminal motif change
cox1-874	mt IIB2-LHE	*Ulva lacinulata*	MT179357		GUGUGA	AG	Both 5′ and 3′ terminal motif change
cox1-874	mt IIB2-LHE	*Ulva rigida*	MT179359		GUGUGA	AG	Both 5′ and 3′ terminal motif change
cox1-874	mt IIB2-LHE	*Ulva prolifera*	MZ438677		GUGCGA	AG	Both 5′ and 3′ terminal motif change
cox1-874	mt IIB2-LHE	*Ulva intestinalis*	MZ571476		GUGCGA	AG	Both 5′ and 3′ terminal motif change
cox1-874	mt IIB2-LHE	*Ulva meridionalis*	ON402236		GUGCGA	AG	Both 5′ and 3′ terminal motif change
cox1-874	mt IIB2-LHE	*Ulva meridionalis*	ON402237		GUGCGA	AG	Both 5′ and 3′ terminal motif change
cox1-874	mt IIB2-LHE	*Ulva meridionalis*	ON402238		GUGCGA	AG	Both 5′ and 3′ terminal motif change
cox1-874	mt IIB2-LHE	*Ulva meridionalis*	ON402239		GUGCGA	AG	Both 5′ and 3′ terminal motif change
cox1-874	mt IIB2-LHE	*Ulva meridionalis*	ON402240		GUGCGA	AG	Both 5′ and 3′ terminal motif change

Intron name	Intron type	Species	GenBank accession number	5′ terminal linker (5′-3′)	5′ terminal motif (5-GUGCGA-3′)	3′ terminal motif (5′-AY-3′)	Changes of 5′ and 3′ terminal motifs (GUGCGA/AY)
cox1-874	mt IIB2-LHE	*Ulva prolifera*	PV023351		GUGCGA	AG	Both 5′ and 3′ terminal motif change
cox1-874	mt IIB2-LHE	*Ulva taeniata*	PV023352		GUGUGA	AG	Both 5′ and 3′ terminal motif change
rns-420	mt IIB1-LHE	*Ulva* sp. UNA00071828	KP720617	AAUAAAA	ACGCGA	GC	Both 5′ and 3′ terminal motif change
rns-420	mt IIB1-LHE	*Ulva meridionalis*	ON402236	AAUUAAAA	AUGCGA	GC	Both 5′ and 3′ terminal motif change
rns-420	mt IIB1-LHE	*Ulva meridionalis*	ON402237	AAUUAAAA	AUGCGA	GC	Both 5′ and 3′ terminal motif change
rns-420	mt IIB1-LHE	*Ulva meridionalis*	ON402238	AAUUAAAA	AUGCGA	GC	Both 5′ and 3′ terminal motif change
rns-420	mt IIB1-LHE	*Ulva meridionalis*	ON402239	AAUUAAAA	AUGCGA	GC	Both 5′ and 3′ terminal motif change
rns-420	mt IIB1-LHE	*Ulva meridionalis*	ON402240	AAUUAAAA	AUGCGA	GC	Both 5′ and 3′ terminal motif change
rnl-2698	mt IIB1-LHE	*Ulva linza*	KU189740	UGCUUAAAA	ACGCGA	GU	Both 5′ and 3′ terminal motif change
rnl-2698	mt IIB1-LHE	*Ulva aragoënsis*	KX455878	GGCUUAAAA	AAGCGA	GU	Both 5′ and 3′ terminal motif change
rnl-2698	mt IIB1-LHE	*Ulva aragoënsis*	KY626326	GGCUUAAAA	AAGCGA	GU	Both 5′ and 3′ terminal motif change
rnl-2698	mt IIB1-LHE	*Ulva lacinulata*	MN389526	GGCUUGAAA	ACGCGA	GU	Both 5′ and 3′ terminal motif change
rnl-2698	mt IIB1-LHE	*Ulva* sp.	MN853878	GAAUUAAA	AAGCGA	GU	Both 5′ and 3′ terminal motif change
rnl-2698	mt IIB1-LHE	*Ulva meridionalis*	MN861072	GAAUUAAA	AAGCGA	AU	Both 5′ and 3′ terminal motif change
rnl-2698	mt IIB1-LHE	*Ulva lacinulata*	MT179357	GGCUUGAAA	ACGCGA	GU	Both 5′ and 3′ terminal motif change
rnl-2698	mt IIB1-LHE	*Ulva* sp. A AF-2021	MT179358	GGCUUGAAA	ACGCGA	GU	Both 5′ and 3′ terminal motif change
rnl-2698	mt IIB1-LHE	*Ulva intestinalis*	MZ571476	GGCUUGAAA	ACGCGA	GU	Both 5′ and 3′ terminal motif change
rnl-2698	mt IIB1-LHE	*Ulva taeniata*	PV023352	GGCUUGAAA	ACGCGA	GU	Both 5′ and 3′ terminal motif change
atp1-990	mt IIA1-RT/M	*Ulva ohnoi*	AP018695		GGGCGA	AU	Only 5′ terminal motif change
atp1-990	mt IIA1-RT/M	*Ulva* sp.	MN853878		GGGCGA	AU	Only 5′ terminal motif change
atp1-990	mt IIA1-RT/M	*Ulva meridionalis*	ON402236		GGGCGA	AU	Only 5′ terminal motif change
atp1-990	mt IIA1-RT/M	*Ulva meridionalis*	ON402237		GGGCGA	AU	Only 5′ terminal motif change
atp1-990	mt IIA1-RT/M	*Ulva meridionalis*	ON402238		GGGCGA	AU	Only 5′ terminal motif change
atp1-990	mt IIA1-RT/M	*Ulva meridionalis*	ON402239		GGGCGA	AU	Only 5′ terminal motif change

Intron name	Intron type	Species	GenBank accession number	5′ terminal linker (5′-3′)	5′ terminal motif (5-GUGCGA-3′)	3′ terminal motif (5′-AY-3′)	Changes of 5′ and 3′ terminal motifs (GUGCGA/AY)
atp1-990	mt IIA1-RT/M	*Ulva meridionalis*	ON402240		GGGCGA	AU	Only 5′ terminal motif change
cox1-760	mt IIA1-RT/M	*Ulva australis*	KX530816		GGGCGU	AU	Only 5′ terminal motif change
cox1-760	mt IIA1-RT/M	*Ulva aragoënsis*	MH013470		GGGCGU	AU	Only 5′ terminal motif change
cox1-760	mt IIA1-RT/M	*Ulva rigida*	MT179359		GGGCGU	AU	Only 5′ terminal motif change
nad5-1057	mt IIA1-RT/M	*Ulva fenestrata*	MT179355		GGGCGA	AC	Only 5′ terminal motif change
nad5-1057	mt IIA1-RT/M	*Ulva rigida*	MT179359		GGGCGA	AC	Only 5′ terminal motif change
cox1-643	mt IIA1-RT/M	*Ulva compressa*	MH013469		GGGCGA	AC	Only 5′ terminal motif change
cox1-643	mt IIA1-RT/M	*Ulva compressa*	MK069587		GGGCGA	AC	Only 5′ terminal motif change
cox1-643	mt IIA1-RT/M	*Ulva fenestrata*	MT179355		GGGCGA	AC	Only 5′ terminal motif change
atp1-1095	mt IIA1-RT/M	*Ulva rigida*	MT179359		GGGCGA	AU	Only 5′ terminal motif change
cox1-199	mt IIA1-RT/M	*Ulva linza*	KU189740		GUGCGC	AC	Only 5′ terminal motif change
cox1-199	mt IIA1-RT/M	*Ulva aragoënsis*	MH013470		GUGCGC	AC	Only 5′ terminal motif change
cox1-199	mt IIA1-RT/M	*Ulva compressa*	MK069586		GUGCGC	AC	Only 5′ terminal motif change
cox1-199	mt IIA1-RT/M	*Ulva* sp.	MN853878		GUGCGC	AC	Only 5′ terminal motif change
cox1-199	mt IIA1-RT/M	*Ulva taeniata*	OR030801		GUGCGC	AC	Only 5′ terminal motif change
cox2-424	mt IIA1-RT/M	*Ulva ohnoi*	AP018695		GUGCGC	AC	Only 5′ terminal motif change
cox2-424	mt IIA1-RT/M	*Ulva australis*	KX530816		GUGCGC	AC	Only 5′ terminal motif change
cox2-424	mt IIA1-RT/M	*Ulva compressa*	KX595276		GUGCGC	AC	Only 5′ terminal motif change
cox2-424	mt IIA1-RT/M	*Ulva compressa*	KY626327		GUGCGC	AC	Only 5′ terminal motif change
cox2-424	mt IIA1-RT/M	*Ulva compressa*	MH013469		GUGCGC	AC	Only 5′ terminal motif change
cox2-424	mt IIA1-RT/M	*Ulva torta*	MH013471		GUGCGC	AC	Only 5′ terminal motif change
cox2-424	mt IIA1-RT/M	*Ulva compressa*	MK069586		GUGCGC	AC	Only 5′ terminal motif change
cox2-424	mt IIA1-RT/M	*Ulva compressa*	MK069587		GUGCGC	AC	Only 5′ terminal motif change
cox2-424	mt IIA1-RT/M	*Ulva* sp.	MN853878		GUGCGC	AC	Only 5′ terminal motif change

Intron name	Intron type	Species	GenBank accession number	5′ terminal linker (5′-3′)	5′ terminal motif (5-GUGCGA-3′)	3′ terminal motif (5′-AY-3′)	Changes of 5′ and 3′ terminal motifs (GUGCGA/AY)
cox2-424	mt IIA1-RT/M	*Ulva fenestrata*	MT179355		GUGCGC	AC	Only 5′ terminal motif change
cox2-424	mt IIA1-RT/M	*Ulva gigantea*	MT179356		GUGCGC	AC	Only 5′ terminal motif change
cox2-424	mt IIA1-RT/M	*Ulva* sp. A AF-2021	MT179358		GUGCGC	AC	Only 5′ terminal motif change
cox2-424	mt IIA1-RT/M	*Ulva rigida*	MT179359		GUGCGC	AC	Only 5′ terminal motif change
cox2-424	mt IIA1-RT/M	*Ulva taeniata*	OR030801		GUGCGC	AC	Only 5′ terminal motif change
infA-62	cp IIB-like	*Ulva ohnoi*	AP018696		GUGUGA	AU	Only 5′ terminal motif change
infA-62	cp IIB-like	*Ulva* sp. UNA00071828	KP720616		GUGUGA	AU	Only 5′ terminal motif change
infA-62	cp IIB-like	*Ulva lactuca*	KT882614		GUGUGA	AU	Only 5′ terminal motif change
infA-62	cp IIB-like	*Ulva linza*	KX058323		GUGUGA	AU	Only 5′ terminal motif change
infA-62	cp IIB-like	*Ulva prolifera*	KX342867		GUGUGA	AU	Only 5′ terminal motif change
infA-62	cp IIB-like	*Ulva aragoënsis*	KX579943		GUGUGA	AU	Only 5′ terminal motif change
infA-62	cp IIB-like	*Ulva compressa*	KX595275		GUGUGA	AU	Only 5′ terminal motif change
infA-62	cp IIB-like	*Ulva australis*	LC507117		GUGUGA	AU	Only 5′ terminal motif change
infA-62	cp IIB-like	*Ulva lactuca*	MH730972		GUGUGA	AU	Only 5′ terminal motif change
infA-62	cp IIB-like	*Ulva compressa*	MK069584		GUGUGA	AU	Only 5′ terminal motif change
infA-62	cp IIB-like	*Ulva compressa*	MK069585		GUGUGA	AU	Only 5′ terminal motif change
infA-62	cp IIB-like	*Ulva lacinulata*	MN389525		GUGUGA	AU	Only 5′ terminal motif change
infA-62	cp IIB-like	*Ulva australis*	MN853875		GUGUGA	AU	Only 5′ terminal motif change
infA-62	cp IIB-like	*Ulva* sp.	MN853879		GUGUGA	AU	Only 5′ terminal motif change
infA-62	cp IIB-like	*Ulva* sp.	MN889540		GUGUGA	AU	Only 5′ terminal motif change
infA-62	cp IIB-like	*Ulva australis*	MT179348		GUGUGA	AU	Only 5′ terminal motif change
infA-62	cp IIB-like	*Ulva fenestrata*	MT179349		GUGUGA	AU	Only 5′ terminal motif change
infA-62	cp IIB-like	*Ulva gigantea*	MT179350		GUGUGA	AU	Only 5′ terminal motif change
infA-62	cp IIB-like	*Ulva lacinulata*	MT179351		GUGUGA	AU	Only 5′ terminal motif change

Intron name	Intron type	Species	GenBank accession number	5′ terminal linker (5′-3′)	5′ terminal motif (5-GUGCGA-3′)	3′ terminal motif (5′-AY-3′)	Changes of 5′ and 3′ terminal motifs (GUGCGA/AY)
infA-62	cp IIB-like	*Ulva* sp. A AF-2021	MT179352		GUGUGA	AU	Only 5′ terminal motif change
infA-62	cp IIB-like	*Ulva rigida*	MT179353		GUGUGA	AU	Only 5′ terminal motif change
infA-62	cp IIB-like	*Ulva compressa*	MT916929		GUGUGA	AU	Only 5′ terminal motif change
infA-62	cp IIB-like	*Ulva compressa*	MW344287		GUGUGA	AU	Only 5′ terminal motif change
infA-62	cp IIB-like	*Ulva compressa*	MW353781		GUGUGA	AU	Only 5′ terminal motif change
infA-62	cp IIB-like	*Ulva lacinulata*	MW531676		GUGUGA	AU	Only 5′ terminal motif change
infA-62	cp IIB-like	*Ulva rigida*	MW543060		GUGUGA	AU	Only 5′ terminal motif change
infA-62	cp IIB-like	*Ulva lacinulata*	MW543061		GUGUGA	AU	Only 5′ terminal motif change
infA-62	cp IIB-like	*Ulva compressa*	MW548841		GUGUGA	AU	Only 5′ terminal motif change
infA-62	cp IIB-like	*Ulva* sp. Q253	MW699788		GUGUGA	AU	Only 5′ terminal motif change
infA-62	cp IIB-like	*Ulva intestinalis*	MZ158703		GUGUGA	AU	Only 5′ terminal motif change
infA-62	cp IIB-like	*Ulva californica*	MZ561475		GUGUGA	AU	Only 5′ terminal motif change
infA-62	cp IIB-like	*Ulva prolifera*	MZ571508		GUGUGA	AU	Only 5′ terminal motif change
infA-62	cp IIB-like	*Ulva torta*	MZ703011		GUGUGA	AU	Only 5′ terminal motif change
infA-62	cp IIB-like	*Ulva tepida*	OL684341		GUGUGA	AU	Only 5′ terminal motif change
infA-62	cp IIB-like	*Ulva torta*	OL684342		GUGUGA	AU	Only 5′ terminal motif change
infA-62	cp IIB-like	*Ulva prolifera*	OP985129		GUGUGA	AU	Only 5′ terminal motif change
infA-62	cp IIB-like	*Ulva prolifera*	OP985130		GUGUGA	AU	Only 5′ terminal motif change
infA-62	cp IIB-like	*Ulva prolifera*	OP985131		GUGUGA	AU	Only 5′ terminal motif change
infA-62	cp IIB-like	*Ulva aragoënsis*	OP985132		GUGUGA	AU	Only 5′ terminal motif change
infA-62	cp IIB-like	*Ulva meridionalis*	OP985133		GUGUGA	AU	Only 5′ terminal motif change
infA-62	cp IIB-like	*Ulva taeniata*	OQ349516		GUGUGA	AU	Only 5′ terminal motif change
infA-62	cp IIB-like	*Ulva dactylifera*	OR003918		GUGUGA	AU	Only 5′ terminal motif change
infA-62	cp IIB-like	*Ulva intestinalis*	PQ777150		GUGUGA	AU	Only 5′ terminal motif change

Intron name	Intron type	Species	GenBank accession number	5′ terminal linker (5′-3′)	5′ terminal motif (5-GUGCGA-3′)	3′ terminal motif (5′-AY-3′)	Changes of 5′ and 3′ terminal motifs (GUGCGA/AY)
infA-62	cp IIB-like	*Ulva compressa*	PQ777151		GUGUGA	AU	Only 5′ terminal motif change
infA-62	cp IIB-like	*Ulva lactuca*	PQ824971		GUGUGA	AU	Only 5′ terminal motif change
infA-62	cp IIB-like	*Ulva prolifera*	PV023350		GUGUGA	AU	Only 5′ terminal motif change
infA-62	cp IIB-like	*Ulva* sp.	PV138240		GUGUGA	AC	Only 5′ terminal motif change
cox1-686	mt IIB2-LHE	*Ulva* sp.	MN853878	UAACAAAAA	CAGCGA	AC	Only 5′ terminal motif change
rnl-1963	mt IIB1-RT/M	*Ulva pertusa*	KX530816		GUGCGA	CU	Only 3′ terminal motif change
rnl-1963	mt IIB1-RT/M	*Ulva australis*	KX530817		GUGCGA	CU	Only 3′ terminal motif change
rnl-1963	mt IIB1-RT/M	*Ulva expansa*	MH730971		GUGCGA	CU	Only 3′ terminal motif change
rnl-1963	mt IIB1-RT/M	*Ulva australis*	MT179354		GUGCGA	CU	Only 3′ terminal motif change
rnl-1963	mt IIB1-RT/M	*Ulva gigantea*	MT179356		GUGCGA	CU	Only 3′ terminal motif change
rnl-1963	mt IIB1-RT/M	*Ulva taeniata*	OR030801		GUGCGA	CU	Only 3′ terminal motif change
atpB-627	cp IIB2-RT/M	*Ulva ohnoi*	AP018696		GUGCGA	CC	Only 3′ terminal motif change
atpB-627	cp IIB2-RT/M	*Ulva aragoënsis*	KX579943		GUGCGA	CC	Only 3′ terminal motif change
atpB-627	cp IIB2-RT/M	*Ulva compressa*	KX595275		GUGCGA	CC	Only 3′ terminal motif change
atpB-627	cp IIB2-RT/M	*Ulva compressa*	MK069584		GUGCGA	CC	Only 3′ terminal motif change
atpB-627	cp IIB2-RT/M	*Ulva australis*	MN853875		GUGCGA	CC	Only 3′ terminal motif change
atpB-627	cp IIB2-RT/M	*Ulva fenestrata*	MT179349		GUGCGA	CC	Only 3′ terminal motif change
atpB-627	cp IIB2-RT/M	*Ulva gigantea*	MT179350		GUGCGA	CC	Only 3′ terminal motif change
atpB-627	cp IIB2-RT/M	*Ulva rigida*	MT179353		GUGCGA	CC	Only 3′ terminal motif change
atpB-627	cp IIB2-RT/M	*Ulva compressa*	MT916929		GUGCGA	CC	Only 3′ terminal motif change
atpB-627	cp IIB2-RT/M	*Ulva compressa*	MW344287		GUGCGA	CC	Only 3′ terminal motif change
atpB-627	cp IIB2-RT/M	*Ulva compressa*	MW353781		GUGCGA	CC	Only 3′ terminal motif change
atpB-627	cp IIB2-RT/M	*Ulva lacinulata*	MW531676		GUGCGA	CC	Only 3′ terminal motif change
atpB-627	cp IIB2-RT/M	*Ulva rigida*	MW543060		GUGCGA	CC	Only 3′ terminal motif change

Intron name	Intron type	Species	GenBank accession number	5′ terminal linker (5′-3′)	5′ terminal motif (5-GUGCGA-3′)	3′ terminal motif (5′-AY-3′)	Changes of 5′ and 3′ terminal motifs (GUGCGA/AY)
atpB-627	cp IIB2-RT/M	*Ulva lacinulata*	MW543061		GUGCGA	CC	Only 3′ terminal motif change
atpB-627	cp IIB2-RT/M	*Ulva compressa*	MW548841		GUGCGA	CC	Only 3′ terminal motif change
orf185-47	cp IIB2-RT/M	*Ulva tepida*	OL684341		GUGCGA	CC	Only 3′ terminal motif change
petD-87	cp IIA2-RT/M	*Ulva compressa*	KX595275		GUGCGA	AU	No change
petD-87	cp IIA2-RT/M	*Ulva gigantea*	MT179350		GUGCGA	AU	No change
petD-87	cp IIA2-RT/M	*Ulva compressa*	MW344287		GUGCGA	AU	No change
petD-87	cp IIA2-RT/M	*Ulva compressa*	MW353781		GUGCGA	AU	No change
atpB-537	cp IIB2-RT/M	*Ulva meridionalis*	OP985133		GUGCGA	AC	No change
atpB-696	cp IIB2-RT/M	*Ulva ohnoi*	AP018696		GUGCGA	AC	No change
atpB-696	cp IIB2-RT/M	*Ulva aragoënsis*	KX579943		GUGCGA	AC	No change
atpB-696	cp IIB2-RT/M	*Ulva compressa*	MK069584		GUGCGA	AC	No change
atpB-696	cp IIB2-RT/M	*Ulva lacinulata*	MN389525		GUGCGA	AC	No change
atpB-696	cp IIB2-RT/M	*Ulva gigantea*	MT179350		GUGCGA	AC	No change
atpB-696	cp IIB2-RT/M	*Ulva lacinulata*	MT179351		GUGCGA	AC	No change
atpB-696	cp IIB2-RT/M	*Ulva rigida*	MT179353		GUGCGA	AC	No change
atpB-696	cp IIB2-RT/M	*Ulva lacinulata*	MW531676		GUGCGA	AC	No change
atpB-696	cp IIB2-RT/M	*Ulva rigida*	MW543060		GUGCGA	AC	No change
atpB-696	cp IIB2-RT/M	*Ulva lacinulata*	MW543061		GUGCGA	AC	No change
atpB-696	cp IIB2-RT/M	*Ulva compressa*	MW548841		GUGCGA	AC	No change
atpB-696	cp IIB2-RT/M	*Ulva californica*	MZ561475		GUGCGA	AC	No change
atpB-696	cp IIB2-RT/M	*Ulva prolifera*	MZ571508		GUGCGA	AC	No change
atpB-696	cp IIB2-RT/M	*Ulva torta*	MZ703011		GUGCGA	AC	No change
atpB-696	cp IIB2-RT/M	*Ulva torta*	OL684342		GUGCGA	AC	No change
atpB-696	cp IIB2-RT/M	*Ulva aragoënsis*	OP985132		GUGCGA	AC	No change

Intron name	Intron type	Species	GenBank accession number	5′ terminal linker (5′-3′)	5′ terminal motif (5-GUGCGA-3′)	3′ terminal motif (5′-AY-3′)	Changes of 5′ and 3′ terminal motifs (GUGCGA/AY)
atpB-696	cp IIB2-RT/M	*Ulva meridionalis*	OP985133		GUGCGA	AC	No change
petB-169	cp IIB1-RT/M	*Ulva rigida*	MT179353		GUGCGA	AU	No change
petB-169	cp IIB1-RT/M	*Ulva rigida*	MW543060		GUGCGA	AU	No change
petB-23	cp IIB2-RT/M	*Ulva rigida*	MT179353		GUGCGA	AC	No change
petB-23	cp IIB2-RT/M	*Ulva rigida*	MW543060		GUGCGA	AC	No change
petB-23	cp IIB2-RT/M	*Ulva meridionalis*	OP985133		GUGCGA	AC	No change
petB-23	cp IIB2-RT/M	*Ulva taeniata*	OQ349516		GUGCGA	AC	No change
petB-277	cp IIB1-RT/M	*Ulva rigida*	MT179353		GUGCGA	AU	No change
petB-277	cp IIB1-RT/M	*Ulva rigida*	MW543060		GUGCGA	AU	No change
petB-277	cp IIB1-RT/M	*Ulva lacinulata*	MW543061		GUGCGA	AU	No change
petB-277	cp IIB1-RT/M	*Ulva torta*	MZ703011		GUGCGA	AU	No change
petB-277	cp IIB1-RT/M	*Ulva torta*	OL684342		GUGCGA	AU	No change
petB-277	cp IIB1-RT/M	*Ulva meridionalis*	OP985133		GUGCGA	AU	No change
petB-277	cp IIB1-RT/M	*Ulva dactylifera*	OR003918		GUGCGA	AU	No change
petB-69	cp IIB2-RT/M	*Ulva ohnoi*	AP018696		GUGCGA	AC	No change
petB-69	cp IIB2-RT/M	*Ulva* sp. UNA00071828	KP720616		GUGCGA	AC	No change
petB-69	cp IIB2-RT/M	*Ulva lactuca*	KT882614		GUGCGA	AC	No change
petB-69	cp IIB2-RT/M	*Ulva compressa*	KX595275		GUGCGA	AC	No change
petB-69	cp IIB2-RT/M	*Ulva australis*	LC507117		GUGCGA	AC	No change
petB-69	cp IIB2-RT/M	*Ulva lactuca*	MH730972		GUGCGA	AC	No change
petB-69	cp IIB2-RT/M	*Ulva compressa*	MK069584		GUGCGA	AC	No change
petB-69	cp IIB2-RT/M	*Ulva lacinulata*	MN389525		GUGCGA	AC	No change
petB-69	cp IIB2-RT/M	*Ulva australis*	MT179348		GUGCGA	AC	No change
petB-69	cp IIB2-RT/M	*Ulva gigantea*	MT179350		GUGCGA	AC	No change

Intron name	Intron type	Species	GenBank accession number	5′ terminal linker (5′-3′)	5′ terminal motif (5-GUGCGA-3′)	3′ terminal motif (5′-AY-3′)	Changes of 5′ and 3′ terminal motifs (GUGCGA/AY)
petB-69	cp IIB2-RT/M	*Ulva lacinulata*	MT179351		GUGCGA	AC	No change
petB-69	cp IIB2-RT/M	*Ulva compressa*	MT916929		GUGCGA	AC	No change
petB-69	cp IIB2-RT/M	*Ulva compressa*	MW353781		GUGCGA	AC	No change
petB-69	cp IIB2-RT/M	*Ulva lacinulata*	MW531676		GUGCGA	AC	No change
petB-69	cp IIB2-RT/M	*Ulva lacinulata*	MW543061		GUGCGA	AC	No change
petB-69	cp IIB2-RT/M	*Ulva compressa*	MW548841		GUGCGA	AC	No change
petB-69	cp IIB2-RT/M	*Ulva intestinalis*	MZ158703		GUGCGA	AC	No change
petB-69	cp IIB2-RT/M	*Ulva prolifera*	MZ571508		GUGCGA	AC	No change
petB-69	cp IIB2-RT/M	*Ulva torta*	MZ703011		GUGCGA	AC	No change
petB-69	cp IIB2-RT/M	*Ulva torta*	OL684342		GUGCGA	AC	No change
petB-69	cp IIB2-RT/M	*Ulva dactylifera*	OR003918		GUGCGA	AC	No change
petB-69	cp IIB2-RT/M	*Ulva intestinalis*	PQ777150		GUGCGA	AC	No change
petB-69	cp IIB2-RT/M	*Ulva lactuca*	PQ824971		GUGCGA	AC	No change
cox2-751	mt IIB2-RT/M	*Ulva australis*	KX530816		GUGCGA	AC	No change
cox2-751	mt IIB2-RT/M	*Ulva australis*	KX530817		GUGCGA	AC	No change
cox2-751	mt IIB2-RT/M	*Ulva compressa*	KX595276		GUGCGA	AC	No change
cox2-751	mt IIB2-RT/M	*Ulva compressa*	KY626327		GUGCGA	AC	No change
cox2-751	mt IIB2-RT/M	*Ulva torta*	MH013471		GUGCGA	AC	No change
cox2-751	mt IIB2-RT/M	*Ulva compressa*	MH093740		GUGCGA	AC	No change
cox2-751	mt IIB2-RT/M	*Ulva compressa*	MK069586		GUGCGA	AC	No change
cox2-751	mt IIB2-RT/M	*Ulva compressa*	MK069587		GUGCGA	AC	No change
cox2-751	mt IIB2-RT/M	*Ulva* sp.	MN853878		GUGCGA	AC	No change
cox2-751	mt IIB2-RT/M	*Ulva australis*	MT179354		GUGCGA	AC	No change
cox2-751	mt IIB2-RT/M	*Ulva rigida*	MT179359		GUGCGA	AC	No change

Intron name	Intron type	Species	GenBank accession number	5′ terminal linker (5′-3′)	5′ terminal motif (5-GUGCGA-3′)	3′ terminal motif (5′-AY-3′)	Changes of 5′ and 3′ terminal motifs (GUGCGA/AY)
cox2-751	mt IIB2-RT/M	*Ulva meridionalis*	ON402236		GUGCGA	AC	No change
cox2-751	mt IIB2-RT/M	*Ulva meridionalis*	ON402237		GUGCGA	AC	No change
cox2-751	mt IIB2-RT/M	*Ulva meridionalis*	ON402238		GUGCGA	AC	No change
cox2-751	mt IIB2-RT/M	*Ulva meridionalis*	ON402239		GUGCGA	AC	No change
cox2-751	mt IIB2-RT/M	*Ulva meridionalis*	ON402240		GUGCGA	AC	No change
cox2-751	mt IIB2-RT/M	*Ulva taeniata*	OR030801		GUGCGA	AC	No change
nad3-215	mt IIB2-RT/M	*Ulva* sp. UNA00071828	KP720617		GUGCGA	AC	No change
nad3-216	mt IIB2-RT/M	*Ulva ohnoi*	AP018695		GUGCGA	AC	No change
nad3-216	mt IIB2-RT/M	*Ulva lactuca*	KT364296		GUGCGA	AC	No change
nad3-216	mt IIB2-RT/M	*Ulva prolifera*	KT428794		GUGCGA	AC	No change
nad3-216	mt IIB2-RT/M	*Ulva prolifera*	KU161104		GUGCGA	AC	No change
nad3-216	mt IIB2-RT/M	*Ulva lactuca*	KU182748		GUGCGA	AC	No change
nad3-216	mt IIB2-RT/M	*Ulva linza*	KU189740		GUGCGA	AC	No change
nad3-216	mt IIB2-RT/M	*Ulva compressa*	MH013469		GUGCGA	-	No change
nad3-216	mt IIB2-RT/M	*Ulva lactuca*	MH763013		GUGCGA	AC	No change
nad3-216	mt IIB2-RT/M	*Ulva compressa*	MK069586		GUGCGA	AC	No change
nad3-216	mt IIB2-RT/M	*Ulva compressa*	MK069587		GUGCGA	AC	No change
nad3-216	mt IIB2-RT/M	*Ulva gigantea*	MT179356		GUGCGA	AC	No change
nad3-216	mt IIB2-RT/M	*Ulva* sp. A AF-2021	MT179358		GUGCGA	AC	No change
nad3-216	mt IIB2-RT/M	*Ulva rigida*	MT179359		GUGCGA	AC	No change
nad3-216	mt IIB2-RT/M	*Ulva prolifera*	MZ438677		GUGCGA	AC	No change
nad3-216	mt IIB2-RT/M	*Ulva meridionalis*	ON402236		GUGCGA	AC	No change
nad3-216	mt IIB2-RT/M	*Ulva meridionalis*	ON402237		GUGCGA	AC	No change
nad3-216	mt IIB2-RT/M	*Ulva meridionalis*	ON402238		GUGCGA	AC	No change

Intron name	Intron type	Species	GenBank accession number	5′ terminal linker (5′-3′)	5′ terminal motif (5-GUGCGA-3′)	3′ terminal motif (5′-AY-3′)	Changes of 5′ and 3′ terminal motifs (GUGCGA/AY)
nad3-216	mt IIB2-RT/M	*Ulva meridionalis*	ON402239		GUGCGA	AC	No change
nad3-216	mt IIB2-RT/M	*Ulva meridionalis*	ON402240		GUGCGA	AC	No change
nad3-216	mt IIB2-RT/M	*Ulva dactylifera*	OR030800		GUGCGA	AC	No change
nad3-216	mt IIB2-RT/M	*Ulva taeniata*	OR030801		GUGCGA	AC	No change
nad3-216	mt IIB2-RT/M	*Ulva prolifera*	PV023351		GUGCGA	AC	No change
nad3-216	mt IIB2-RT/M	*Ulva taeniata*	PV023352		GUGCGA	AC	No change
rns-780	mt IIB1-RT/M	*Ulva ohnoi*	AP018695		GUGCGA	AU	No change
rns-780	mt IIB1-RT/M	*Ulva* sp. UNA00071828	KP720617		GUGCGA	AU	No change
rns-780	mt IIB1-RT/M	*Ulva pertusa*	KX530816		GUGCGA	AU	No change
rns-780	mt IIB1-RT/M	*Ulva australis*	KX530817		GUGCGA	AU	No change
rns-780	mt IIB1-RT/M	*Ulva compressa*	MK069587		GUGCGA	AU	No change
rns-780	mt IIB1-RT/M	*Ulva australis*	MT179354		GUGCGA	AU	No change
rnl-2080	mt IIB2-LHE	*Ulva aragoënsis*	KX455878		GUGCGA	AC	No change
rnl-2080	mt IIB2-LHE	*Ulva aragoënsis*	KY626326		GUGCGA	AC	No change
rnl-2080	mt IIB2-LHE	*Ulva expansa*	MH730971		GUGCGA	AC	No change
rnl-2080	mt IIB2-LHE	*Ulva lacinulata*	MN389526		GUGCGA	AC	No change
rnl-2080	mt IIB2-LHE	*Ulva* sp.	MN853878		GUGCGA	AC	No change
rnl-2080	mt IIB2-LHE	*Ulva meridionalis*	MN861072		GUGCGA	AC	No change
rnl-2080	mt IIB2-LHE	*Ulva lacinulata*	MT179357		GUGCGA	AC	No change
rnl-2080	mt IIB2-LHE	*Ulva* sp. A AF-2021	MT179358		GUGCGA	AC	No change
rnl-2080	mt IIB2-LHE	*Ulva taeniata*	PV023352		GUGCGA	AC	No change
rns-670	mt IIB2-LHE	*Ulva* sp.	KP720617		GUGCGA	AC	No change
rns-670	mt IIB2-LHE	*Ulva prolifera*	KT428794		GUGCGA	AC	No change
rns-670	mt IIB2-LHE	*Ulva prolifera*	KU161104		GUGCGA	AC	No change

Intron name	Intron type	Species	GenBank accession number	5′ terminal linker (5′-3′)	5′ terminal motif (5-GUGCGA-3′)	3′ terminal motif (5′-AY-3′)	Changes of 5′ and 3′ terminal motifs (GUGCGA/AY)
rns-670	mt IIB2-LHE	*Ulva linza*	KU189740		GUGCGA	AC	No change
rns-670	mt IIB2-LHE	*Ulva aragoënsis*	KX455878		GUGCGA	AC	No change
rns-670	mt IIB2-LHE	*Ulva aragoënsis*	KY626326		GUGCGA	AC	No change
rns-670	mt IIB2-LHE	*Ulva* sp. TM637	MH013467		GUGCGA	AC	No change
rns-670	mt IIB2-LHE	*Ulva aragoënsis*	MH013470		GUGCGA	AC	No change
rns-670	mt IIB2-LHE	*Ulva expansa*	MH730971		GUGCGA	AC	No change
rns-670	mt IIB2-LHE	*Ulva* sp.	MN853878		GUGCGA	AC	No change
rns-670	mt IIB2-LHE	*Ulva meridionalis*	MN861072		GUGCGA	AC	No change
rns-670	mt IIB2-LHE	*Ulva* sp. A AF-2021	MT179358		GUGCGA	AC	No change
rns-670	mt IIB2-LHE	*Ulva prolifera*	MZ438677		GUGCGA	AC	No change
rns-670	mt IIB2-LHE	*Ulva meridionalis*	ON402236		GUGCGA	AC	No change
rns-670	mt IIB2-LHE	*Ulva meridionalis*	ON402237		GUGCGA	AC	No change
rns-670	mt IIB2-LHE	*Ulva meridionalis*	ON402238		GUGCGA	AC	No change
rns-670	mt IIB2-LHE	*Ulva meridionalis*	ON402239		GUGCGA	AC	No change
rns-670	mt IIB2-LHE	*Ulva meridionalis*	ON402240		GUGCGA	AC	No change
rns-670	mt IIB2-LHE	*Ulva* sp.	PP908992		GUGCGA	AC	No change
rns-670	mt IIB2-LHE	*Ulva prolifera*	PV023351		GUGCGA	AC	No change
rns-420	mt IIB1-LHE	*Ulva linza*	KU189740		GUGCGA	AU	No change
rns-420	mt IIB1-LHE	*Ulva aragoënsis*	KX455878		GUGCGA	AU	No change
rns-420	mt IIB1-LHE	*Ulva aragoënsis*	KY626326		GUGCGA	AU	No change
rns-420	mt IIB1-LHE	*Ulva expansa*	MH730971		GUGCGA	AU	No change
rns-420	mt IIB1-LHE	*Ulva lacinulata*	MN389526		GUGCGA	AU	No change
rns-420	mt IIB1-LHE	*Ulva meridionalis*	MN861072		GUGCGA	AU	No change
rns-420	mt IIB1-LHE	*Ulva lacinulata*	MT179357		GUGCGA	AU	No change

Intron name	Intron type	Species	GenBank accession number	5′ terminal linker (5′-3′)	5′ terminal motif (5-GUGCGA-3′)	3′ terminal motif (5′-AY-3′)	Changes of 5′ and 3′ terminal motifs (GUGCGA/AY)
rns-420	mt IIB1-LHE	*Ulva* sp. A AF-2021	MT179358		GUGCGA	AU	No change
rns-420	mt IIB1-LHE	*Ulva intestinalis*	MZ571476		GUGCGA	AU	No change
rns-420	mt IIB1-LHE	*Ulva taeniata*	OR030801		GUGCGA	AU	No change
rns-420	mt IIB1-LHE	*Ulva taeniata*	PV023352		GUGCGA	AU	No change
